# Targeting L-arginine/NO/cGMP/K_ATP_ pathway by phytochemicals: therapeutic applications and clinical perspectives

**DOI:** 10.3389/fphar.2025.1708659

**Published:** 2025-11-28

**Authors:** Seyede Zahra Hosseini, Fatemeh Abbaszadeh, Seyede Darya Alavi, Mohammad Mehdi Gravandi, Roshanak Amirian, Sajad Fakhri, Javier Echeverría

**Affiliations:** 1 Student Research Committee, Kermanshah University of Medical Sciences, Kermanshah, Iran; 2 Neurobiology Research Center, Institute of Neuroscience and Cognition, Shahid Beheshti University of Medical Sciences, Tehran, Iran; 3 Pharmaceutical Sciences Research Center, Health Institute, Kermanshah University of Medical Sciences, Kermanshah, Iran; 4 Departamento de Ciencias del Ambiente, Facultad de Química y Biología, Universidad de Santiago de Chile, Santiago, Chile

**Keywords:** l-arginine, nitric oxide, cGMP, K_ATP_, cardiovascular disease, metabolic syndrome, pain, phytochemicals

## Abstract

**Background:**

The L-arginine/nitric oxide (NO)/cyclic guanosine monophosphate (cGMP)/ATP-sensitive potassium channel (K_ATP_) signaling pathway is a crucial biological pathway that plays a significant role in many physiological processes.

**Purpose:**

This study provides a comprehensive overview of this signaling pathway and its therapeutic implications across various diseases, including cardiovascular disorders, metabolic syndromes, and chronic pain conditions. It highlights how natural compounds can effectively influence this essential signaling cascade to help manage different health issues.

**Methodology:**

A comprehensive review was conducted to evaluate the effects of phytochemicals on modulating the L-arginine/NO/cGMP/K_ATP_ pathway in the treatment of cardiovascular disorders, metabolic syndromes, and chronic pain conditions. The literature review included searches of electronic databases such as Scopus®, PubMed®, and ScienceDirect®, as well as manual searches of reference lists and citations within the authors’ areas of expertise.

**Results and discussion:**

L-arginine plays a central role in this pathway, which is converted into NO—a potent vasodilator—by nitric oxide synthases, thereby regulating vascular tone and pain sensitivity. The subsequent activation of cGMP and K_ATP_ channels further influences cellular function, providing protective effects during ischemic events, supporting cardiovascular stability, helping combat metabolic syndromes, and pain. While traditional pharmacological approaches have shown effectiveness by regulating the pathway, they often come with undesirable side effects. Additionally, emerging evidence supports the role of phytochemicals as promising modulators with therapeutic potential in such conditions.

**Conclusion:**

Phytochemicals may modulate the L-arginine/NO/cGMP/K_ATP_ pathway to treat cardiovascular disorders, metabolic syndromes, and chronic pain conditions.

## 1 Introduction

The L-arginine/nitric oxide (NO)/cyclic guanosine monophosphate (cGMP)/ATP-sensitive potassium (K_ATP_) channel signaling pathway is a vital biological mechanism that has attracted significant attention in pharmacological research due to its diverse therapeutic roles. This pathway plays a crucial role in various physiological processes, including vasodilation, neurotransmission, metabolic syndrome, and pain modulation, making it a key target for therapeutic interventions. The interaction among these components regulates vascular tone, affects pain perception, and modulates inflammatory responses, providing a multifaceted approach to treat various conditions (NA, 2003; Ataei Ataabadi et al., 2020).

L-arginine, an amino acid that serves as a substrate for NO synthase (NOS), plays a critical role in the pathway. There are three main NO isoforms: neuronal NOS (nNOS), inducible NOS (iNOS), and endothelial NOS (eNOS). This enzyme catalyzes the conversion of L-arginine into NO, a potent endogenous vasodilator (Moncada, 1997; Andrabi et al., 2023). NO is a highly reactive free radical, enabling it to diffuse rapidly and easily through cell membranes. Consequently, it serves as a vital signaling molecule in numerous physiological and pathological processes, including vasodilation, antithrombotic properties, and anti-inflammatory actions (Sennequier and Vadon-Le Goff, 1998; Sharma et al., 2007). NO stimulates cGMP production by activating soluble guanylate cyclase (sGC). The increase in cGMP levels activates protein kinase G (PKG), which mediates downstream effects, including the opening of K_ATP_ channels. These channels play a critical role in cellular excitability and modulate pain pathways (Francis et al., 2010). Elevated cGMP subsequently results in vasodilation and improved blood flow, while also influencing other cellular functions such as apoptosis, inflammation, and platelet aggregation (Peixoto et al., 2017; Kremser, 2023). K_ATP_ channels, which are sensitive to intracellular ATP levels, also contribute to vascular smooth muscle relaxation and play a role in cardioprotection during ischemic events (Vishwakarma et al., 2019). The significance of this pathway extends beyond simple vasodilation; it is intricately linked to the mechanisms underlying nociception—the process by which painful stimuli are perceived (Bode-Böger et al., 1998; Levy and Zochodne, 2004; Schmidtko et al., 2009). Studies have shown that activation of the L-arginine/NO/cGMP/K_ATP_ pathway can produce antinociceptive effects, suggesting its potential as a target for pain management therapies (Schmidtko et al., 2009; Cury et al., 2011; Parvardeh et al., 2018).

Conventional pharmacological approaches, including phosphodiesterase (PDE) inhibitors and NO donors, have demonstrated efficacy in modulating this pathway; however, they often come with a range of side effects and limitations (ElHady et al., 2023). In recent years, there has been growing interest in the therapeutic potential of phytochemicals—bioactive compounds derived from plants—as modulators of the L-arginine/NO/cGMP/K_ATP_ pathway (Parvardeh et al., 2018; Rahemi et al., 2023). These compounds often exhibit anti-inflammatory, antioxidant, and analgesic properties, making them valuable in the treatment of various health conditions. These compounds are typically well tolerated, possess a wide safety margin, and exhibit multiple health benefits, making them attractive candidates for therapeutic intervention (Majid et al., 2015; Zhang et al., 2015; Zhu et al., 2018).

This review aims to provide a comprehensive overview of the phytochemicals targeting the L-arginine/NO/cGMP/K_ATP_ pathway, elucidating their mechanisms of action, therapeutic applications, and clinical perspectives in cardiovascular health, metabolic disorders, pain management, and other physiological functions.

## 2 The role of L-arginine/NO/cGMP/K_ATP_ pathway in various diseases

The L-arginine/NO/cGMP/K_ATP_ channel pathway plays a significant role in various physiological and pathological processes, particularly in metabolic disorders, pain modulation, and cardiovascular health. This pathway is crucial for regulating vascular tone, neuronal excitability, and pain perception, making it a focal point in understanding various diseases.

### 2.1 Cardiovascular diseases

L-arginine is an amino acid that acts as the primary substrate for NOS, which transforms it into NO and L-citrulline. This conversion is vital for maintaining endothelial function (Gambardella et al., 2020). In the cardiovascular system, NO serves as a critical signaling molecule involved in numerous regulatory processes, including facilitating vasodilation, decreasing leukocyte adhesion, promoting hemostasis, inhibiting platelet aggregation, facilitating fibrinolysis, regulating vascular smooth muscle cell proliferation, and maintaining blood pressure homeostasis (Mancini et al., 2011).

Under physiological conditions, NO is mainly produced by eNOS in endothelial cells lining blood vessels. Once synthesized, NO diffuses into vascular smooth muscle cells, where it activates sGC. This activation increases cGMP levels, promoting smooth muscle relaxation and vasodilation, thereby reducing vascular resistance and lowering blood pressure (Strijdom et al., 2009). In addition to its vasodilator effects, NO inhibits platelet aggregation, preventing platelets from clumping together—a vital function for maintaining normal blood flow and preventing thrombosis. This inhibitory effect is mediated by a rise in cGMP levels in platelets, which lowers intracellular calcium concentrations required for platelet activation (Liu and Huang, 2008; Strijdom et al., 2009).

NO also minimizes leukocyte adhesion to the endothelium by inhibiting the expression of adhesion molecules such as intercellular adhesion molecule-1 (ICAM-1) and vascular cell adhesion molecule-1 (VCAM-1). This action helps to reduce inflammation and the recruitment of immune cells to sites of injury or infection, thereby supporting cardiovascular health (Roy et al., 2023). Furthermore, NO plays a significant role in hemostasis by balancing clot formation and dissolution, promoting fibrinolysis—the process that breaks down clots—thereby preventing excessive clotting that could lead to vascular occlusion (Naseem, 2005). Additionally, NO maintains vascular smooth muscle cells in a non-proliferative state, which is crucial for preventing conditions like atherosclerosis. By inhibiting smooth muscle cell proliferation, NO helps preserve blood vessel integrity and prevents pathological remodeling. Overall, through its vasodilatory effects and interactions with various cellular processes, adequate levels of NO are vital for counteracting vasoconstrictive factors such as angiotensin II and endothelin-1 (ET-1), thereby contributing to overall cardiovascular stability (Naseem, 2005; Carlström et al., 2024).

In conditions such as hypertension and atherosclerosis, NO production is frequently diminished due to increased arginase activity, an enzyme that competes with NOS for L-arginine. This competition reduces the availability of L-arginine for NO synthesis, resulting in endothelial dysfunction characterized by impaired vasodilation and increased vascular resistance (Sun et al., 2019; Gambardella et al., 2020). Endothelial dysfunction is evident through a diminished capacity to regulate vascular tone and elevated levels of reactive oxygen species (ROS), which contribute to chronic inflammation and vascular damage. The imbalance between NO-mediated vasodilation and vasoconstriction can worsen conditions like hypertension and atherosclerosis (Sun et al., 2019). Furthermore, elevated levels of asymmetric dimethylarginine (ADMA), an endogenous inhibitor of NOS, further complicate this scenario by reducing NO bioavailability (Raghavan and Dikshit, 2004; Gambardella et al., 2020).

In heart failure, NO’s role extends beyond vasodilation; it also influences myocardial contractility and relaxation. Impaired NO signaling can disrupt cardiac function by lowering cGMP levels, which are essential for smooth muscle relaxation and proper heart filling. This disruption can intensify heart failure symptoms by negatively affecting both diastolic and systolic function (Divakaran and Loscalzo, 2017). During ischemia-reperfusion events, NO exerts significant protective effects. NO aids vasodilation, helping restore blood flow to ischemic tissues while mitigating oxidative stress. This function is critical for reducing cell death during reperfusion. Additionally, the opening of K_ATP_ channels is another way in which NO provides protective effects, promoting cell survival in ischemic conditions (Cziráki et al., 2020).

**FIGURE 1:**
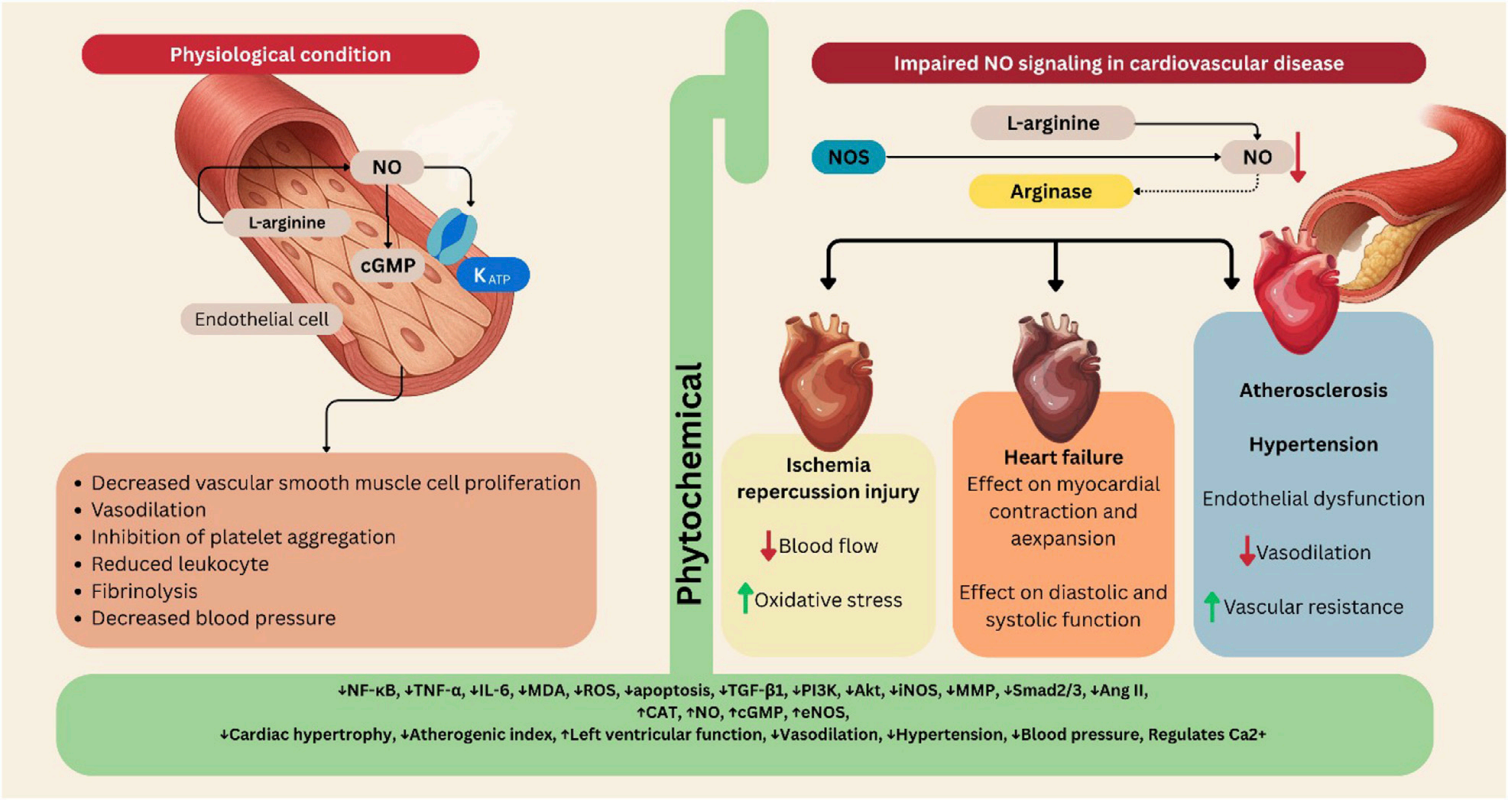
The L-arginine/NO/cGMP/K_ATP_ pathway in cardiovascular diseases, and its modulation by phytochemicals. The figure illustrates how the L-arginine/NO/cGMP/K_ATP_ pathway regulates vascular and cardiac function under physiological and pathological conditions. Left panel (Physiological condition): In endothelial cells, L-arginine is converted by NOS to NO, which activates cGMP signaling and K_ATP_ channels. The pathway results in vasodilation, inhibition of platelet aggregation, reduced leukocyte adhesion, fibrinolysis, and decreased vascular smooth muscle cell proliferation and blood pressure. Right panel (Impaired NO signaling in cardiovascular disease): Under pathological conditions such as atherosclerosis, hypertension, and heart failure, NO synthesis or bioavailability is reduced, often due to increased arginase activity or oxidative stress. This leads to endothelial dysfunction, impaired vasodilation, elevated vascular resistance, and ischemia–reperfusion injury. Central section (Phytochemical modulation): Various plant-derived phytochemicals enhance NO production, restore eNOS activity, reduce oxidative stress, and modulate downstream signaling. These effects collectively improve vasodilation, decrease blood pressure, and protect against cardiac hypertrophy, ischemic injury, and heart failure. Akt: Protein kinase B (PKB), Ang II: Angiotensin II, CAT: Catalase, cGMP: Cyclic Guanosine Monophosphate, eNOS: Endothelial Nitric Oxide Synthase, IL-6: Interleukin 6, iNOS: Inducible Nitric Oxide Synthase, MDA: Malondialdehyde, MMP: Matrix Metalloproteinase, NF-κB: Nuclear Factor Kappa B, NO: Nitric Oxide, PI3K: Phosphoinositide 3-kinase, ROS: Reactive Oxygen Species, Smad2/3: SMAD family member 2 and 3, TGF-β1: Transforming Growth Factor beta 1, TNF-α: Tumor Necrosis Factor alpha. Figures were Created by Canva.

Therefore, the interplay between L-arginine metabolism, NO production, and signaling pathways is crucial for maintaining cardiovascular health. When there is a sufficient supply of L-arginine and optimal NOS activity, NO levels are adequately maintained, supporting endothelial function and vascular homeostasis. However, when this balance and these pathways are disrupted by factors such as inflammation, oxidative stress, or elevated arginase activity, the resulting decrease in NO can exacerbate cardiovascular diseases, including endothelial dysfunction, heart failure, and ischemia-reperfusion injury. Gaining insight into these mechanisms can identify potential therapeutic targets to improve vascular health and manage cardiovascular diseases (Figure 1).

### 2.2 Metabolic disorders

Metabolic disorders, including diabetes and obesity, are conditions characterized by abnormalities in the body’s metabolic processes, leading to issues like insulin resistance, increased fat accumulation, and heightened cardiovascular risk (Rochlani et al., 2017). Understanding the role of the L-arginine/NO/cGMP/K_ATP_ pathway can provide insights into potential therapeutic strategies for these disorders.

In addition to its other roles, NO is also considered a central regulator of energy metabolism and body composition. In obesity, NO bioavailability is often reduced, contributing to insulin resistance and increased adiposity. Studies have shown that enhancing NO production can improve insulin sensitivity and reduce obesity-related complications (Sansbury and Hill, 2014). NO plays a vital role in mitochondrial biogenesis and function. It influences mitochondrial respiration and apoptosis, which are crucial for maintaining energy homeostasis (Litvinova et al., 2015). NO promotes mitochondrial biogenesis primarily by activating key transcriptional regulators, including peroxisome proliferator-activated receptor gamma coactivator 1-alpha (PGC-1α), nuclear respiratory factor (Nrf-1), nuclear factor erythroid 2 -related factor 2 (Nrf-2), and mitochondrial transcription factor A (TFAM). These factors are critical for the expression of genes involved in mitochondrial proliferation and function (Nisoli and Carruba, 2006).

NO frequently exerts its effects via cGMP, which mediates the expression of genes related to mitochondrial biogenesis (Brown, 2007). As a potent vasodilator, NO improves blood flow and oxygen delivery to tissues, indirectly supporting mitochondrial function by ensuring an adequate supply of respiratory substrates (Valerio and Nisoli, 2015). Reduced eNOS activity leads to decreased NO production, which is associated with mitochondrial dysfunction. This reduction can exacerbate metabolic disorders such as obesity and type 2 diabetes (T2DM). Studies indicate that eNOS-deficient mice exhibit impaired mitochondrial biogenesis and reduced ATP levels across various tissues, highlighting the importance of NO in maintaining mitochondrial health (Nisoli and Carruba, 2006; Litvinova et al., 2015). In diabetes, particularly T2DM, reduced NO bioavailability is linked to impaired insulin signaling pathways. Restoring NO levels has been shown to enhance insulin sensitivity and improve glucose metabolism in diabetic models (Pitocco et al., 2010; Sansbury and Hill, 2014).

Also, NO has been found to inhibit lipolysis in adipose tissue. In obese models, iNOS is often upregulated, leading to increased NO production that may contribute to insulin resistance by altering lipid metabolism (Elizalde et al., 2000). Studies highlighted the involvement of cGMP in the differentiation and function of brown adipose tissue, which is responsible for thermogenesis—burning energy to produce heat. Enhancing cGMP signaling can promote the recruitment of brown adipocytes, making it a potential target for anti-obesity therapies that increase energy expenditure in overweight individuals (Hoffmann et al., 2016; Reverte-Salisa et al., 2019). Research indicates that natriuretic peptides, which activate cGMP pathways, can enhance glucose uptake in adipocytes. This effect is notably reduced in adipocytes from obese individuals, suggesting that impaired cGMP signaling may contribute to the insulin resistance observed in T2DM. The expression of guanylyl cyclase-A (GC-A), a receptor for natriuretic peptides, correlates negatively with markers of insulin resistance, reinforcing the link between cGMP signaling and glucose metabolism (Coué et al., 2018). In addition, a study examining urinary excretion of cGMP found an inverse relationship between cGMP levels and components of metabolic syndrome. Lower urinary cGMP excretion was associated with a greater number of metabolic syndrome features, suggesting a potential role for cGMP as a biomarker of metabolic dysfunction (Cui et al., 2007).

**FIGURE 2:**
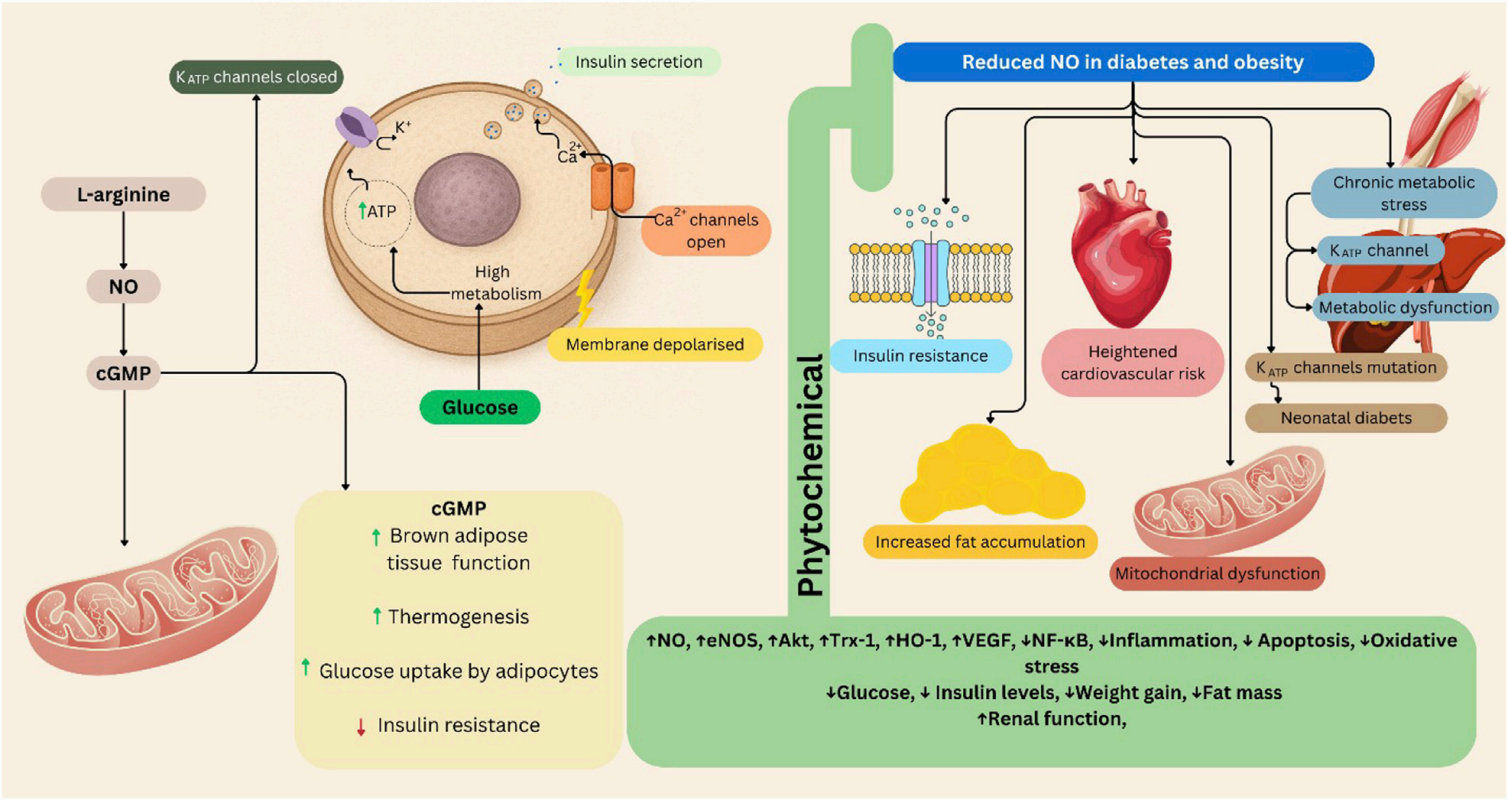
The L-arginine/NO/cGMP/K_ATP_ pathway in obesity and diabetes, and the role of phytochemical interventions. This schematic illustrates the role of the L-arginine/NO/cGMP/K_ATP_ signaling pathway in glucose metabolism, insulin secretion, and metabolic dysfunction. Left panel (Physiological condition): Under normal metabolic conditions, L-arginine-derived NO activates cGMP signaling, which regulates K_ATP_ channel activity in pancreatic β-cells and mitochondria. This enhances glucose uptake by adipocytes, stimulates thermogenesis in brown adipose tissue, and promotes insulin secretion following membrane depolarization and Ca^2+^ influx. These processes collectively improve insulin sensitivity and energy homeostasis. Right panel (Reduced NO in diabetes and obesity): In metabolic diseases such as obesity and type 2 diabetes, NO bioavailability is diminished due to oxidative stress and chronic inflammation. This impairs cGMP signaling, leading to insulin resistance, mitochondrial dysfunction, fat accumulation, and K_ATP_ channel abnormalities that contribute to neonatal diabetes and increased cardiovascular risk. Central section (Phytochemical modulation): Phytochemicals restore NO signaling and modulate eNOS and molecular pathways, thereby improving glucose utilization, reducing fat mass and oxidative stress, and enhancing mitochondrial and renal function. These effects counteract metabolic and cardiovascular complications associated with obesity and diabetes. Akt: Protein kinase B, cGMP: Cyclic Guanosine Monophosphate, eNOS: Endothelial Nitric Oxide Synthase, HO-1: Heme Oxygenase-1, NF-κB: Nuclear Factor kappa B, NO: Nitric Oxide, Trx-1: Thioredoxin-1, VEGF: Vascular Endothelial Growth Factor. Figures were Created by Canva.

K_ATP_ channels are integral to the function of pancreatic beta cells, regulating insulin secretion in response to glucose levels. When blood glucose rises, glucose metabolism increases ATP production, leading to the closure of K_ATP_ channels. This closure results in membrane depolarization, which opens voltage-gated calcium channels and promotes insulin granule exocytosis (Minami et al., 2004; Ashcroft, 2023). Conversely, impaired regulation of these channels can lead to insufficient insulin secretion, contributing to the development of T2DM. Specifically, mutations in K_ATP_ channel genes can cause neonatal diabetes by altering the channel’s response to ATP (Olson and Terzic, 2010; Ashcroft, 2023). Moreover, chronic metabolic stress associated with obesity can activate K_ATP_ channels in various tissues, including skeletal muscle and liver. This activation can impair cellular excitability and contractility, contributing to metabolic dysfunction. The interplay between K_ATP_ channel activity and insulin signaling pathways highlights their potential role in the pathophysiology of obesity (Kim H. J. et al., 2024) (Figure 2).

By understanding the intricate relationships between these pathways and obesity and diabetes, researchers can identify novel therapeutic strategies that may improve patient outcomes.

### 2.3 Pain management

Pain is a complex and multifaceted experience that involves intricate signaling pathways within the nervous system (Cao et al., 2024). Among these pathways, the L-arginine/NO/cGMP/K_ATP_ pathway has gained significant attention for its critical role in pain modulation. Understanding this pathway is essential for revealing the mechanisms underlying both acute and chronic pain and for developing new therapeutic strategies. The first step in this signaling pathway is the conversion of L-arginine in neurons and glial cells to NO by NOS. In the peripheral nervous system, NO enhances the transmission of pain signals by acting on primary nociceptive neurons. The presence of inflammatory mediators, such as cytokines, upregulates iNOS, resulting in increased NO production, which sensitizes nociceptors and contributes to pain sensation and hyperalgesia (Ong et al., 2021). In the central nervous system, NO produced by nNOS is critically dependent on calcium ion influx through N-methyl-D-aspartate (NMDA) receptors. Activation of NMDA receptors allows calcium to enter neurons, increasing intracellular calcium levels, which is essential for the central sensitization process (Cury et al., 2011). Also, NO plays a role in long-term potentiation (LTP), a process that enhances synaptic strength and is believed to be a cellular mechanism underlying learning and memory, as well as chronic pain states (Haley et al., 1992; Zhuo, 2014).

The release of neuropeptides involved in nociception (the sensory perception of pain) by NO is often mediated by sGC activation and the subsequent increase in cGMP levels (Kuhn, 2016). Elevated cGMP can increase intracellular calcium levels, which are crucial for neurotransmitter and neuropeptide release. cGMP signaling interacts with other second messengers, such as cyclic adenosine monophosphate (cAMP), creating a network of signaling pathways that can either potentiate or inhibit pain signals (Song et al., 2006; Li et al., 2019). For instance, NO can promote the release of neuropeptides, such as substance P and calcitonin gene-related peptide (CGRP), which are involved in pain signaling (Yonehara and Yoshimura, 1999; Bellamy et al., 2006). Once released, substance P acts on neurokinin-1 (NK1) receptors located on neurons in the spinal cord and brain, leading to enhanced pain perception (hyperalgesia) and the development of central sensitization. This means that even normal stimuli can be perceived as painful (allodynia), contributing to the persistence and amplification of pain (Yan, 2007; Vink, 2025). CGRP is another neuropeptide that increases neurons’ sensitivity to painful stimuli and contributes to the development of neurogenic inflammation. It promotes vasodilation and increases blood flow to the affected area, which can exacerbate swelling and pain. CGRP is also involved in the transmission of pain signals in the dorsal horn of the spinal cord, where it can influence synaptic transmission and plasticity (Schou et al., 2017).

**FIGURE 3:**
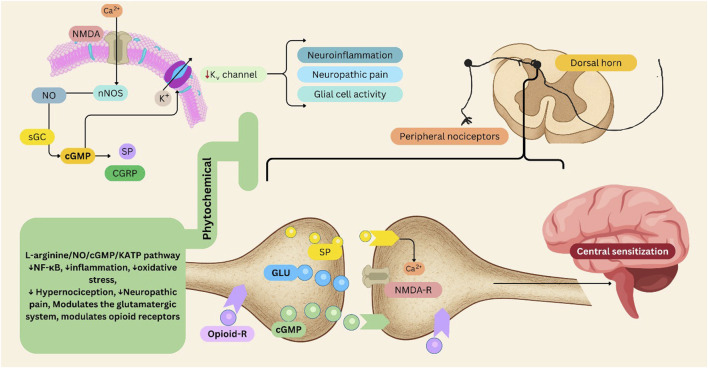
L-arginine/NO/cGMP/K_ATP_ pathway in pain management, its modulation by phytochemicals. This figure illustrates the involvement of the L-arginine/NO/cGMP/K_ATP_ pathway in pain, and the modulatory role of phytochemicals. Activation of nNOS via NMDA receptor–mediated Ca^2+^ influx stimulates NO production. NO activates sGC, increasing cGMP levels, which influence K_ATP_ channel activity. This cascade modulates neurotransmitter release—including GLU, SP, and CGRP—and contributes to neuronal hyperpolarization and analgesic responses. Dysregulation of this pathway contributes to neuroinflammation, glial activation, and central sensitization within the dorsal horn and brain regions involved in pain perception. Peripheral nociceptor sensitization and enhanced glutamatergic transmission exacerbate neuropathic pain. Phytochemical modulation: Phytochemicals can attenuate NF-κB activation, oxidative stress, and inflammatory signaling, thereby reducing hypernociception and neuropathic pain. They may also modulate the glutamatergic system and enhance opioid receptor–mediated antinociception, offering potential non-opioid strategies for pain management. cGMP: Cyclic Guanosine Monophosphate, CGRP: Calcitonin Gene-Related Peptide, NF-κB: Nuclear Factor Kappa B, NMDA: N-Methyl-D-Aspartate, NO: Nitric Oxide, nNOS: Neuronal Nitric Oxide Synthase, sGC: Soluble Guanylate Cyclase, SP: Substance P. Figures were Created by Canva.

The interplay between the NO/cGMP signaling pathway and K_ATP_ channels is crucial for effective pain modulation. K_ATP_ channels typically help regulate neuronal excitability by allowing potassium efflux, which hyperpolarizes the cell membrane and limits the generation of action potentials. In neuropathic pain states, the dysfunction or reduced expression of these channels can lead to spontaneous action potential generation in injured neurons or ectopic sites, further exacerbating pain signaling. This hyperexcitability is driven by changes in ion channel expression and function following nerve damage (Tsantoulas and McMahon, 2014; De Campos Lima et al., 2022). K_ATP_ channels also modulate neuroinflammatory responses associated with neuropathic pain. Activation of these channels can influence glial cell activity and reduce the release of pro-inflammatory mediators, potentially alleviating pain. Following nerve injury, K_ATP_ channels are often downregulated in dorsal root ganglion and spinal cord neurons, contributing to hyperexcitability and the development of neuropathic pain (Zhu et al., 2015). Activation of K_ATP_ channels using openers, such as pinacidil, can alleviate mechanical allodynia associated with various forms of neuropathic pain. These K_ATP_ channel openers induced analgesic effects by activating specific signaling pathways, such as the growth arrest-specific 6 (Gas6)/Axl/suppressor of cytokine signaling 3 (SOCS3) pathway in microglia, which helps attenuate neuroinflammation and postoperative pain (Zhu et al., 2015; Qian et al., 2023) (Figure 3).

Given the significant implications of the L-arginine/NO/cGMP/K_ATP_ pathway in various diseases, it becomes essential to explore innovative approaches that target these mechanisms for therapeutic benefit. Phytochemicals, naturally occurring compounds found in plants, have emerged as promising candidates for modulating this pathway due to their diverse biological activities and capacity to influence cellular signaling processes. By enhancing NO production, influencing cGMP levels, and regulating K_ATP_ channel activity, phytochemicals can potentially mitigate the adverse effects of diseases such as cardiovascular disorders, metabolic syndromes, and chronic pain conditions (Figure 3).

## 3 L-arginine/NO/cGMP/K_ATP_ pathway as a hopeful target for phytochemicals in various diseases

Medicinal plants have been integral to human health for thousands of years, serving as a primary source for the development of pharmaceuticals. The reliance on these plants has persisted into modern times, particularly in developing countries where access to conventional medicine is limited. The World Health Organization (WHO) estimates that over 80% of the population in these regions utilizes traditional medicine, much of which is derived from medicinal plants (Subedi et al., 2021). Today, medicinal plants remain a vital resource for drug discovery. Phytochemicals are a diverse range of compounds produced by plants that serve protective roles and are associated with numerous health benefits. They can be found in various sources, including fruits, vegetables, whole grains, nuts, and herbs. Key classes of phytochemicals include polyphenols, carotenoids, phytosterols, isoprenoids, and saponins. These compounds possess potent antioxidant properties and have been linked to antimicrobial, anti-inflammatory, and anticancer effects (Leitzmann, 2016; Kumar et al., 2023). These bioactive compounds have garnered attention for their potential therapeutic effects across various diseases (Nyamai et al., 2016; Pawase et al., 2024).

Many phytochemicals exhibit antioxidant properties that help to increase NO bioavailability. For instance, polyphenols can elevate NO levels by reducing oxidative stress and improving endothelial function. This action not only increases NO availability but also protects against ROS-mediated degradation (Kooshki and Hoseini, 2014; Malekmohammad et al., 2020).

### 3.1 Phytochemicals and phytoextracts modulating the L-arginine/NO/cGMP/K_ATP_ pathway for the treatment of cardiovascular diseases

Cardiovascular diseases remain a leading cause of morbidity and mortality worldwide, necessitating innovative therapeutic strategies to enhance vascular health and function (Chong et al., 2024). As mentioned, the L-arginine/NO/cGMP/K_ATP_ signaling pathway plays a pivotal role in regulating vascular tone, promoting vasodilation, and maintaining cardiovascular homeostasis. Dysregulation of this pathway is implicated in various cardiovascular conditions, including hypertension, atherosclerosis, and heart failure. Recent research has highlighted the potential of naturally derived compounds to modulate this essential signaling cascade. By enhancing NO production, promoting cGMP signaling, and activating K_ATP_ channels, these natural compounds may offer promising therapeutic avenues for the prevention and treatment of cardiovascular diseases (Forte et al., 2016) (Figure 1).

#### 3.1.1 Phytochemicals

The polyphenols, especially flavonoids, are abundant antioxidants in human diets, primarily found in plant-based foods and linked to the Mediterranean diet, which is associated with lower cardiovascular disease mortality. Their beneficial effects stem from their ability to scavenge free radicals, reduce oxidized low-density lipoprotein (LDL), induce NO production, and modulate inflammatory responses (Ginter and Simko, 2012). Several studies on phytochemicals have targeted the L-arginine/NO/cGMP/K_ATP_ pathway for the treatment of cardiovascular diseases (Table 1).

**TABLE 1:** Phytochemicals modulating the L-arginine/NO/cGMP/K_ATP_ pathway in cardiovascular disease.

Compound	Class	Method	*In vitro*/*in vivo* models	Mechanism and outcome	References
17-methoxyl-7-hydroxy-benzene-furanchalcone (MHBFC)	Flavonoid	Cardiac remodeling by abdominal aorta constriction	*In vivo:* Rats	↑Phosphorylation of PI3K, Akt, and eNOS, ↑eNOS enzyme activity, ↑Protective NO production, Activates eNOS-NO signaling pathway ↓Apoptosis of MMVECs	Ye et al. (2019)
3-hydroxyphenylacetic acid	Monocarboxylic acid	Acetaldehyde-induced cytotoxicity	*In vitro:* Mouse hepatoma Hepa1c1c7 and human hepatoma HepG2 cells	↑Cellular protection, ↑AhR-dependent reporter gene expression, ↑Total ALDH	Liu et al. (2022)
		Spontaneously hypertensive rats (SHR)	*In vivo:* SHR, *Ex vivo:* Precontracted porcine coronary artery segments	↓Blood pressure	Dias et al. (2022)
Apigenin	Flavonoid	Vasodilation	*In vivo:* SHRs	↑NO	Klider et al. (2024a)
Astragaloside IV	Triterpene	Vascular protection study	*In vitro:* Rat aortic rings	↑Vasodilation, ↑NO, ↑PI3K/Akt/eNOS pathway activation	Lin et al. (2018)
Baicalein	Flavonoid	Ischemia-reperfusion injury	*In vitro:* Cardiomyocytes	↑NO, ↑PTEN/Akt/NO pathway activation ↓ROS	Chang et al. (2019)
Berberine	Alkaloid	Hyperglycemia-induced endothelial dysfunction	*In vitro:* Mouse aortic rings, endothelial cells	↑eNOS phosphorylation, ↓Oxidative stress, ↓AMPK activation	Wang et al. (2009b)
Boldine	Alkaloid	Vasodilation in renal arteries	*In vitro:* Perfused rat kidneys	↑Vasodilation, ↑NO production	de Souza et al. (2022)
Bornesitol	Cyclitol	Blood pressure study	*In vivo*: Rats	↑NO production ↓ACE activity ↓Blood pressure	Moreira et al. (2019)
Caffeine and caffeic acid	Alkaloid and phenolic compound	Hypertension	*In vivo:* Rats	↑NO ↓Ang II	Oboh et al. (2021)
Chlorogenic acid	Phenolic compound	Oxidative stress	*In vitro*: Human aortic 4endothelial cells	↑NO ↑HO-1	Jiang et al. (2016)
Cinnamic acid and cinnamaldehyde	Phenolic compounds	Acute myocardial ischemia	*In vivo:* Rats	↑NO ↓CK, ↓LDH, ↓TNF-α, ↓IL-6	Song et al. (2013)
Curcumin	Polyphenol	Hypertension	*In vivo:* Rats	↑NO ↓Oxidative stress	Boonla et al. (2014)
		Homocysteine-induced endothelial dysfunction	*Ex vivo*: Porcine coronary artery rings	↓Homocysteine-induced endothelial dysfunction	Ramaswami et al. (2004)
Delphinidin	Anthocyanin	Apoptosis induction	*In vitro:* Bovine aortic endothelial cells	↑NOS, ↑Guanylyl cyclase, ↑MAPK activation	Martin et al. (2003)
Ellagic acid	Polyphenol	Hypertension (OVX-induced)	*In vivo:* Rats	↑eNOS phosphorylation, ↓Oxidative stress	da Silva et al. (2022)
(−)-Epicatechin	Flavonoid	Ischemia-reperfusion injury	*In vivo:* Rats, neonatal rat ventricular myocytes	↑NO, ↑Mitochondrial function ↓Infarct size	Yamazaki et al. (2014)
Epicatechin	Flavonoid	Vascular smooth muscle contraction	*In vitro:* Rat arteries	↑Vasodilation	MacRae et al. (2019)
Epigallocatechin gallate (EGCG)	Polyphenol	Vasodilation and NO production	*In vivo:* SHR rats *In vitro:* Endothelial cells	↑Fyn/PI3K/Akt/eNOS activity, ↑Cardiovascular health ↓ET-1	Nomura et al. (2017)
	Polyphenol	L-NAME induction	*Ex vivo:* WKY rats mesenteric vascular beds	↑ Vasorelaxation, ↑NO, ↑eNOS phosphorylation, ↑Fyn-dependent activation of PI3K/Akt/eNOS pathway, ↑Intracellular H_2_O_2_ generation	Kim et al. (2007)
Equol	Polyphenols	Atherosclerosis, estrogen replacement therapy	*In vitro:* Human endothelial cells *In vivo:* Mice	↑NO ↓LDL oxidation	De Andrade et al. (2015)
		Experimental study investigating the antioxidant properties	*In vitro:* J774 monocyte/macrophages	↓ LDL oxidation, ↑NO, ↑Cardiovascular protection ↓Superoxide radical production	Hwang et al. (2003)
Ginsenoside Rd	Triterpene Saponin	Nicotine-induced vascular damage	*In vitro:* HUVEC *In vivo:* Rats	↑NO ↓Inflammation	Zhang et al. (2020a)
Ginsenoside Rg3	Triterpene Saponin	Anoxia-reoxygenation injury induction	*In vivo:* Rat model of myocardial ischemia/reperfusion injury *In vitro:* Neonatal rat cardiomyocytes	↑p-Akt, ↑eNOS, ↑Bcl-2/Bax ↓Caspase-3, ↓Caspase-9, ↓NRC apoptosis	Wang et al. (2015)
Gomisin J	Lignan	Vasodilatory study	*In vitro:* Rat thoracic aorta	↑vasorelaxation via NO and guanylyl cyclase pathways	Park et al. (2012)
Hesperidin	Flavonoid	Hypertension (L-NAME-induced)	*In vivo:* Rats	↑NO ↓Oxidative stress, ↓Inflammation, ↓Fibrosis	Maneesai et al. (2018)
Hesperetin	Flavonoid	Hesperetin-induced vasodilation	*In vivo:* Spontaneously hypertensive rats	↑NO/sGC/cGMP pathway, ↑AC/cAMP/PKA pathway, ↑β2-adrenergic receptor activation, ↑KV and K_ATP_ channel opening ↓Intracellular calcium via VOCC and IP3R blockade	Tew et al. (2024)
Honokiol	Polyphenol	Palmitic acid-induced endothelial dysfunction	*In vitro:* HUVEC cells	↑NO, ↑eNOS activation ↓Inflammation	Qiu et al. (2015)
Icariside II	Flavonoid	STZ-induced diabetic cardiomyopathy	*In vivo:* Rats	↓Oxidative stress, ↓Apoptosis, ↓Akt, ↓NOS, ↓NF-κB	Yang et al. (2018)
Kaempferol	Flavonol	Hypertension induced using L-NAME (NO synthase inhibitor)	*In vivo*: Rat model of NO-deficient hypertension	↑CAT, ↑NO, ↑Left ventricular function ↓TNFR1 and TNFR2, ↓p-NF-κB, ↓TNF-α, ↓IL-6, ↓SOD,↓MDA, ↓TGF-β1, ↓PI3K, ↓Akt1, ↓Smad2/3, ↓Cardiac hypertrophy	Maneesai et al. (2023)
Kaempferitrin, kaempferol and *Bauhinia forficata* extract	Flavonol and extract	Endothelium-dependent vasorelaxation induced by L-NAME, ODQ	*In vitro*: Isolated aortic rings from NTR and SHR rats	↓Vasorelaxation via NO/sGC pathway	Cechinel-Zanchett et al. (2019)
Luteolin	Flavonoid	Endothelial dysfunction	*In vitro:* Rat venous endothelial cells	↑NO, ↑prostacyclin, ↓ROS	Assunção et al. (2021)
Morin	Flavonoid	Chemical-induced endothelial dysfunction	*In vivo:* Rats	↑NO, ↓NF-κB, ↓oxidative stress	James et al. (2023)
Myricetin	Flavonoid	ACE inhibition and NO production	*In vitro:* HUVEC	↑eNOS, ↑NO bioavailability ↓ROS, ↓ACE	Berköz et al. (2020)
Naringenin	Flavonoid	Vasorelaxation in diabetes	*In vivo:* Diabetic rats	↑Endothelium-dependent relaxation, ↓Contractile response	Fallahi et al. (2012)
Naringin	Flavonoid	Fructose-induced endothelial dysfunction	*In vivo:* Rats	↑NO, ↑eNOS	Malakul et al. (2018)
Orientin	Flavone	Cardiac remodeling	*In vivo:* Mice	↑Cardiac function, ↑eNOS/NO signaling ↓Fibrosis and apoptosis	Li et al. (2017a)
Osthole	Coumarin	ox-LDL-induced endothelial injury	*In vitro:* HUVEC	↑NO ↓ROS, ↓TNF-α, ↓IL-6	Wang et al. (2018)
α-pinene	Terpenoid	Vasorelaxation effects	*In vivo:* Murine blood vessels	↑NO production, ↑TRPA1 channels, ↑Endothelial-dependent vasorelaxation	Jin et al. (2023)
Procyanidin trimer	Polyphenol	Vascular disease	*In vitro:* RAECs	↑NO production, ↑Intracellular Ca^2+^ influx, Activation of multiple K^+^ channels	Byun (2012)
Protocatechuic acid	Phenolic compound	Hypertension	*In vivo:* SHR	↑PI3K-Akt-eNOS activation, ↑NO	Masodsai et al. (2019)
Puerarin	Isoflavone	Endothelial NO production	*In vitro*: EA.hy926 endothelial cells	↑eNOS phosphorylation, ↑NO ↓eNOS activation by AMPK and CaMKII inhibitors, ↓TNFα, ↓ICAM-1, ↓NF-κB	Hwang et al. (2011)
		Cholesterol excretion and vascular health	*In vivo:* Mice	↑CYP7A1, ↓Cholesterol, ↑eNOS, ↓Atherogenic index	Yan et al. (2006)
Quercetin	Flavonoid	K-carrageenan-induced rat-tail model	*In vivo*: Rat model of thrombosis and circulatory stasis	↑6-keto-PGF1α, ↑eNOS, ↓Thrombus, ↓TXB2, ↓ET-1	Gogoi et al. (2024)
		L-NAME-induced hypertensive rats	*In vivo*: Rats	↑Antioxidant activity, ↑NO, ↓Blood pressure, ↓NADPH oxidase	Calabró et al. (2018)
		Myocardial ischemia/reperfusion (I/R) models	*In vivo:* Myocardial I/R rat model	↑Mitochondrial K_ATP_ channel activation, ↑NO, ↑Ventricular pressure, ↓CK, ↓IL-1β, ↓TNF-α, ↓IL-6	Liu et al. (2021)
		HO-1 gene knockout mice	*In vivo:* ApoE (−/−) and wild-type mice	↑Endothelium-dependent relaxation, ↑NO, ↑ HO-1 ↓Atherosclerosis progression	Shen et al. (2013)
		Abdominal aortic aneurysm	*In vivo:* C57BL/6 mice	↓p47phox, ↓iNOS, ↓MMP, ↓JNK/AP-1 signaling activation	WANG et al. (2014)
Quercetin and morin	Flavonoids	STZ-induced	*In vitro*: Isolated aorta	↑NO production, ↓Vasodilation	Taguchi et al. (2020)
Quercetin and rutin	Flavonoid	Ang II-induced	*In vitro*: H9c2 myocardial cells	↓Oxidative stress, ↓Hypertrophy ↓Superoxide anion levels ↓JNK1/2 ↓ERK1/2, ↓p38 MAPK	Siti et al. (2021b)
Resveratrol	Polyphenol	ACh-induced	*In vivo*: Young (4 months) and old (26 months) mice *،*eNOS knockout mice	↑Acetylcholine ↑Dilation in isolated femoral arteries, ↑NO production, ↑Endothelium-derived hyperpolarizing factor pathways, ↑Vascular function	Räthel et al. (2007), Diaz et al. (2019)
		L-NAME and ODQ induction	*In vitro:* Cardiac fibroblasts	↑NO, ↑NOS, ↑cGMP, ↑NO signaling ↓mRNA expression of hypertrophic markers atrial and brain atrial natriuretic peptide ↓Angiotensin II-induced cardiac fibroblast proliferation	Wang et al. (2007)
		L-NAME and iberiotoxin induction	*In vitro:* Endothelium-dependent dilation	↑NO, ↑NOS activation	Nagaoka et al. (2007)
		Diabetes-related myocardial infarction rats	*In vivo:* DRMI rats	↑Total plasma insulin ↑eNOS, ↓VEGF ↑Lipid metabolism ↓Blood glucose, ↓Body weight, ↓Plasma triglyceride, ↓Oxidative stress	Yan et al. (2018)
		Vasorelaxant effects	*in vitro:* Endothelial cells	↑ Ca^+2^, ↑NO ↑Endothelial function	Elíes et al. (2011)
		Vascular effects	*In vitro:* BAECs and HUVECs	↑NO, Trigger the interaction between ERα, Cav-1, and c-SRC	Klinge et al. (2008)
		L-NAME-induction	*In vivo:* Rats T2DM	↑eNOS ↑Microvascular compromise by chronic inflammation ↑Vascular function	Wang et al. (2011)
		Cardiovascular effects	*In vitro:* HUVEC and EA.hy 926 cells	↑eNOS mRNA expression ↑eNOS protein, ↑NO ↑eNOS promoter activity	Wallerath et al. (2002)
		Vasoculture	*In vitro:* Cultured pulmonary	↑NOS	Hsieh et al. (1999)
		Endothelial colony-forming cells from a rat model of intrauterine growth restriction	*In vivo:* Rat model *In vitro:* ECFCs isolated from 6-month-old IUGR and control male rats	↑Sirtuin-1, ↑ECFC proliferation, ↑Capillary-like sprouting, ↑NO, ↑eNOS expression, ↓Oxidative stress, ↓Superoxide anion, ↑Cu/Zn SOD, ↓SIPS, ↓Beta-galactosidase, ↓p16ink4a	Guillot et al. (2023)
		Cardioprotection	*In vivo:* Rats *Ex vivo:* Isolated working heart	↑iNOS, ↑VEGF, ↑KDR, ↑eNOS, ↑NO-dependent signaling, ↑VEGF	Das et al. (2005)
		Endothelial function	*In vivo:* Wistar rats *Ex vivo Model:* Aortic rings	↑Acetylcholine, ↓Phenylephrine, ↓Angiotensin II, ↑Plasma nitrite/nitrate, ↓Superoxide production	Soylemez et al. (2009)
		L-NAME induction	*In vivo:* Male Wistar rats	↑GSH/GSSG ratio, ↓Sympathetic modulation to vessels, ↑BRS, ↓LF and HF components of PI variability	Dillenburg et al. (2013)
Rhynchophylline	Alkaloid	Diabetes, STZ	*Ex vivo Model:* Aortic rings *In vivo:* Rats	Microvascular relaxation activity Improve endothelial dysfunction ↑eNOS	Guo et al. (2017)
Rutaecarpine	Alkaloid	NO synthesis enhancement	*In vitro:* Human endothelial cells	↑eNOS phosphorylation, ↑NO via TRPV1 activation ↑CaMKII, ↑CaMKKβ/AMPK signaling ↓ICAM-1, ↓VCAM-1	Lee et al. (2021)
Rutin	Flavonoid	HUVEC cell experiments	*In vitro*: HUVEC cells	↑NO, ↑bFGF	Ugusman et al. (2014)
Scutellarin	Flavonoid	Hypocholesterolemic and atheroscleroprotective effects	*In vivo*: Rats on an atherogenic diet	↑NO, ↑Endothelium-dependent vasorelaxation ↓Serum total cholesterol levels, ↓Atherogenic index	Li et al. (2009)
Secoisolariciresinol Diglucoside	Lignan	Anti-inflammatory effects	*In vitro:* LPS-stimulated HUVEC	↓IL-1β, ↓IL-6, ↓TNF-α, ↑NO, ↓NF-κB	Zhang et al. (2020b)
Silibinin	Flavonoid	Vascular function modulation	*In vivo:* Ovariectomized rats	↑Endothelial integrity ↑eNOS, ↓Ang-II, ↓ET-1	Maleki et al. (2022)
Tanshinone IIA	Diterpenoid	Ang II-induced endothelial dysfunction	*In vitro:* Human endothelial cells	↑NO production, ↓ROS, ↓ERK phosphorylation	Chan et al. (2011)
Tilianin	Flavonoid	Myocardial ischemia/reperfusion injury	*In vivo:* Rat	↑Endothelial function, ↑Na/K ATPase and Ca ATPase activities, ↓Calcium overload, ↓Apoptosis, ↓NLRP3 inflammasome activation	Guo et al. (2015)
*trans-*dehydrocrotonin (*trans*-DCTN)	Diterpene	Hemodynamic parameter analysis	*In vivo:* Rats	↑NO ↓Blood pressure, ↓Heart rate, ↑Vasorelaxation	Silva et al. (2005)
Whitanolide A	Steroidal lactone	Endothelial dysfunction	*In vitro:* EA.hy926 cells, rat aortic rings	↑NO release, ↑eNOS activation	Pathak et al. (2021)
Several phenolic compounds	Phenolic compounds	Relaxing activity	*In vitro:* Porcine coronary rings	↑NO, ↑Vasorelaxing activity	Taubert et al. (2002)

6-keto-PGF1α, 6-Keto-Prostaglandin F1 alpha; AC/cAMP/PKA, Adenylate Cyclase/Cyclic AMP/Protein Kinase A; ACE, Angiotensin-Converting Enzyme; ACh, Acetylcholine; AhR-RGE, Aryl Hydrocarbon Receptor-Regulated Gene Enhancement; Ahr-TN, Aryl Hydrocarbon Receptor-Translocated; AI, atherogenic index; Akt, Protein Kinase B ALDH, aldehyde dehydrogenase; AMPK, AMP-Activated Protein Kinase; Ang II, Angiotensin II; Ang II-CFP, Angiotensin II; Central Fusion Point; AP-1, Activator Protein 1; ARNT, aryl hydrocarbon receptor nuclear translocator; AS, atherosclerosis; ATPases, Adenosine Triphosphatases; Bcl-2/Bax, B-cell Lymphoma 2/Bcl-2-associated X Protein; bFGF, basic fibroblast growth factor; BMI, body mass index; BP, blood pressure; BRS, baroreflex sensitivity; CaMKII, Calcium/Calmodulin-Dependent Protein Kinase II; CaMKKβ, Calcium/Calmodulin-Dependent Protein Kinase Kinase Beta CAT, catalase; CD39, Cluster of Differentiation 39; CD73, Cluster of Differentiation 73; CDR, cognitive decline rate; cGMP, cyclic guanosine monophosphate; CK, creatine kinase; CLS, Capillary-like Structures; cNOS, constitutive nitric oxide synthase; CPK, creatine phosphokinase; CS, Cholesterol SulfatecTnI, Cardiac Troponin I; CVP, central venous pressure; CYP7A1, Cholesterol 7 Alpha-Hydroxylase; ECFC, Endothelial Colony-Forming Cells; EDHF, Endothelium-Derived Hyperpolarizing Factor; EDR, Endothelium-Dependent Relaxation; EDV, end diastolic volume; eNO, endothelial nitric oxide; eNOS, endothelial nitric oxide synthase; ERK1/2, Extracellular Signal-Regulated Kinases 1/2,ET-1, Endothelin-1; GC, guanylate cyclase; GSH/GSSG, Reduced Glutathione/Glutathione Disulfide Ratio; GSH-Px, Glutathione Peroxidase; GSK-3β, Glycogen Synthase Kinase 3 Beta; H_2_O_2_, hydrogen peroxide; Hcy-ED, Homocysteine-Endothelial Dysfunction; HDL, High-Density Lipoprotein; HDL-C, High-Density Lipoprotein Cholesterol; HO-1, Heme Oxygenase 1; ICAM-1, Intercellular Adhesion Molecule 1; IL-1β, Interleukin 1 Beta; IL-6, Interleukin 6; iNOS, inducible nitric oxide synthase; IP3R, Inositol 1,4,5-Trisphosphate Receptor,JNK, c-Jun N-terminal kinase,JNK1/2, c-Jun N-terminal kinases 1/2; K_ATP_, ATP-Sensitive Potassium Channel; KCM, potassium channel modulation; KDR, kinase insert domain receptor; K-Ras, Kirsten Rat Sarcoma Viral Oncogene Homolog; KV; and K_ATP_, Voltage-Gated Potassium and ATP-Sensitive Potassium Channels; LDH, lactate dehydrogenase; LDL, Low-Density Lipoprotein; LDL-C, Low-Density Lipoprotein Cholesterol; LF and HF, low frequency and high frequency components of power index; LPO, lipid peroxidation; MAP, mean arterial pressure; MAPK, Mitogen-Activated Protein Kinase; MDA, Malondialdehyde,MF, myocardial fibrosis; mitoK_ATP_, Mitochondrial ATP-Sensitive Potassium Channel; MMP, matrix metalloproteinase; mRNA, messenger ribonucleic acid; NADPH, nicotinamide adenine dinucleotide phosphate; NLRP3, NLR; Family Pyrin Domain Containing 3; NO, Nitric Oxide,NOS2 and NOS3, Nitric Oxide Synthase 2 and 3; NOX4, NADPH; Oxidase 4; NQO1, NAD(P)H Quinone Dehydrogenase 1; NRC, nuclear regulatory commission; Nrf2, Nuclear Factor Erythroid 2–Related Factor 2; OS, oxidative stress; p38 MAPK, p38 Mitogen-Activated Protein Kinases; p47phox, Phox Subunit of NADPH; oxidase; PE, phenylephrine; PGI, prostacyclin; PI3K, Phosphoinositide 3-Kinase,p-NF-κB, Phosphorylated Nuclear Factor kappa B,PTEN, phosphatase and tensin homolog; RAS, Renin-Angiotensin System; ROS, reactive oxygen species; RS, renal stenosis; SAL, salinity; sGC, soluble guanylate cyclase; SIPS, Stress-Induced Premature Senescence; Smad2/3, SMAD; Family Member 2/3; SOD, superoxide dismutase; STCL, short term cellular leukemia; TG, Triglycerides,TGF-β1, Transforming Growth Factor Beta 1; TNFR, Tumor Necrosis Factor Receptor,TNF-α, tumor necrosis factor alpha; TPE, total protein excretion; TRPA1, Transient Receptor Potential Ankyrin 1; TXB2, Thromboxane B2; VCAM-1, Vascular Cell Adhesion Molecule 1; VD, vascular diameter; VEGF, vascular endothelial growth factor; VOCC, Voltage-Operated Calcium Channels; VP, venous pressure; VR, vascular resistance; β2-AR, Beta-2; adrenergic receptor; β-Gal, Beta-Galactosidase.

Treatment with the flavonoid monomer 17-methoxyl-7-hydroxy-benzene-furanchalcone (MHBFC) for 6 weeks in male rats with cardiac remodeling caused by abdominal aortic stenosis led to significant improvements, effectively reversing the harmful effects induced by L-NAME. This included reductions in myocardial cell cross-sectional area and fibrosis, along with increased eNOS activity and decreased apoptosis in myocardial microvascular endothelial cells. The results suggest that MHBFC enhances eNOS protein phosphorylation via the PI3K/Akt pathways, thereby increasing NO production and providing protection against myocardial injury (Ye et al., 2019).

3-Hydroxyphenylacetic acid is a monocarboxylic acid and metabolite derived from dietary phenols and flavonoids found in foods like teas and fruits (Liu et al., 2022). Research indicated that this metabolite could lower arterial blood pressure in a dose-dependent manner when administered intravenously to spontaneously hypertensive rats, without significantly affecting heart rate. *Ex vivo* studies showed that 3-hydroxyphenylacetic acid induces relaxation of precontracted porcine coronary artery segments, a process that depends on endothelial integrity. Inhibition of eNOS reduces this relaxing effect, suggesting that this metabolite lowers blood pressure primarily by promoting NO release from endothelial cells, thereby facilitating vascular relaxation (Dias et al., 2022).

Klider et al. highlighted that apigenin (5,7,4′-trihydroxyflavone), a flavonoid with cardiovascular benefits, causes dose-dependent vasodilation in SHR rats. They proposed that this effect depends on endothelial function and is mediated by NO. Additionally, potassium channel blockers further reduce vasodilation, particularly by inhibiting calcium-activated potassium channels. Notably, simultaneous inhibition of NO and blockade of potassium channels completely abolishes the effects of apigenin (Klider et al., 2024a).

Astragaloside IV, a cycloartane-type triterpene glycoside derived from *Astragalus mongholicus* Bunge [Fabaceae], has shown protective effects against this dysfunction by promoting vasodilation via the PI3K/Akt/eNOS signaling pathway. Astragaloside IV increased NO levels in a concentration-dependent manner and enhanced the relaxation of isolated rat aortic rings. The mechanism involves phosphorylation of Akt at Ser473 and dephosphorylation of eNOS at Thr495, thereby upregulating eNOS expression (Lin et al., 2018).

Baicalein (5,6,7-trihydroxyflavone), a natural flavonoid, was administered at the onset of reperfusion and demonstrated a significant reduction in cell death in a concentration-dependent manner. The underlying mechanism included effective ROS scavenging and enhanced NO production, particularly at the 100 μM dose. Furthermore, co-treatment with the NO synthase inhibitor L-NAME partially diminished the cytoprotective effects of baicalein, suggesting that NO plays a crucial role in mediating its protective action (Chang et al., 2019). Li et al. reported that baicalein exhibits significant cardioprotective effects against ischemia-reperfusion injury. Their study showed that brief treatment with baicalein induces controlled mitochondrial ROS production, which activates protective signaling pathways, notably Akt and eNOS phosphorylation, leading to increased NO production. Additionally, baicalein promoted metabolic recovery by upregulating pyruvate dehydrogenase activity, thereby increasing ATP production during reperfusion (Li et al., 2022). In another study using a chick cardiomyocyte model of ischemia/reperfusion injury, three baicalein treatment strategies were assessed for their effects on ROS scavenging, NO production, and cell viability. The findings revealed that preventive treatment with baicalein provided the most significant cardioprotection, markedly reducing cell death and ROS generation while enhancing NO production and Akt phosphorylation. Additionally, the study highlighted the involvement of the phosphatase and tensin homolog (PTEN)/Akt/NO signaling pathway in the protective effects (Li J. et al., 2017). Tan et al. linked the protective effects of baicalein (30 mg/kg) against injuries caused by acute myocardial infarction to activation of eNOS signaling and a reduction in oxidative stress (Tan et al., 2014). In a mouse study, cardiac remodeling was induced by left coronary artery ligation.

Berberine is a bioactive compound belonging to the isoquinoline alkaloid class (Neag et al., 2018). Berberine exerted a protective effect against hyperglycemia-induced endothelial dysfunction in a dose-dependent manner. It enhances phosphorylation of eNOS at Ser1177, thereby increasing NO production by promoting eNOS association with heat shock protein 90 (HSP90). Additionally, berberine reduces ROS generation and cellular apoptosis while inhibiting NF-κB activation and adhesion molecule expression, thereby preventing monocyte attachment to endothelial cells. In mouse aortic rings, it induces endothelium-dependent vasodilatation, thereby counteracting high-glucose-induced dysfunction. These effects are mediated via the activation of the AMPK signaling pathway (Wang C. et al., 2009).

Boldine is a primary aporphine alkaloid derived from the leaves and bark of *Peumus boldus* Molina [Monimiaceae] (Fuentes-Barros et al., 2023). Recent studies using perfused rat kidneys have shown that boldine induces vasodilation of renal arteries in a dose-dependent, endothelium-dependent manner, with effective doses ranging from 30 to 300 nmol. Inhibition of NOS with L-NAME completely negated boldine’s vasodilatory effects, indicating that NO production is essential for this action. Furthermore, the involvement of small-conductance calcium-activated potassium channels was confirmed, as the selective blocker apamin prevented the vasodilatory response elicited by boldine (De Souza et al., 2022).

The bornesitol (methyl ether of D-myo-inositol) is a cyclitol identified as the primary active compound in the leaves of *Hancornia speciosa* Gomes [Apocynaceae]. In a study involving rats, administering varying doses of bornesitol intravenously significantly lowered systolic and diastolic blood pressure, increased plasma nitrite levels, and reduced ACE activity. The mechanism of action involved enhanced NO production, which facilitated endothelium-dependent vasodilation, a process that was inhibited by NOS blockade. Additionally, while inhibiting the calcium-calmodulin complex diminished the vasodilatory effect, blocking the PI3K/Akt pathway did not affect it (Moreira et al., 2019).

Caffeine and caffeic acid, administered individually or in combination at doses of 5 and 25 mg/kg over 14 days, significantly decreased the activities of angiotensin converting enzyme (ACE) and arginase, while also lowering MDA levels and increasing NO levels. Reducing ACE activity lowers angiotensin II levels, thereby facilitating vasodilation (Oboh et al., 2021).

Evaluation of the protective effects of chlorogenic acid (caffeoyl quinic acid) found in coffee on endothelial function in aortic rings isolated from C57BL mice and human aortic endothelial cells (HAECs) under oxidative stress revealed increased NO production and HO-1 expression (Jiang et al., 2016).

Cinnamic acid and cinnamaldehyde are two important compounds derived from the cinnamon plant. In a rat model, cinnamic aldehyde and cinnamic acid demonstrated protective effects against ST-elevation induced by acute myocardial ischemia. These compounds significantly reduced serum levels of creatine kinase, lactate dehydrogenase (LDH), tumour necrosis factor alpha (TNF-α), and interleukin-6 (IL-6). Additionally, they enhanced serum NO activity. In myocardial tissue, they increased superoxide dismutase (SOD) activity while decreasing malondialdehyde (MDA) content, indicating a potential antioxidant effect (Song et al., 2013).

In a study involving rats with induced hypertension, curcumin, a key component of turmeric (Tarlan et al., 2025), significantly improved hemodynamic performance, reducing blood pressure and enhancing blood flow. Furthermore, curcumin was found to promote better endothelium-dependent vasorelaxation while alleviating oxidative stress and vascular remodeling associated with hypertension. The antihypertensive effects of curcumin are attributed to mechanisms such as increased NO bioavailability and decreased markers of oxidative stress, including reduced superoxide production (Boonla et al., 2014). Ramaswami et al. suggested that curcumin mitigates endothelial dysfunction by enhancing NO bioavailability and reducing oxidative stress (Ramaswami et al., 2004).

A study investigated the impact of delphinidin, an anthocyanin found in red wine, on apoptosis in bovine aortic endothelial cells. Delphinidin significantly reduced apoptosis triggered by actinomycin D and 7-beta-hydroxycholesterol. This protective effect was negated by NOS, GC, and MAPK inhibitors, indicating that delphinidin’s action involves these signaling pathways (Martin et al., 2003).

A study on ovariectomized spontaneously hypertensive rats demonstrated that ellagic acid, a natural polyphenol, improved endothelial-dependent vasodilation similarly to 17-β-estradiol, with effects negated by L-NAME. Ellagic acid increased vascular NO release by enhancing eNOS phosphorylation and total levels. Furthermore, ellagic acid reduced superoxide anion levels and elevated the activity of antioxidant enzymes, including SOD and catalase (CAT) (da Silva et al., 2022).

The *in silico* and *in vitro* findings reveal that (−)-epicatechin interacts with arginase, thereby reducing its activity. *In vivo* studies demonstrated that a 10-day pretreatment with 1 mg/kg of (−)-epicatechin reduces arginase expression in ischemic myocardium, while simultaneously increasing NOS expression and phosphorylation levels (Ortiz-Vilchis et al., 2018). (−)-epicatechin administration before reperfusion significantly reduced infarct size. Mechanistically, it improved mitochondrial function by reducing respiratory inhibition and mitochondrial calcium overload, which correlated with increased ATP levels in tissues. It stimulated maximal respiration rates in neonatal rat ventricular myocytes, an effect blocked by specific inhibitors (L-NAME, ODQ, or sGC), indicating its action on mitochondrial pathways (Yamazaki et al., 2014).

MacRae et al. investigated the effects of epicatechin, a flavonoid known for its cardioprotective properties, on cardiovascular function using isolated rat arteries and cardiac electrophysiology. The study found that epicatechin induced significant vasodilation in pre-contracted vessels at doses ranging from 10^–9^ to 10^–4^ M. This vasodilatory effect was diminished by antagonists such as naloxone, L-NAME, and calcium channel blockers, indicating the involvement of opioid receptors, NO, and calcium channels. Furthermore, epicatechin enhanced cardiac electrophysiology by lowering action potential parameters, an effect also diminished by naloxone (MacRae et al., 2019).

In vascular endothelial cells, epigallocatechin gallate (EGCG) has been shown to enhance NO production via signaling pathways that activate Fyn/PI3K/Akt/eNOS, resulting in vasodilation (Kim et al., 2007). Subsequent research revealed that this polyphenol also reduced ET-1 expression and secretion by regulating forkhead box O1 (FOXO1) through Akt and AMPK pathways, underscoring its potential cardiovascular benefits (Reiter et al., 2010). Acute and chronic administration of EGCG was associated with dose-dependent vasodilation, which was inhibited by NO synthase and PI3k inhibitors. It resulted in decreased systolic blood pressure and improved insulin sensitivity in SHR (Potenza et al., 2007). It was reported that combining EGCG with low-dose vardenafil significantly enhances cell proliferation and NO production in H9C2 cardiomyocytes while also protecting against oxidative damage (Wang et al., 2020). A rat study found that EGCG exerted direct NOS-dependent vasodilatory effects in skeletal muscle, without immediate changes in muscle glucose uptake or amplification of insulin’s vascular and metabolic effects in healthy rats (Ng et al., 2017).

In exploring alternatives to conventional estrogen replacement therapy to lower cardiovascular disease risk in postmenopausal women, research on equol—a metabolite of the soy isoflavone daidzein—found that it has superior antioxidant properties compared to its parent compounds, genistein and daidzein. Equol has been shown to effectively inhibit the oxidation of LDL cholesterol and its alteration by macrophages, indicating a potential mechanism by which increased NO levels prevent LDL modification. The antioxidant effects of equol appear to stem from its ability to reduce superoxide radical production, thereby increasing free NO levels, which play a vital role in supporting vascular health (Hwang et al., 2003).

Ginsenoside Rd, a protopanaxadiol (ppd)-type saponin of *Panax notoginseng* (Burkill) F.H.Chen [Araliaceae] was studied for its protective effects against nicotine-induced damage to vascular endothelial cells. In HUVECs, ginsenoside Rd countered nicotine’s damaging effects by increasing NO and eNOS production, decreasing angiotensin II levels, and reducing the expression of apoptosis-related proteins. In nicotine-treated rats, ginsenoside Rd increased serum NO and angiotensin II levels, protected aortic endothelial cells, and reduced monocyte adhesion, platelet aggregation, and vasoconstriction. The protective mechanisms involved maintaining normal NO signaling and inhibiting inflammatory pathways associated with TLR4 and NF-κB (Zhang B. et al., 2020). The cardioprotective effects of ginsenoside Rg3 were also linked to Akt/eNOS signaling and the Bcl-2/Bax pathway (Wang et al., 2015).

Gomisin J, a lignan derived from *Schisandra chinensis* (Turcz.) Baill. [Schisandraceae] elicited concentration-dependent vasorelaxation in the rat thoracic aorta, with pronounced effects observed in endothelium-intact samples that L-NAME significantly diminished. Additionally, Gomisin J promoted NO production and facilitated eNOS phosphorylation in both rat and human endothelial cells via calcium- and PI3K/Akt-dependent mechanisms (Park et al., 2012).

Hesperidin, a flavanone glycoside found in citrus fruits (Hajialyani et al., 2019), was investigated for its cardioprotective effects in rats with hypertension induced by L-NAME (40 mg/kg). Treatment with hesperidin at 15 and 30 mg/kg, in combination with captopril (2.5 mg/kg), significantly mitigated hypertension and reduced cardiac and vascular remodeling, as evidenced by decreased wall thickness, cross-sectional area, and fibrosis in the left ventricle and the aorta. These protective effects were associated with lower oxidative stress markers, reduced levels of TNF-α and TGF-β1, and increased plasma NO metabolites. Hesperidin also inhibited the upregulation of TNF-R1, matrix metallopeptidase (MMP)-2, and MMP-9 (Maneesai et al., 2018). Hesperedin reduced monocyte ICAM-1 and VCAM-1 expression in response to TNF-α treatment, suggesting its potential to mitigate inflammation and improve endothelial function by enhancing NO signaling (Rizza et al., 2011).

*In vitro* studies demonstrated that hesperetin, the aglycone of hesperidin, stimulated the phosphorylation of key proteins (Src, Akt, AMP kinase (AMPK), and eNOS) in bovine aortic endothelial cells, leading to enhanced NO production. Another study found that hesperetin promoted vasodilation by increasing NO levels and cyclic nucleotide levels, primarily through the NO/sGC/cGMP signaling pathway. Additionally, it activated the adenylyl cyclase (AC)/cAMP/PKA pathway via PGI2 and stimulated the β2-adrenergic receptor. Hesperetin also functioned as an opener of voltage-gated potassium channels and K_ATP_, leading to reduced intracellular calcium levels in vascular smooth muscle by blocking voltage-operated calcium channels and IP3 receptors. *In vivo* studies have demonstrated that the oral administration of hesperetin significantly lowered blood pressure in spontaneously hypertensive rats over 21 days (Tew et al., 2024).

Honokiol, a polyphenolic derived from *Magnolia officinalis* Rehder and E.H.Wilson [Magnoliaceae], significantly suppressed pentraxin 3 overexpression in palmitic acid-induced HUVECs by inhibiting the phosphorylation of IκB and NF-κB. Furthermore, honokiol reduced endothelial cell injury and apoptosis by modulating iNOS and eNOS expression and NO production. Honokiol showed anti-inflammatory effects in HUVECs by significantly inhibiting the production of IL-6, IL-8, and monocyte chemoattractant protein-1 (MCP-1) (Qiu et al., 2015).

A study focused on the effects of icariside II, a flavonoid derived from *Epimedium brevicornum* Maxim. [Berberidaceae], in diabetic cardiomyopathy in rats induced by STZ, found that treatment with icariside II led to significant improvements in body weight, heart-to-body weight ratio, and fasting blood glucose levels. Furthermore, it effectively reduced serum levels of creatine kinase and lactate dehydrogenase, while also mitigating cardiac oxidative stress, inflammation, and apoptosis in the diabetic model. Its mechanism appears to involve the activation of the Akt/NOS/NF-κB signaling pathway (Yang et al., 2018).

Kaempferol (3,4′,5,7-tetrahydroxyflavone), a natural flavonol, exhibited significant cardioprotective effects in rats suffering from NO deficiency induced by L-N^G^-Nitro arginine methyl ester (L-NAME). It effectively reduced hypertension and improved left ventricular function and hypertrophy. At the molecular level, kaempferol inhibited the overexpression of TNF-α receptors (TNFR1 and TNFR2), phosphoinositide 3-kinases (PI3Ks), protein kinase B (AKT), and Smad2/3 in cardiac tissue, as well as p-nuclear factor kappa B (NF-κB) and transforming growth factor beta 1 (TGF-β1) in vascular tissue. Additionally, it restored superoxide (O_2_^•–^) formation, MDA levels, CAT activity, plasma NO metabolites, TNF-α, and IL-6. These findings indicate that kaempferol possesses cardioprotective, antihypertensive, anti-inflammatory, and antioxidant properties in NO-dependent hypertensive rats (Maneesai et al., 2023). In a study, the vascular effects of the extract from the leaves of *Bauhinia forficata* Link [Fabaceae] and its primary compounds, kaempferol and kaempferitrin (kaempferol 3,7-dirhamnoside), were investigated using aortic rings from normal and spontaneously hypertensive (SHR) rats. The ethyl acetate and butanol fractions demonstrated both endothelium-dependent and independent vasorelaxant properties. The vasorelaxation induced by this fraction was inhibited by L-NAME and 1H-(1,2,4)oxadiazolo (4,3-a)quinoxalin-1-one (ODQ), suggesting the involvement of the NO/sGC pathway. Additionally, their activity was significantly influenced by certain potassium channel blockers (Cechinel-Zanchett et al., 2019).

Luteolin (5,7,3′,4′-tetrahydroxyflavone), a flavonoid with antioxidant properties, was studied for its effects on rat venous endothelial cells at concentrations of 10, 20, and 50 μmol/L. Luteolin significantly increased NO levels while decreasing ROS generation. This reduction in ROS was correlated with a significant decrease in 3-NT residues and an increase in prostacyclin (PGI2) release (Assunção et al., 2021).

Morin (2′,3,4′,5,7-pentahydroxyflavone) was evaluated for its effects on cardiac endothelial function in rats exposed to a mixture of bisphenol S and diethyl phthalate. Exposure to this mixture significantly increased oxidative stress markers and inflammatory mediators, while decreasing NO and antioxidant enzyme activities. Notably, morin treatment reversed these adverse effects in a dose-dependent manner, enhancing NO levels and antioxidant enzyme activities, and reducing markers of inflammation and apoptosis. The underlying mechanism appears to involve modulation of oxidative stress and inflammatory pathways, particularly by inhibiting NF-kB signaling (James et al., 2023).

Myricetin belongs to the flavonoid class of compounds. The investigation into myricetin’s effects on HUVEC involved several key parameters. Myricetin significantly inhibited angiotensin-converting enzyme (ACE) activity, a critical regulator of blood pressure. Additionally, treatment with myricetin increased NO production by reducing ROS levels and enhancing eNOS activation. The activation of eNOS was associated with an increase in cellular calcium concentration, an essential requirement for eNOS function (Berköz et al., 2020).

Naringenin (4′, 5, 7-trihydroxyflavonone) is a flavanone found in citrus fruits. In diabetic rats treated with naringenin (10 mg/kg for 5 weeks), the maximum contractile responses of endothelium-intact aortic rings to potassium chloride and phenylephrine were significantly reduced. Conversely, endothelium-dependent relaxation in response to acetylcholine (ACh) was markedly enhanced in these rats. Pretreatment of the aortic rings with L-NAME substantially diminished the observed relaxation response (Fallahi et al., 2012).

Naringin is a natural flavanone glycoside derived from naringenin. Malakul et al. investigated the effects of naringin, a citrus flavonoid, on fructose-induced endothelial dysfunction in rats. Following 12 weeks of a 10% fructose diet, rats that received naringin treatment (100 mg/kg) for 4 weeks, starting from week 8, showed significant restoration of metabolic parameters and improved endothelial function. Naringin effectively reversed fructose-induced elevations in blood glucose, total cholesterol, and low-density lipoprotein levels. Additionally, it enhanced NO bioavailability by increasing eNOS expression and phosphorylation while simultaneously reducing nitrotyrosine (NT) levels (Malakul et al., 2018).

Orientin, a C-glycosyl flavonoid derived from *Persicaria orientalis* (L.) Spach syn. *Polygonum orientale* L. [Polygonaceae] reduced mortality and improved cardiac function after 4 weeks. Notably, orientin reduced fibrosis, inflammatory responses, and cardiomyocyte apoptosis. Furthermore, it enhanced cell viability in hypoxic neonatal rat cardiomyocytes and mitigated oxidative stress in heart tissue. The cardioprotective effects of orientin were linked to activation of the eNOS/NO signaling pathway (Li F. et al., 2017).

Osthole, a naturally derived coumarin, was found to alleviate ox-LDL-induced endothelial injury in HUVECs. It did not affect cell viability but significantly reduced ox-LDL-induced cytotoxicity by decreasing the release of inflammatory cytokines, including TNF-α, IL-1β, and IL-6. Osthole also reversed the increase in ROS and MDA levels while restoring SOD activity. Additionally, it decreased mRNA expression and secretion of the adhesion molecules ICAM-1 and VCAM-1, thereby improving endothelial function. Significantly, osthole enhanced NO production and phosphorylated eNOS in ox-LDL-treated HUVECs. Furthermore, osthole inhibited the activation of the TGF-β1/Smad signaling pathway, which is implicated in endothelial dysfunction (Wang et al., 2018).

α-Pinene, a volatile organic compound derived from plants, exhibits notable cardiovascular effects. When exposed to α-pinene (1 ppm for 6 h), metabolites such as myrtenol and verbenol are produced, leading to significant vasorelaxation in murine blood vessels. At a concentration of 0.3 mM, these metabolites effectively reduce vascular tension, whereas 1 mM is toxic to the vasculature. Vasorelaxation is facilitated by endothelial activation and enhanced NO production. Furthermore, α-pinene activated the transient receptor potential ankyrin 1 (TRPA1) channel, amplifying its vascular effects and suggesting its potential to improve cardiovascular health through increased NO release (Jin et al., 2023).

Procyanidin C1 is a notable B-type proanthocyanidin, specifically an epicatechin trimer, primarily found in sources such as grapes, unripe apples, and cinnamon. Byun, in a study examining the effects of procyanidin C1 on rat aortic endothelial cells (RAECs), found that procyanidin C1 induced hyperpolarization dependent on potassium channels, enhanced intracellular calcium influx, and boosted NO production. These effects were reduced by the inhibitor L-NAME and tetraethylammonium chloride (Byun, 2012).

In a study conducted on SHR, the administration of protocatechuic acid, a phenolic compound, enhanced insulin- and insulin-like growth factor-1 (IGF-1)-induced vasorelaxation, indicating improved endothelial responsiveness. The mechanism underlying this effect involves activation of the PI3K pathway, which increases NO production via the eNOS enzyme. Notably, vasorelaxation was abolished entirely when PI3K or NO synthase inhibitors were applied, confirming the critical role of this pathway (Masodsai et al., 2019).

Puerarin, an isoflavone derivative derived from *Pueraria montana* var. *lobata* (Willd.) Maesen and S.M. Almeida ex Sanjappa and Predeep [Fabaceae] exhibits cardioprotective properties by activating endothelial nitric oxide synthase (eNOS) in EA. hy926 endothelial cells. It facilitated phosphorylation of eNOS at Ser1177, leading to increased NO production, primarily through estrogen receptor-mediated PI3K/Akt signaling. The activation of eNOS by puerarin was obstructed by inhibitors of AMP-activated protein kinase (AMPK) and calcium/calmodulin-dependent protein kinase II (CaMKII). Furthermore, puerarin diminished TNFα-induced monocyte adhesion to endothelial cells and reduced the expression of ICAM-1 and NF-κB, thereby decreasing inflammation (Hwang et al., 2011). Another study found that oral administration of puerarin (300 mg/kg) might increase cholesterol excretion and support vascular health. In this study, puerarin significantly reduced total cholesterol levels and atherogenic index in mice. Additionally, puerarin increased hepatic 7-alpha-hydroxylase (CYP7A1) expression and improved endothelial function by reducing eNOS abnormalities (Yan et al., 2006).

Gogoi et al.’s study indicated that quercetin derived from *Solanum lasiocarpum* Dunal [Solanaceae] (syn. *Solanum indicum*) exhibits both anticoagulant and antithrombotic effects *in vivo*. Quercetin significantly inhibited thrombus formation triggered by k-carrageenan in the rat-tail model. Additionally, in a model of acute circulatory stasis, quercetin was found to lower the levels of thromboxane B2 (TXB2) and ET-1, while simultaneously enhancing the levels of eNOS and 6-keto prostaglandin F1α (6-keto-PGF1α) (Gogoi et al., 2024). Quercetin administration effectively prevented the rise in blood pressure and the activation of nicotinamide adenine dinucleotide phosphate (NADPH) oxidase, but did not alter the decrease in NOS activity induced by L-NAME. Additionally, quercetin positively influenced oxidative stress markers, thereby improving NO bioavailability by regulating superoxide production (Calabró et al., 2018). In their study, Liu et al. proposed that the protective effects of quercetin in myocardial ischemia/reperfusion models are associated with the activation of mitochondrial K_ATP_ channels and NO pathways. Quercetin notably improved ventricular pressure and reduced creatine kinase release. Furthermore, it lowered the levels of inflammatory cytokines, including IL-1β, TNF-α, and IL-6 (Liu et al., 2021). Quercetin supplementation (0.05% w/w) significantly reduced atherosclerosis in ApoE (−/−) mice and improved endothelium-dependent relaxation in wild-type mice. Mechanistic analysis revealed enhancements in NO bioavailability and increased heme oxygenase-1 (HO-1) expression, contributing to its protective effects against oxidant-induced endothelial dysfunction. However, quercetin did not mitigate endothelial dysfunction in arteries from HO-1 gene knockout mice, indicating a critical relationship between HO-1 induction and quercetin’s efficacy (Shen et al., 2013). Wang et al. investigated quercetin’s role in inhibiting abdominal aortic aneurysm formation in male C57BL/6 mice, revealing that quercetin treatment reduced the expression of key proteins involved in oxidative stress and inflammation, such as p47phox and iNOS, and inhibited matrix metalloproteinase activation. These quercetin’s protective effects may be mediated through c-Jun N-terminal kinases (JNK)/activator protein-1 signaling pathways (Wang et al., 2014).

Quercetin and β-naphthoflavone activate the aryl hydrocarbon receptor (Ahr), which plays a vital role in cellular signaling pathways. In cellular models of oxygen-glucose deprivation/reoxygenation, both compounds were shown to enhance antioxidant capacity, thereby reducing ROS overproduction and cell death rates. Mechanistically, they promote the translocation of Ahr into the nucleus, where it competes with hypoxia-inducible factor (HIF)-1α for binding to the Ahr nuclear translocator (ARNT), thus inhibiting HIF-1α-mediated cardioprotective effects like NO and vascular endothelial growth factor (VEGF) production (Li W. et al., 2017). However, clinical studies indicate that acute administration of quercetin does not produce significant changes in NO-mediated endothelial relaxation or blood pressure regulation in healthy adults (Bondonno et al., 2016).

A comparative study examined the effects of quercetin and morin on aortas isolated from control and streptozotocin (STZ)-induced diabetic mice. Both quercetin and morin (10^–6^ M) were effective vasodilators, increasing NO production by phosphorylating Akt and eNOS. The vasodilatory effects of both flavonoids were inhibited by an Akt inhibitor, highlighting the significance of the Akt pathway. However, the effects of quercetin were further diminished by PI3K and AMPK inhibitors, suggesting a more intricate mechanism involving these pathways. In contrast, morin cells primarily activated the Akt pathway (Taguchi et al., 2020).

In a study, H9c2 cardiomyocytes were treated with angiotensin II to induce hypertrophy, followed by exposure to quercetin (331 μM) or rutin (50 μM) for 24 h. Both flavonoids effectively reversed the oxidative stress and hypertrophic effects induced by angiotensin II, as evidenced by reduced cell surface area and superoxide anion levels. Rutin specifically downregulated phosphorylated JNK1/2, while quercetin demonstrated a more substantial inhibitory effect on multiple mitogen-activated protein kinase (MAPK) pathway proteins, including extracellular signal-regulated kinases (ERKs) and p38 (Siti et al., 2021b). Challa et al. highlighted the role of NO in the cardioprotective mechanisms of these two flavonoids against myocardial ischemia-reperfusion injury (Challa et al., 2011).

Researchers produced polyphenol extracts from various red wines to assess their impact on eNOS promoter activity using a luciferase reporter gene assay. Results indicated significant variability in extract activity by grape origin and cultivar, although no specific correlation emerged when averaged across samples. Notably, resveratrol was identified as a key component that enhances eNOS activity, albeit at concentrations higher than typically found in red wine (Räthel et al., 2007). A study examined the acute effects of *trans*-resveratrol on ACh- induced dilation in isolated femoral arteries from both young (4 months) and old (26 months) mice. Resveratrol enhanced ACh-induced dilation but did not affect flow-mediated dilation. It increased NO production in endothelial cells in response to ACh while reducing it during flow conditions. In eNOS knockout mice, resveratrol still potentiated ACh-induced dilation, which was inhibited by potassium channel blockers. The findings suggested that resveratrol stimulates ACh-induced dilation through the NO and endothelium-derived hyperpolarizing factor pathways, but does not influence flow-mediated dilation via the cyclooxygenase pathway. These results highlighted the potential of resveratrol in improving vascular function under normal physiological conditions (Diaz et al., 2019). Resveratrol, extracted from *Reynoutria japonica* Houtt. [Polygonaceae], inhibits angiotensin II-induced cardiac fibroblast proliferation in a dose-dependent manner and activates NO signaling. It increases levels of NO, NOS, and cGMP while reducing mRNA expression of hypertrophic markers, atrial and brain natriuretic peptide (Wang et al., 2007). Nagaoka et al. also proposed that the effects of resveratrol on endothelium-dependent dilation are mediated by NO released from the endothelium, NOS activation via the ERK pathway, and subsequent activation of soluble guanylyl cyclase (Nagaoka et al., 2007). Doses of resveratrol resulted in a marked reduction in blood glucose, body weight, and plasma triglyceride levels, while increasing total plasma insulin levels. The mechanism involves enhancing eNOS expression, suppressing VEGF, and increasing phosphorylated p38 protein levels, indicating its role in mitigating inflammation and oxidative stress. Furthermore, resveratrol’s anti-inflammatory properties have contributed to improved cardiovascular outcomes by regulating multiple signaling pathways involved in insulin sensitivity and lipid metabolism (Yan et al., 2018). An *in vitro* study concluded that *trans*-resveratrol induces a concentration-dependent increase in both calcium and NO synthesis, potentially contributing to its vasorelaxant effects and enhancing endothelial function (Elíes et al., 2011). Klinge et al. have shown that nanomolar concentrations of resveratrol trigger the interaction between estrogen receptor alpha (ERα), caveolin-1 (Cav-1), and c-SRC, leading to NO production via a Gα-protein-coupled mechanism (Klinge et al., 2008). *In vivo* experiments revealed that resveratrol administration significantly increased muscle microvascular blood volume and flow in rats. These effects were negated by the NOS inhibitor L-NAME and systemic TNF-α infusion. This suggests that while resveratrol activates eNOS, its microvascular recruitment capabilities may be compromised by chronic inflammation, as seen in conditions like T2DM. Thus, while resveratrol holds promise for improving vascular function, its efficacy may be limited by inflammatory states (Wang et al., 2011). In experiments with HUVEC and EA. hy926 cells, resveratrol significantly upregulated eNOS mRNA expression in a time- and concentration-dependent manner, with increases observed up to 2.8-fold. Long-term exposure to resveratrol also elevated eNOS protein levels and NO production. Notably, resveratrol enhanced eNOS promoter activity, particularly within a crucial 263 bp proximal region, while stabilizing eNOS mRNA without altering the binding activity of key transcription factors (Wallerath et al., 2002). Another *in vitro* study confirmed that Resveratrol promotes NOS expression in cultured pulmonary artery endothelial cells (Hsieh et al., 1999). Numerous other studies have confirmed that resveratrol enhances NO levels, contributing to cardioprotection (Das et al., 2005; Soylemez et al., 2009; Dillenburg et al., 2013; Guillot et al., 2023).

Research focused on racemic analogues of the plant-derived indole alkaloid rhynchophylline and its stereoisomers (G2a and G2b) demonstrated that these compounds exhibit at least 30-fold greater potency in inducing vascular relaxation compared to rhynchophylline. Notably, G2a showed the greatest microvascular relaxation in rat mesenteric arteries, promoting endothelial function recovery by upregulating eNOS and increasing NO levels (Guo et al., 2017).

Rutaecarpine is an indolopyridoquinazolinone alkaloid derived from the fruit of *Tetradium ruticarpum* (A.Juss.) T.G.Hartley [Rutaceae] (Tian et al., 2019). Recent studies have demonstrated its significant role in enhancing NO synthesis by phosphorylating eNOS in human endothelial cells. This process is mediated through a transient receptor potential vanilloid type 1 (TRPV1)-dependent signaling pathway involving CaMKII and CaMKKβ/AMPK. Rutaecarpine has been shown to suppress inflammatory responses by inhibiting the expression of adhesion molecules like ICAM-1 and VCAM-1, which are often upregulated during inflammatory conditions (Lee et al., 2021).

An *in vitro* study on human umbilical vein endothelial cells (HUVEC) reported that rutin not only increases NO levels but also promotes the expression of basic fibroblast growth factor (bFGF) (Ugusman et al., 2014).

A study investigated the hypocholesterolemic and atheroscleroprotective effects of scutellarin, an active flavonoid from *Erigeron breviscapus* (Vaniot) Hand.-Mazz. [Asteraceae], administered at doses of 30 and 100 mg/kg/day. Results indicated that scutellarin effectively reduced serum total cholesterol levels elevated by an atherogenic diet and lowered the atherogenic index. Additionally, it enhanced NO production and improved endothelium-dependent vasorelaxation in isolated rat aortas, suggesting its potential to mitigate dietary cholesterol’s atherogenic effects (Li et al., 2009).

Secoisolariciresinol diglucoside, a plant lignan, shows potential in mitigating LPS-induced injury in HUVECs through its anti-inflammatory properties. In an LPS-stimulated model, this lignan significantly reduced levels of pro-inflammatory cytokines, including IL-1β, IL-6, and TNF-α, while enhancing NO release. Mechanistically, it inhibited NF-κB signaling and downregulated Akt expression, thereby contributing to its protective effects against HUVEC injury and apoptosis (Zhang S. et al., 2020).

Silibinin, a flavonoid derived from the plant milk thistle (Singla et al., 2024), administered at 50 mg/kg/day to ovariectomized rats, significantly modulated vascular function, comparable to 1 mg/kg/day of estrogen. Both treatments improved lipid profiles and reduced oxidative stress, leading to increased eNOS expression and decreased levels of inflammatory markers, such as angiotensin II and ET-1. Histopathological analysis demonstrated that silibinin and estrogen restored the normal endothelial layer in rat aortic tissue (Maleki et al., 2022).

Tanshinone IIA, a diterpene quinone derived from *Salvia miltiorrhiza* Bunge [Lamiaceae], commonly named Danshen, partially inhibited angiotensin II-induced cell proliferation and ET-1 expression while reducing ROS formation and ERK phosphorylation. Additionally, it enhanced NO production and eNOS phosphorylation, suggesting that the eNOS/NO pathway plays a crucial role in its cardioprotective mechanism (Chan et al., 2011).

Tilianin, a flavonoid glycoside from *Dracocephalum moldavica* L. [Lamiaceae], exhibits cardioprotective effects during myocardial ischemia/reperfusion injury by enhancing Na^+^/K^+^-ATPase and Ca^2+^-ATPase activities, which help alleviate calcium overload and improve energy metabolism. It also improves endothelial function by lowering ET-1 and thromboxane B2 levels while increasing CGRP levels, inhibits apoptosis by modulating B-Cell Lymphoma-2 (Bcl-2), Bcl-2-associated X protein (Bax), and caspase-3 expression, and suppresses NOD-like receptor protein 3 (NLRP3) inflammasome activation via the toll-like receptor 4 (TLR4)/NF-κB signaling pathway (Guo et al., 2015).

A study evaluated the effects of *trans*-dehydrocrotonin (t-DCTN), a diterpene from *Croton cajucara* Benth. [Euphorbiaceae], on hemodynamic parameters in rats. Intravenous administration of t-DCTN at 10 mg/kg resulted in a dose-dependent decrease in mean arterial pressure and heart rate, with its hypotensive effects linked to NO release rather than cholinergic or adrenergic pathways. Additionally, t-DCTN induced vasorelaxation in the aortic ring (Silva et al., 2005).

*Withania somnifera* (L.) Dunal [Solanaceae], commonly known as Ashwagandha, is a medicinal herb recognized for its stress-reducing and neuroprotective properties, primarily due to its active compound, the steroidal lactone withanolide A. This compound exhibits antioxidant and anti-inflammatory effects (Singh et al., 2010). A study investigated endothelial dysfunction using rat aortic rings and EA. hy926 endothelial cells. They found that *W. somnifera* and its active compound, withanolide A, promoted vasorelaxation by activating eNOS and enhancing NO signaling (Pathak et al., 2021).

Finally, a study evaluated 28 phenolic compounds for their capacity to enhance NO release in isolated porcine coronary arteries. Quercetin, myricetin, leucocyanidol, and oligomeric proanthocyanidins were found to have the most substantial effect on NO release, while caffeic acid and fisetin had a moderate impact. Effective phenols generally contained a flavan moiety with specific hydroxyl groups, with caffeic acid identified as essential for NO-dependent vasorelaxation (Taubert et al., 2002).

#### 3.1.2 Phytoextracts

Several studies on phytoextracts have targeted the L-arginine/NO/cGMP/K_ATP_ pathway for the treatment of cardiovascular diseases (Table 2).

**TABLE 2:** Phytoextracts modulating the L-arginine/NO/cGMP/K_ATP_ pathway in cardiovascular disease.

Extract	Plant/class	Method	*In vitro*/*in vivo* models	Mechanism and outcome	References
*Acanthospermum hispidum* DC.	Aqueous extract	Cardio-renal properties	*In vivo:* Male Wistar rats	↓Acute hypotension by the NO/cGMP pathway	Tirloni et al. (2017)
*Actinidia arguta* (Siebold and Zucc.) Planch. ex Miq., *Glycyrrhiza glabra* L., *Spatholobus suberectus* dunn, and *peucedanum praeruptorum* dunn	Total saponins, total flavonoids, total coumarins, and total flavonoids extracts	Vasodilating properties	*In vitro:* RAW 264.7 cell, Isolated rat thoracic aortas	Strong endothelium-dependent vasodilation via eNOS, Relaxation by modulating calcium	Zhang et al. (2017)
*Aronia melanocarpa* juice	Polyphenol-rich juice	Vascular protection	*In vitro:* Porcine coronary artery endothelial cells	↑NO, ↑eNOS phosphorylation and expression, Activating the PI3-kinase/Akt, JNK, and p38 MAPK pathways, Inactivation of transcription factors FoxO1 and FoxO3a	Kim et al. (2024b)
*Astragalus mongholicus* bunge	Polysaccharide extract	Cardiac hypertrophy	*In vivo:* Rat	↑NO, ↑cGMP, ↓Endothelial dysfunction, ↓Oxidative stress, ↓Inflammation	Han et al. (2016)
*Averrhoa bilimbi* L	Ethanolic extract	Hypertension study	*In vivo:* Ethanol-induced hypertensive rats	↑NO, ↑Vascular function, ↓Endothelial damage	Solfaine et al. (2021)
*Azadirachta indica* A. juss	Polyphenol-Rich Fraction	L-NAME-induced hypertension	*In vivo:* Hypertensive rats	↑Blood pressure, ↑oxidative stress markers, ↑Nrf2 expression	Omóbòwálé et al. (2019)
*Baccharis milleflora* DC.	Ethanol-soluble fraction	Cardiorenal effects study	*In vivo:* Rats	↑Diuretic and natriuretic effects, ↑NO availability, ↓Serum creatinine, ↓MDA levels	Klider et al. (2024b)
Berries: Black currant	Anthocyanin-rich extract	Vessel health in in vitro and in postmenopausal model	*In vitro:* Human endothelial cells *In vivo*: Ovariectomized rats	↑eNOS mRNA levels and NO synthesis through phytoestrogenic activity	Horie et al. (2019)
Berries: *Strawberry*	Aqueous extract	Vasorelaxation and endothelial function	*In vitro*: Rabbit aorta	↑Endothelium-dependent relaxation, involves phosphorylation of Akt and eNOS via PI3K/Akt pathway	Edirisinghe et al. (2008)
Berries: Strawberry and hawthorn	Aqueous extract of leaves	Vasodilatory potential	*In vitro:* Guinea pig hearts, rat aortic rings	Dose-dependent vasodilation, NO and cyclooxygenase-mediated	Mudnic et al. (2009)
Berries: Blueberry	Polyphenolic extract	Oxidative stress reduction	*In vitro*: Human aortic endothelial cells	↑NO, ↓Oxidative stress	Najjar et al. (2022)
Berries: Mulberry	Polyphenol extract	K-Ras-induced senescence study	*In vitro:* Smooth muscle cells	↓K-Ras, ↑iNOS, ↑AMPK, ↓Oxidative stress	Chen et al. (2022)
Berries: Red raspberry	Ethyl acetate extract	Hypertension reduction	*In vivo:* Hypertensive rats	↑NO, ↑SOD, ↓Blood pressure, ↓ET, ↓MDA.	Jia et al. (2011), Majewski et al. (2020)
Berries: Chokeberries	Extract: 15% anthocyanins and 48.5% of polyphenols as gallic acid equivalent	NO production study	*In vitro:* Bovine coronary artery endothelial cells	↑eNOS phosphorylation, ↑NO, ↑Vascular function	Varela et al., 2016; Olas (2018)
*Brillantaisia nitens* lindau	Aqueous, methylene chloride, methanol, and methylene chloride/methanol leaves extracts	Vasorelaxant effects	*In vitro:* Isolated rat vascular smooth muscle	Concentration-dependent vasorelaxation, inhibition of Ca^2+^ influx, Activation of ATP-sensitive K^+^ channels	Dimo et al. (2007)
*Camellia sinensis* (L.) kuntze	Aqueous extract	Hypertension, endothelial dysfunction	*In vitro:* HUVEC, RAEC, SHR aortic rings	↑NO, ↑K^+^ channel modulation	Persson et al. (2006)
	cystatin named CsCPI1	Thrombotic and Stroke model	*In vivo*: C57BL/6 J mice, Left carotid artery	Inhibiton of the formation of thrombus and prevention of the occurrence of ischemic stroke ↑NO and inhibiting platelet aggregation, ↑Occludin, ↓TNF-α, ↓IL-6, ↓ CXCL1	Fang et al. (2023)
*Camellia japonica* L	Ethanolic extract of fruits	Endothelium-dependent vasorelaxation study	*In vitro:* Porcine coronary arteries	↑eNOS phosphorylation ↓Vascular smooth muscle cell migration	Park et al. (2015)
*Centella asiatica* (L.) Urb. [Apiacea]	Ethanolic extract	Hypertensive rat model	*In vivo:* Male rats	↑NO ↓ACE, ↓ROS, ↓MDA, ↓Systolic blood pressure	Bunaim et al. (2021)
*Chiranthodendron pentadactylon* larreat	Aqueous Extract	Hypotensive and vasorelaxant effects	*In vivo:* Anesthetized rats	↑NO activity, ↑Vascular reactivity	Escobar-Ramírez et al. (2023)
*Clitoria ternatea* L	Aqueous extract of flowers	Cardiac protection in L-NAME-induced hypertension	*In vivo:* Hypertensive rats	↓Hypertension, ↑Vasorelaxation, ↓Left ventricular hypertrophy, ↓Renin-angiotensin system	Maneesai et al. (2021)
*Croton urucurana* baill	Ethanol-soluble fraction	Cardiovascular function in SHRs	*In vivo:* Hypertensive rats	↑Hemodynamics, ↑Electrocardiographic profiles, ↑Renal function, ↑vasodilation via NO and potassium channels	Auth et al. (2022)
Danshen-Shanzha formula (root of *S. miltiorrhiza* and the fruit of *C. pinnatifida* var. *major*)	Ethanol extract	Anti-atherosclerotic study	*In vivo*: SHR Rats	↑HDL-C, ↓IL-1β, ↓IL-18, ↑NO, ↓Cholesterol, ↓Triglycerides, ↓LDL-C	Zhang et al. (2019)
*Delonix regia* (Bojer ex Hook.) Raf	80% ethanol extract of leaves	Heart injury protection	*In vivo:* Mouse model of isoproterenol-induced heart injury	↑NO, ↑Vasodilation in porcine coronary arteries ↓Mortality, ↓Cardiac hypertrophy, ↓Creatine phosphokinase, ↓LDH, ↓TNF-α	Wang et al. (2016a)
*Dicksonia sellowiana* (C.Presl) Hook	Hydroalcoholic extract of leaves	Relaxation response, Blood pressure effects	*In vitro:* Rat aortic rings *In vivo:* Anesthetized rats	Activation of muscarinic receptors, Stimulation of the NO pathway and opening of Ca^2+^-activated K^+^ channels	Rattmann et al. (2009)
*Diospyros virginiana* L	Methanolic extract	Endothelial function study	*In vitro*: HUVEC	↑Phosphorylation of Akt and eNOS, ↑NO, ↓ET-1	Woodcock et al. (2013)
*Dracocephalum heterophyllum* Benth	Total flavonoid extract	Cardiomyocyte hypertrophy model	*In vitro:* Rat cardiomyocytes	Regulates Ca^2+^, ↑NO, ↓Hypertrophic markers	Jiang et al. (2018)
*Elsholtzia splendens* Nakai ex F.Maek	Total flavonoid extract	Vasorelaxation via NOS pathway	*In vitro:* Rat thoracic aortas	↑NO-mediated vasorelaxation, ↑Involvement of K_ATP_ channels	Wang et al. (2014)
*Erigeron breviscapus* (Vaniot) Hand.-Mazz	Polyphenol-enriched fraction	Cardiovascular protection	*In vivo:* High-fat diet rats	↓Adipose tissue, ↓Hepatic lipid, Modulation NO pathways and K^+^ channels	Wang et al. (2016b)
*Eruca sativa* mill	Methanolic extract	L-NAME and atropine induction	*In vivo:* Normotensive and hypertensive rats *In vitro:* Aortic rings	↑NO, ↑Endothelium-dependent vasorelaxation ↓MAP	Salma et al. (2018)
*Erythroxylum passerinum* mart	Ethanolic extract	Vasorelaxation study	*In vitro:* Mesenteric artery rings *In vivo:* Rats	Hypotension and endothelium-dependent and independent vasorelaxation, NO and K^+^channels	Santos et al. (2022)
*Foeniculum vulgare* Mill	Seeds extract	Vasodilatory response study	*In vitro:* Aortic rings	↑Vasodilation via NO/cGMP pathway, K^+^ channels, and muscarinic receptors	Zahi et al. (2025)
*Gynostemma pentaphyllum* (Thunb.) makino	Saponin extract	Vasorelaxation study	*In vitro:* Porcine coronary rings	↑Vasorelaxation, ↑NO	Tanner et al. (1999)
Jinhe yangxin	Total flavonoid extract	Myocardial ischemia protection	*In vivo:* Rat model	↑p-Akt, ↑GSK-3β, ↑eNOS, ↑NO, ↑SOD, ↑GSH-Px ↓CK, ↓ST elevation, ↓MDA, ↓cTnI, ↓LDH, ↓IL-6, ↓TNF-α; ↓Apoptosis	Cheng et al. (2018)
*Ligusticum chuanxiong* Hort	Ethanolic extract	Vascular protection study	*In vivo:* OVX Rats	↓LDL cholesterol, ↑HDL cholesterol, ↑eNOS mRNA expression	Li et al. (2013)
*Mandevilla moricandiana* (A.DC.) Woodson	Hydroalcoholic extract of leaves	Vasodilation study	*In vitro:* NO/cGMP pathway activation	↑NO-mediated vasodilation	Ferreira et al. (2017)
*Millettia pulchra* (Voigt) Kurz	Total flavonoid extract	Cardioprotection in MI/RI models	*In vitro:* Hypoxia/reoxygenation *In vivo:* MI/RI rats	↑cNOS, ↑ATPases, ↑Hemodynamics, ↓LDH, ↓iNOS activities, ↓Oxidative damage, ↓Cardiocyte apoptosis	Huang et al. (2015)
	Yulangsan Flavonoids	Myocardial ischemia/reperfusion injury	*In vivo:* Rat model	↓Infarct size, ↓iNOS, ↓Caspase-3 levels, ↑NOS activity, ↓Inflammation	Zhang et al. (2014)
*Morus alba* L	aqueous and ethanolic extract	Arterial hypertension model	*In vivo:* Rats	↑NO ↓Arterial hypertension	Aghdam et al. (2024)
*Nardostachys jatamansi* (D.Don) DC.	Essential oil	Vasodilation study	*In vitro*: HUVECs	↑p-Akt, ↑NO, ↑eNOS phosphorylation, ↑Intracellular Ca^2+^ level	Maiwulanjiang et al. (2015)
Oat Protein	Protein diet	Hypertension study	*In vivo:* SHR Rats	↓Blood pressure, ↑NO ↓ACE, ↓TNF-α	Raj et al. (2024)
Red wine	Polyphenolic extract (alcohol free)	Acetylcholine-induced vasorelaxation	*In vitro*: HUVEC and EA.hy926 cells *In vivo*: Rat model	↑NO ↑eNOS, ↓Oxidative stress	Leikert et al. (2002)
	Flavonoid-rich diet	Endothelium-dependent vasorelaxation	*In vitro*: Aortic rings of rats	↑NO, ↑NOS, ↑cGMP	Benito et al. (2002)
	Red wine polyphenolic extract	Cardiac reactivity study, Ischaemia-reperfusion injury	*In vitro*: Isolated perfused heart *In vivo*: Rats	↑Nitrite ↓Oxidative stress, ↓MDA+4-HNE, ↓Infarct size	Ralay Ranaivo et al. (2004)
Rice Bran Phenolic	Polyphenolic extract	Antioxidant and anti-inflammatory study	*In vitro:* HUVEC	↑Nrf2, ↑NQO1, ↑HO1, ↑eNOS ↓ICAM1, ↓CD39, ↓CD73, ↓NOX4	Saji et al. (2019)
	Polyphenolic extract	Vasorelaxant effect	*In vitro:* Endothelial cells	↑_P_-eNOS, ↑NO, ↑p-Src	Seong et al. (2022)
Fermented soy	Aglycone-rich biotransformed extract	Atherosclerosis	*In vitro:* HUVEC cells	↑NO, ↑Endothelin-1, ↑Prostaglandin E2	De Andrade et al. (2015)
*Parkia speciosa* Hassk	Methanolic extract	Hypertension and changes in heart	*In vivo:* Rats	↑NO ↓Oxidative stress, ↓Blood pressure	Kamisah et al. (2017)
	Ethanolic extract	Cardiac hypertrophy	*In vitro:* H9c2 Cells	↑SOD ↓NADPH oxidase, ↓ B-type natriuretic peptide levels, ↓ROS ↓P-ERK, ↓P-p38, ↓P-JNK	Siti et al. (2021a)
*Piper guineense* Schumach. and Thonn	Polyphenolic extract	Hypertension study	*In vivo:* Hypertensive rats	↑NO ↓Blood pressure, ↓ACE, arginase, ↓MDA	Olopade et al. (2024)
*Piper sarmentosum* Roxb. (Kadukmy™)	Polyphenolic extract	Hypertension study	*In vivo:* Hypertensive rats	↑NO, ↓Systolic/diastolic blood pressure, ↓MDA, ↓Cholesterol levels	Mohd Zainudin et al. (2015)
*Polygala paniculata* L	Flavonoid extract	L-NAME and methylene blue induction	*In vitro:* Aortic rings *In vivo:* Rats	↓Vasodilation ↓Hypertension	Lapa et al. (2011)
Pomegranate extract (Pomanox®)	Polyphenolic extract	Coronary function in dyslipidemic pigs	*In vivo:* Pigs	↑Endothelial relaxation, ↑Akt/eNOS pathway activation ↓Oxidative stress markers	Vilahur et al. (2015)
Pumpkin	Seed oil	Antihypertensive and cardioprotective study	*In vivo:* L-NAME-induced hypertensive rats	↑NO ↓Blood pressure, ↓MDA	El-Mosallamy et al. (2012)
*Rheum officinale* baill	Aqueous extract	Blood circulation and thrombosis study	*In vitro*	↑Thrombosis inhibition ↓ROS, Regulation of NOS2 and NOS3	Zhang et al. (2022)
*Sarcopoterium spinosum* (L.) Spach	Ethanolic extract	Endothelial dysfunction	*In vitro:* HECV cells	↑NO, ↓ROS, ↓LPO	Zbeeb et al. (2024)
*Schisandra chinensis* (Turcz.) Baill	Chloroform and metanol extract	Vasorelaxation study	*In vitro:* Rat thoracic aorta	Vascular relaxation by NO signaling and direct dephosphorylation of MLC in vascular smooth muscle cells	Park et al. (2009)
*Senecio serratuloides* DC	Hydro-ethanol extract	Antihypertensive effects	*In vivo:* Mice	↑NO, ↑Ang II ↓Blood pressure, ↓LDL, ↓Triglycerides	Tata et al. (2019)
*Sesamum radiatum* Schum. and Thonn	Aqueous extract	Vasorelaxation study	*In vitro*: Guinea pig aortic preparations	↑Vasorelaxation via NO and K_ATP_ channels	Konan et al. (2008)
*Sophora flavescens* aiton	Ethanol extract	Vascular smooth muscle relaxation	*In vitro:* Endothelial cells	↑Relaxation via the NO-sGC-cGMP signaling pathway	Jin et al. (2011)
*Terminalia fagifolia* mart	Ethanol extract	Vasorelaxation study	*In vitro:* Thoracic aorta rings of rats	Concentration-dependent vasorelaxation via NO/sGC/cGMP pathway and K^+^ channels	de Carvalho et al. (2019)
*Terminalia pendula* var. *pendula*	Aqueous methanol extract	Myocardial infarction and hypertension	*In vivo:* Hypertensive rats and AMI model	Improve left ventricular hypertrophy, ↓Myocardial necrosis, ↓Inflammation	Saqib et al. (2020)
*Urtica dioica* L	Aqueous extract	Hypotensive responses	*In vitro*: Thoracic aortae, Guinea-pig atria *In vivo*: Rats	Vasorelaxing effect via NO and K^+^ channels	Testai et al. (2002)
*Vitex agnus-castus*	Ethanol extract	Arterial relaxation study	*In vitro:* Rabbit arterial rings	↑Relaxation via NO/cGMP and prostaglandin synthesis pathways	Thaçi et al. (2022)
*Vitex doniana* Sweet	Aqueous extract	Vasorelaxant activity	*In vitro:* Rat aortic rings	↑NO/sGC-mediated relaxation ↓Mean arterial pressure	Dongmo et al. (2011)
Wild Mediterranean	Phenolic-rich extracts	NO release study	*In vitro:* Porcine aortic endothelial cells	↑NO release in endothelial and brain cell membranes	Grande et al. (2004)
*Ziziphus jujuba* Mill	Water and ethyl acetate extract	Hypertension inhibition	*In vivo:* Hypertensive rats	↑NO ↓Hypertension progression	Mohebbati et al. (2020)
*Ziziphus oxyphylla* Edgew	Hydro-methanolic extract	L-NAME-induced hypertensive rat	*In vivo*: hypertensive rat model induced by L-NAME.	↑NO, ↑cGMP, ↑eNOS ↓IL-6, ↓TNF-α, ↓ACE	Shaukat et al. (2022)

6-keto-PGF1α, 6-Keto-Prostaglandin F1 alpha; AC/cAMP/PKA, Adenylate Cyclase/Cyclic AMP/Protein Kinase A; ACE, Angiotensin-Converting Enzyme; ACh, Acetylcholine; AhR-RGE, Aryl Hydrocarbon Receptor-Regulated Gene Enhancement; Ahr-TN, Aryl Hydrocarbon Receptor-Translocated; AI, atherogenic index; Akt, Protein Kinase B ALDH, aldehyde dehydrogenase; AMPK, AMP-Activated Protein Kinase; Ang II, Angiotensin II; Ang II-CFP, Angiotensin II; Central Fusion Point; AP-1, Activator Protein 1; ARNT, aryl hydrocarbon receptor nuclear translocator; AS, atherosclerosis; ATPases, Adenosine Triphosphatases; Bcl-2/Bax, B-cell Lymphoma 2/Bcl-2-associated X Protein; bFGF, basic fibroblast growth factor; BMI, body mass index; BP, blood pressure; BRS, baroreflex sensitivity; CaMKII, Calcium/Calmodulin-Dependent Protein Kinase II; CaMKKβ, Calcium/Calmodulin-Dependent Protein Kinase Kinase Beta CAT, catalase; CD39, Cluster of Differentiation 39; CD73, Cluster of Differentiation 73; CDR, cognitive decline rate; cGMP, cyclic guanosine monophosphate; CK, creatine kinase; CLS, Capillary-like Structures; cNOS, constitutive nitric oxide synthase; CPK, creatine phosphokinase; CS, Cholesterol SulfatecTnI, Cardiac Troponin I; CVP, central venous pressure; CYP7A1, Cholesterol 7 Alpha-Hydroxylase; ECFC, Endothelial Colony-Forming Cells; EDHF, Endothelium-Derived Hyperpolarizing Factor; EDR, Endothelium-Dependent Relaxation; EDV, end diastolic volume; eNO, endothelial nitric oxide; eNOS, endothelial nitric oxide synthase; ERK1/2, Extracellular Signal-Regulated Kinases 1/2,ET-1, Endothelin-1; GC, guanylate cyclase; GSH/GSSG, Reduced Glutathione/Glutathione Disulfide Ratio; GSH-Px, Glutathione Peroxidase; GSK-3β, Glycogen Synthase Kinase 3 Beta; H_2_O_2_, hydrogen peroxide; Hcy-ED, Homocysteine-Endothelial Dysfunction; HDL, High-Density Lipoprotein; HDL-C, High-Density Lipoprotein Cholesterol; HO-1, Heme Oxygenase 1; ICAM-1, Intercellular Adhesion Molecule 1; IL-1β, Interleukin 1 Beta; IL-6, Interleukin 6; iNOS, inducible nitric oxide synthase; IP3R, Inositol 1,4,5-Trisphosphate Receptor,JNK, c-Jun N-terminal kinase,JNK1/2, c-Jun N-terminal kinases 1/2; K_ATP_, ATP-Sensitive Potassium Channel; KCM, potassium channel modulation; KDR, kinase insert domain receptor; K-Ras, Kirsten Rat Sarcoma Viral Oncogene Homolog; KV; and K_ATP_, Voltage-Gated Potassium and ATP-Sensitive Potassium Channels; LDH, lactate dehydrogenase; LDL, Low-Density Lipoprotein; LDL-C, Low-Density Lipoprotein Cholesterol; LF and HF, low frequency and high frequency components of power index; LPO, lipid peroxidation; MAP, mean arterial pressure; MAPK, Mitogen-Activated Protein Kinase; MDA, Malondialdehyde,MF, myocardial fibrosis; mitoK_ATP_, Mitochondrial ATP-Sensitive Potassium Channel; MMP, matrix metalloproteinase; mRNA, messenger ribonucleic acid; NADPH, nicotinamide adenine dinucleotide phosphate; NLRP3, NLR; Family Pyrin Domain Containing 3; NO, Nitric Oxide,NOS2 and NOS3, Nitric Oxide Synthase 2 and 3; NOX4, NADPH; Oxidase 4; NQO1, NAD(P)H Quinone Dehydrogenase 1; NRC, nuclear regulatory commission; Nrf2, Nuclear Factor Erythroid 2–Related Factor 2; OS, oxidative stress; p38 MAPK, p38 Mitogen-Activated Protein Kinases; p47phox, Phox Subunit of NADPH; oxidase; PE, phenylephrine; PGI, prostacyclin; PI3K, Phosphoinositide 3-Kinase,p-NF-κB, Phosphorylated Nuclear Factor kappa B,PTEN, phosphatase and tensin homolog; RAS, Renin-Angiotensin System; ROS, reactive oxygen species; RS, renal stenosis; SAL, salinity; sGC, soluble guanylate cyclase; SIPS, Stress-Induced Premature Senescence; Smad2/3, SMAD; Family Member 2/3; SOD, superoxide dismutase; STCL, short term cellular leukemia; TG, Triglycerides,TGF-β1, Transforming Growth Factor Beta 1; TNFR, Tumor Necrosis Factor Receptor,TNF-α, tumor necrosis factor alpha; TPE, total protein excretion; TRPA1, Transient Receptor Potential Ankyrin 1; TXB2, Thromboxane B2; VCAM-1, Vascular Cell Adhesion Molecule 1; VD, vascular diameter; VEGF, vascular endothelial growth factor; VOCC, Voltage-Operated Calcium Channels; VP, venous pressure; VR, vascular resistance; β2-AR, Beta-2; adrenergic receptor; β-Gal, Beta-Galactosidase.

The findings suggested that intraduodenally administered *Acanthospermum hispidum* DC. [Asteraceae] in rats has a notable acute hypotensive effect mediated by the NO/cGMP pathway, highlighting its therapeutic potential for managing acute hypertension (Tirloni et al., 2017).

Evaluation of the effects of total saponins from *Actinidia arguta* (Siebold and Zucc.) Planch. ex Miq. [Actinidiaceae], total flavonoids from *Glycyrrhiza glabra* L. [Fabaceae] and *Spatholobus suberectus* Dunn [Fabaceae], and total coumarins from *Peucedanum praeruptorum* Dunn [Apiaceae] exhibited potent anti-inflammatory effects and vasodilator responses mediated by eNOS in isolated aortic rings (Zhang et al., 2017).

The juice of *Aronia melanocarpa* (Michx.) Elliott [Rosaceae] is rich in polyphenols, which possess potent antioxidant properties. These compounds, at a dose of 16.8 µg, not only scavenge free radicals but also modulate signaling pathways that regulate eNOS activity. The effects of *A. melanocarpa* are mediated through several key pathways, including the PI3K/Akt pathway, the JNK and p38 MAPK pathways, as well as the transcription factors FOXO1 and FOXO3a (Kim et al., 2024b).

*Astragalus* polysaccharide, derived from the herb *Astragalus mongholicus* Bunge [Fabaceae], has demonstrated significant protective effects against cardiovascular disease, particularly in isoproterenol-induced cardiac hypertrophy. In a study involving rats, administration of this polysaccharide (400 and 800 mg/kg) in conjunction with isoproterenol (10 mg/kg) resulted in significant improvements in endothelial function and a reduction in cardiac hypertrophy. Its protective mechanisms were linked to decreased numbers of circulating endothelial cells, reduced oxidative stress, and enhanced NO and cGMP production, alongside anti-inflammatory effects indicated by lower levels of TNF-α and IL-6 (Han et al., 2016).

*Averrhoa bilimbi* L. [Oxalidaceae], commonly known as bilimbi, is a tropical fruit-bearing tree. The 40 g/kg *A bilimbi* extract significantly increased serum NO levels and enhanced vascular function in ethanol-induced hypertensive rats. Additionally, it inhibited endothelial pyknosis and reduced leukocyte infiltration, indicating protective effects on the vascular system (Solfaine et al., 2021).

Researchers examined the protective effects of orally administering a polyphenol-rich fraction from *Azadirachta indica* A. Juss. [Meliaceae] (100 and 200 mg/kg, for 21 days) against hypertension and cardiorenal dysfunction induced by L-NAME in rats. Results indicated that this fraction effectively restored blood pressure levels, improved oxidative stress markers, and enhanced the expression of protective factors, such as Nrf-2 (Omóbòwálé et al., 2019).

In their study, Klider et al. examined the cardiorenal effects of the ethanol-soluble fraction of *Baccharis milleflora* DC. [Asteraceae], a Brazilian plant, was identified to contain 33 metabolites, including various phenolic compounds, in rats. The findings revealed that this fraction significantly enhanced diuretic and natriuretic effects while maintaining potassium levels. Furthermore, it lowered serum creatinine and MDA levels while increasing nitrite levels, indicating improved NO availability. The diuretic effects were associated with the activation of the NO/cGMP pathway (Klider et al., 2024b).

Phytoestrogens, plant-derived compounds with estrogenic activity, are found in various foods, including black currant extract (BCE), which is rich in anthocyanins. Results of an *in vitro* study indicated that BCE and anthocyanins significantly upregulated eNOS mRNA levels, enhancing NO synthesis in EA. hy926 cells, while the estrogen receptor antagonist (fulvestrant) inhibited these effects. In ovariectomized rats, a model for menopause, BCE also increased eNOS protein expression and NO synthesis (Horie et al., 2019). Freeze-dried strawberry powder, rich in polyphenols, would induce endothelium-dependent relaxation in the rabbit aorta. The aqueous extract of strawberry powder caused a dose-dependent relaxation in aortic rings precontracted with norepinephrine, achieving a maximum relaxation similar to that of ACh. The relaxation effect was dependent on eNOS and was confirmed to involve Akt and eNOS phosphorylation via the PI3K/Akt signaling pathway (Edirisinghe et al., 2008). Using isolated guinea pig hearts and rat aortic rings, researchers compared the vasodilatory potential of strawberry leaf extract with that of hawthorn (*Crataegus oxyacantha* Walter [Rosaceae]) extract. Both extracts demonstrated similar dose-dependent vasodilation, with maximal relaxation rates of 72.2% for strawberry and 81.3% for hawthorn. The study found that the vasodilatory effect of strawberry leaves is endothelium-dependent and mediated by NO and cyclooxygenase products, indicating its potential as an effective natural vasodilator (Mudnic et al., 2009). Also, polyphenols of blueberry extract (200 μg/mL) functioned via an Nrf2-dependent mechanism to diminish oxidative stress and elevate NO levels in HAECs treated with angiotensin II (Najjar et al., 2022). Another berry polyphenol extract, mulberry, mitigated K-Ras-induced senescence in smooth muscle cells by reducing K-Ras levels and phosphorylated ERK, while enhancing cyclin and cyclin-dependent kinase activation. It downregulated K-Ras-induced cyclin-dependent kinase inhibitors (CDKIs) and increased phosphorylated AMPK and iNOS levels. The reversal of its effects by L-NAME or AMPK inhibitors suggested that its mechanism operates through an iNOS and AMPK-dependent pathway (Chen et al., 2022). Treatment of SHR with 200 mg/kg of ethyl acetate extract of red raspberry fruit for 5 weeks reduced blood pressure, enhanced serum NO and SOD levels, and decreased ET and MDA levels (Jia et al., 2011). Majewski et al. also demonstrated that raspberry seed supplementation decreased liver enzyme levels and enhanced hydrogen peroxide scavenging capacity in young Wistar-Kyoto rats and SHR models. These seeds decreased the atherogenic index and enhanced vasodilation via iNOS and cyclooxygenase-2 (COX-2) pathways (Majewski et al., 2020). *Aronia* berries, commonly known as chokeberries, are recognized for their high antioxidant content and potential health benefits (Olas, 2018). Varela et al. examined the effects of acute and chronic treatment with *Aronia* extracts on NO production and eNOS phosphorylation in bovine coronary artery endothelial cells. It was found that low concentrations of *Aronia* extract (0.1 μg/mL) significantly enhanced NO synthesis and eNOS phosphorylation after just 10 min of treatment. Additionally, prolonged exposure to *Aronia* extract for 48 h followed by a short re-treatment (10 min) further increased eNOS sensitivity and NO production (Varela et al., 2016).

*In vitro* experiments on rat vascular smooth muscle demonstrated that extracts of *Brillantaisia nitens* Lindau [Acanthaceae] induced concentration-dependent relaxation of norepinephrine-induced contractions in aortic strips. This extract’s vasorelaxation effect was endothelium-independent and not mediated by PGI2 or NO, as indicated by the lack of significant changes in relaxation after pretreatment with indomethacin or L-NAME. However, the relaxation was significantly inhibited by tetraethylammonium and glibenclamide, suggesting the involvement of non-selective and ATP-sensitive potassium channels. Furthermore, the extract inhibited calcium-induced contractions and calcium influx in aortic strips, indicating that its vasorelaxant effects are primarily due to the inhibition of calcium influx through these channels (Dimo et al., 2007).

Incubation of HUVEC cells with green tea, black tea, and catechin tea for 10 min resulted in significant, dose-dependent inhibition of ACE activity. Furthermore, after 24 h of incubation with these teas, a notable dose-dependent effect on HUVEC production was also observed (Persson et al., 2006). Recent research identified a cystatin, CsCPI1, a cysteine proteinase inhibitor from the tea plant Camellia sinensis (L.). Kuntze [Theaceae], which exhibits significant antithrombotic properties. CsCPI1 has demonstrated therapeutic benefits in mouse models of thrombotic diseases and ischemic stroke, primarily by enhancing endothelial NO production and inhibiting platelet aggregation (Fang et al., 2023). Another research explored the effects of three distinct tea cultivars—“Yabukita,” “Sofu,” and “Sunrouge”—on NO production and related protein expression in the aorta of SHRs on a high-salt diet. The study found that all three tea infusions effectively prevented the reduction in urinary NO metabolites, which are indicative of overall NO production, caused by high-salt intake. Notably, only “Yabukita” and “Sofu” were observed to enhance the expression of sGC in the thoracic aorta. These findings suggested that variations in flavonoid composition among tea cultivars result in differing effects on NO signaling pathways in SHRs, highlighting the importance of other beneficial compounds beyond epigallocatechin 3-gallate (EGCG) in promoting cardiovascular health (Nomura et al., 2017).

The fruit extract of *Camellia japonica* L. [Theaceae] demonstrated significant endothelium-dependent vasorelaxation in porcine coronary arteries, primarily through the PI3K/eNOS/NO pathway. The extract not only induces eNOS phosphorylation but also inhibits the proliferation and migration of human vascular smooth muscle cells (VSMCs) stimulated by TNF-α and platelet-derived growth factor BB (PDGF-BB) (Park et al., 2015).

*Centella asiatica* (L.) Urb. [Apiaceae] is a versatile herb with a rich history in traditional medicine and modern skincare (Witkowska et al., 2024). The ethanolic extract of *Centella asiatica* showed significant antihypertensive effects in a rat model treated with L-NAME. Over 8 weeks, a 500 mg/kg dose prevented increases in systolic blood pressure and improved serum NO levels. The extract also enhanced antioxidant activity, evidenced by lower MDA levels and cardiac ACE activity. Furthermore, it positively influenced the renin-angiotensin-aldosterone system, mitigating the harmful effects of angiotensin II and supporting endothelial function (Bunaim et al., 2021).

*Chiranthodendron pentadactylon* Larreat [Malvaceae] exhibits hypotensive and vasorelaxant effects through its methanolic extract and the active compound cyanidin 3-*O*-glucoside. In anesthetized rats, they lower the heart rate and mean arterial pressure. The mechanism involves the synthesis and release of NO, while cholinergic receptors and prostaglandins do not play a role. Additionally, isolated rat aortic rings and mesenteric arterial beds demonstrated these effects, highlighting the extract’s potential in treating blood pressure conditions (Escobar-Ramírez et al., 2023). In this context, Fushimi et al. also found that cyanidin 3-*O*-glucoside enhances blood flow and stimulates angiogenesis in skeletal muscle, thereby contributing to reduced blood pressure (Fushimi et al., 2023).

*Clitoria ternatea* L. [Fabaceae], commonly known as butterfly pea, blue pea, or Asian pigeonwings, is a perennial herbaceous plant (Aleena et al., 2023). In a study, the effects of *Clitoria ternatea* extract on cardiac and vascular dysfunction induced by L-NAME in rats were investigated. Results indicated that 300 mg/kg of this extract significantly reduced hypertension, improved vasorelaxation, and mitigated left ventricular hypertrophy. The extract’s protective effects were attributed to decreased oxidative stress and modulation of the renin-angiotensin system (Maneesai et al., 2021).

*Croton urucurana* Baill. [Euphorbiaceae], commonly known as “sangra d'água,” is a medicinal tree species (Auth et al., 2022). A study examined the cardiovascular effects of the ethanol-soluble fraction of *C. urucurana* in SHRs. The rats were administered doses of 30, 100, and 300 mg/kg for 28 days. The treatment resulted in significant improvements in hemodynamic parameters, electrocardiographic profiles, and renal function, effectively reversing the impairments associated with hypertension. Additionally, the ethanol-soluble fraction exhibited a dose-dependent vasodilatory effect in the mesenteric vascular beds at concentrations of 0.1, 0.3, and 1 mg. This vasodilation was inhibited by removing the endothelium or by inhibiting NOS. The underlying mechanism of the extract’s action involved the activation of NO and calcium-activated small conductance potassium channels (Lopes et al., 2022).

Danshen and Shanzha, key herbs in traditional Chinese medicine, were studied for their anti-atherosclerotic effects using network pharmacology, revealing 41 target proteins and 16 relevant pathways. The danshen-shanzha formula significantly improved lipid profiles and reduced intima-media thickness. It also demonstrated anti-inflammatory properties by lowering IL-1β and IL-18 levels, while enhancing endothelial function. Histopathological analyses supported its protective effects on arterial health (Zhang et al., 2019).

*Delonix regia* (Bojer ex Hook.) Raf. [Fabaceae], a flowering plant in the pea family, has been traditionally used in folk medicine for various ailments (Modi et al., 2016). In a mouse model of heart injury induced by isoproterenol, the leaf extract administration at 400 mg/kg significantly reduced mortality rates and cardiac hypertrophy. Biochemical analyses revealed decreased levels of serum markers, including creatine phosphokinase, LDH, and TNF-α, alongside increased NO levels, indicating enhanced vasodilation and reduced myocyte injury. Additionally, *in vitro* experiments demonstrated that it induced dose-dependent vasodilation in porcine coronary arteries (Wang L.-S. et al., 2016).

*Dicksonia sellowiana* (C.Presl) Hook. [Cyatheaceae], a native plant from America, is rich in polyphenols and antioxidants. The hydroalcoholic extract from its leaves has shown significant vascular effects, inducing relaxation of rat aortic rings and causing reversible hypotension in anesthetized rats. The relaxation of these effects was linked to the activation of muscarinic receptors and the NO pathway, with contributions from calcium-activated potassium channels (Rattmann et al., 2009).

Treatment of HUVEC cells with extract of *Diospyros virginiana* L. [Ebenaceae], common name persimmon, resulted in a significant increase in the phosphorylation of Akt and eNOS, along with elevated levels of NO metabolites. Additionally, there was a notable reduction in ET-1 secretion into the media after 24 h (Woodcock et al., 2013).

The flavonoids isolated from *Dracocephalum heterophyllum* Benth. [Lamiaceae], a traditional Tibetan and Uyghur medicinal plant, has shown potential in mitigating cardiomyocyte hypertrophy induced by angiotensin II. Angiotensin II increased hypertrophic markers, cell surface area, and intracellular calcium concentration while reducing NOS activity and NO production. It reversed these effects in a concentration-dependent manner, suggesting its mechanism involves regulating calcium and enhancing NO release, thereby alleviating hypertrophy (Jiang et al., 2018).

A study investigated the vasorelaxant effects of total flavonoids from *Elsholtzia splendens* Nakai ex F. Maek. [Lamiaceae], a plant rich in flavonoids, on rat thoracic aortas using an organ bath system. The results demonstrated that these flavonoids induced concentration-dependent vasorelaxation in endothelium-intact aortic rings, an effect diminished when the endothelium was removed. The involvement of the NOS pathway was confirmed, as the NOS inhibitor significantly blocked the vasorelaxation effect. Additionally, endothelium-independent mechanisms were suggested, as the K_ATP_ channel blocker reduced this relaxation (Wang et al., 2014).

Erigerontis Herba, also known as *Erigeron breviscapus* (Vaniot) Hand.-Mazz. [Asteraceae] is a traditional Chinese medicinal herb recognized for its various therapeutic properties, particularly in cardiovascular health. Erigerontis Herba significantly reduced adipose tissue and hepatic lipid levels in rats fed a high-fat diet, indicating its potential in managing obesity and hepatic steatosis. Additionally, it demonstrated vasodilative effects through NOS pathways and other channels, BKca, Kv, and Kir channels. Also, it modulated the expression levels of CD36, Cyp7α1, and PPAR-γ (Wang Y. P. et al., 2016).

Intravenous administration of the extract *Eruca sativa* Mill [Brassicaceae] significantly reduced mean arterial pressure in both normotensive and hypertensive rats, with the antihypertensive effect being mediated by muscarinic receptors linked to NO release. *In vitro* experiments demonstrated endothelium-dependent relaxation in aortic rings, which was partially inhibited by L-NAME and atropine, indicating the involvement of NO. Additionally, the extract exhibited both vasodilatory and cardiac effects, supporting its traditional use for hypertension management due to its ability to modulate calcium influx and release in vascular tissues. The presence of bioactive compounds, such as quercetin and erucin, further substantiates these findings (Salma et al., 2018).

The ethanolic extract of *Erythroxylum passerinum* Mart. [Erythroxylaceae] was evaluated for its cardiovascular effects, demonstrating notable hypotension, bradycardia, and vasorelaxation in rats. Phytochemical analysis revealed the presence of polyphenols, while alkaloids were absent. The vasorelaxation effect induced by this ethanol extract was significantly inhibited by L-NAME and indomethacin, suggesting the involvement of NO signaling pathways. Furthermore, the removal of the endothelium and treatment with potassium channel blockers reduced the vasorelaxation response, indicating the critical role of potassium channels (Santos et al., 2022).

The aqueous extract of *Foeniculum vulgare* Mill. [Apiaceae], common name French fennel, demonstrated a concentration-dependent vasodilatory response, significantly reduced by endothelium removal and various inhibitors (L-NAME, ODQ, indomethacin, tetraethylammonium, glibenclamide, and atropine), indicating involvement of the NO/cGMP pathway, potassium channels, and muscarinic receptors (Zahi et al., 2025).

The extract of *Gynostemma pentaphyllum* (Thunb.) Makino [Cucurbitaceae], also known as Southern Ginseng, induced concentration-dependent vasorelaxation in porcine coronary rings at concentrations ranging from 0.1 to 100 μg/mL. This effect was significantly inhibited by the NOS inhibitor L-NAME. Interestingly, the nonsteroidal anti-inflammatory drug indomethacin did not substantially affect the relaxation induced by *G. pentaphyllum*, suggesting that the mechanism of action may not involve cyclooxygenase pathways (Tanner et al., 1999).

The cardioprotective effects of total flavonoids from Jinhe yangxin in rats with isoproterenol-induced myocardial ischemia were marked by a reduction in ST elevation and lower serum levels of MDA, creatine kinase, cardiac troponin I, LDH, IL-6, and TNF-α. Additionally, there was an increase in serum activities of NO, SOD, and glutathione peroxidase (GSH-Px). Furthermore, these flavonoids were found to inhibit myocardial apoptosis and elevate the levels of phosphorylated Akt, glycogen synthase kinase 3 beta (GSK-3β), and eNOS proteins (Cheng et al., 2018).

In a study involving ovariectomized rats, supplementation with the ethanolic extract of *Ligusticum chuanxiong* Hort. [Apiaceae] significantly reduced weight gain, improved lipid profiles by lowering LDL cholesterol while increasing HDL cholesterol, and provided vascular protection. The beneficial effects are attributed to the extract’s antioxidant properties and its ability to enhance hepatic antioxidant enzyme levels and upregulate eNOS mRNA expression (Li et al., 2013).

Investigation of the effects of hydroalcoholic leaf extract of *Mandevilla moricandiana* (A.DC.) Woodson [Apocynaceae], on vasodilation, highlighted the role of the activation of the NO/cGMP pathway (Ferreira et al., 2017).

Huang et al. explored the cardioprotective effects of flavonoids isolated from *Millettia pulchra* (Voigt) Kurz [Fabaceae] using *in vitro* hypoxia/reoxygenation and *in vivo* rat myocardial ischemia/reperfusion models. They showed that pretreatment with these flavonoids reduced LDH and iNOS activities while increasing the activities of constitutive NOS and other ATPases. *In vivo*, *M. pulchra* flavonoids significantly improved hemodynamics, reduced oxidative damage, and decreased cardiocyte apoptosis (Bax/Bcl-2) (Huang et al., 2015). Yulangsan flavonoid is the main component derived from the plant *M. pulchra*. The effects of Yulangsan flavonoid on myocardial ischemia/reperfusion injury in rats included reduced infarct size and decreased levels of iNOS and caspase-3, as well as enhanced total and constitutive NOS activities. Additionally, it alleviated cellular edema and inflammatory cell infiltration (Zhang et al., 2014).

In studies conducted on hypertensive rats treated with *Morus alba* L. [Moraceae], it was found to have antihypertensive effects by increasing NO activity, thereby improving vascular reactivity and reducing vasoconstriction (Aghdam et al., 2024).

The essential oil of *Nardostachys jatamansi* (D.Don) DC. [Caprifoliaceae] was studied for its vascular benefits, particularly its effects on vasodilation in rat aortic rings and NO release in HUVECs. The findings revealed a rapid increase in NO release and eNOS phosphorylation, which L-NAME significantly inhibited. Additionally, treatment with the essential oil enhanced Akt kinase phosphorylation, with partial inhibition observed using the PI3K/Akt inhibitor (LY294002), indicating the involvement of the PI3K/Akt pathway. The essential oil also elevated intracellular calcium levels, and the calcium antagonist BAPTA-AM diminished both the increase in calcium levels and the phosphorylation of eNOS, as well as NO production (Maiwulanjiang et al., 2015).

Over 16 weeks, SHRs fed an oat protein diet exhibited significant reductions in both systolic and diastolic blood pressure, along with improvements in cardiac function and oxidative stress markers, notably increased NO levels and decreased TNF-α levels. The high antioxidant activity of oat protein and its ability to inhibit angiotensin-converting enzyme were also highlighted (Raj et al., 2024).

Treatment of HUVECs and EA. hy926 cells with alcohol-free red wine polyphenol extract at concentrations of 100–600 μg/mL resulted in a significant dose-dependent increase in NO release and eNOS protein levels, along with enhanced eNOS promoter activity (Leikert et al., 2002). A rat study indicated that a diet rich in de-alcoholized red wine significantly ACh-induced vasorelaxation compared to a control diet, highlighting the role of the NO/cGMP pathway in vascular health. Additionally, diets supplemented with quercetin or catechin enhanced endothelial function without increasing superoxide anion production, suggesting a protective mechanism via increased NO bioavailability. The expression of eNOS remained consistent across dietary groups (Benito et al., 2002). Short-term administration of red wine polyphenols (20 mg/kg/day for 7 days) in rats improved cardiac function and reduced ischemia-reperfusion injury. It lowered baseline pressure, increased heart rate, decreased infarct size, and reduced oxidative stress, without affecting post-ischemic contractile dysfunction. These effects were sensitive to NO synthase inhibition, suggesting a NO-dependent mechanism (Ralay Ranaivo et al., 2004).

Investigation of the effects of rice bran *Oryza sativa* L. [Poaceae] phenolic extracts on HUVECs under oxidative stress showed that these extracts modulated the expression of genes related to antioxidant and anti-inflammatory pathways, especially reducing ICAM1, CD39, CD73, and NADPH oxidase 4 (NOX4), and upregulating Nrf2, NAD(P)H quinone dehydrogenase 1 (NQO1), HO-1, and eNOS (Saji et al., 2019). Also, Red rice bran extract exhibited a potent endothelium-dependent vasorelaxant effect via activation of eNOS and subsequent NO production. This process is mediated through the PI3K/eNOS signaling pathway, which is crucial for vascular relaxation. In addition, this extract’s high taxifolin content, a flavonol, contributes to its vasorelaxant properties, distinguishing it from other rice bran extracts (Seong et al., 2022).

Isoflavones, primarily found in soy foods, enhance NO production, which is beneficial for cardiovascular health. A study on fermented soy extract demonstrated that it efficiently converts polyphenol glucosides to aglycones, and unlike unfermented extracts, significantly increases NO and prostaglandin E2 production in human endothelial cells. These findings suggest that aglycone-rich soy extracts could be promising therapeutic agents against atherosclerosis (De Andrade et al., 2015).

*Parkia speciosa* Hassk. [Fabaceae], commonly known as the stink bean, is prevalent in Southeast Asia and has been used in traditional medicine to treat hypertension. The effects of the methanolic extract from the empty pods of the plant on hypertension induced by L-NAME in rats were tested. The results indicated that administration of *Parkia speciosa* extract increased plasma NO levels and decreased systolic blood pressure, along with reduced angiotensin-converting enzyme and NADPH oxidase activities, and lipid peroxidation in the heart (Kamisah et al., 2017). Siti et al. reported that extracts from the empty pods of *P. speciosa* provide protective effects against angiotensin II-induced cardiomyocyte hypertrophy. This protective mechanism may involve the modulation of the angiotensin II/ROS/NO axis and the MAPK signaling pathway (Siti et al., 2021a).

A 28-day study of an aqueous extract of *Piper sarmentosum* Roxb. [Piperaceae] (Kadukmy™) in SHR showed that Kadukmy™ significantly reduced systolic and diastolic blood pressure, increased serum NO levels, decreased MDA levels, and reduced total cholesterol (Mohd Zainudin et al., 2015). Treatment with the n-hexane fraction of the seed of *Piper guineense* Schumach. and Thonn. [Piperaceae], another species of the *Piper* genus, extract at doses of 200 and 400 mg/kg/day significantly reduced systolic and diastolic blood pressure, increased NO concentration, and decreased activities of angiotensin-converting enzyme and arginase, as well as MDA levels and cardiac biomarkers (Olopade et al., 2024).

The results from both *in vitro* and *in vivo* studies indicate that the crude hydroalcoholic extract of *Polygala paniculata* L. [Polygalaceae] exhibits strong vasodilatory and antihypertensive effects, primarily mediated through the NO pathway, and this effect is potentially linked to its rutin content. In aortic rings with intact endothelium, the hydroalcoholic extract exhibited a significant vasodilatory effect at concentrations ranging from 30 to 1000 μg/mL, which was completely inhibited by soluble NOS and GC inhibitors. Furthermore, oral administration of the extract (30–300 mg/kg) demonstrated a dose-dependent antihypertensive effect in rats, which was notably diminished when L-NAME or methylene blue was injected (Lapa et al., 2011).

A study was conducted to evaluate the effects of pomegranate extract (Pomanox®) on coronary function in pigs fed either a normocholesterolemic or a hypercholesterolemic diet. Over 10 days, half of the pigs were administered 625 mg/day of Pomanox®, which contains 200 mg of punicalagins. The results demonstrated that Pomanox® supplementation significantly improved endothelial relaxation in dyslipidemic pigs, effectively aligning their vascular responses with those observed in normocholesterolemic pigs. These beneficial effects were associated with the activation of the Akt/eNOS signaling pathway and a reduction in markers of oxidative stress. Furthermore, Pomanox® enhanced lipoprotein resistance to oxidation and reduced DNA damage in coronary arteries (Vilahur et al., 2015). In another study, 24 major compounds from pomegranate were tested for their ability to inhibit ACE, and pedunculagin, punicalin, and gallagic acid were found to be the most effective inhibitors. Molecular docking studies indicated that these compounds inhibit ACE by forming hydrogen bonds and hydrophobic interactions with its catalytic residues and zinc ions. Additionally, pedunculagin was found to stimulate NO production and enhance eNOS activity, resulting in a dose-dependent increase in eNOS protein expression (Ali et al., 2023).

Pumpkin seed oil demonstrated antihypertensive and cardioprotective effects, particularly in L-NAME-induced hypertension in rats. Doses of pumpkin seed oil at 40 or 100 mg/kg were administered orally for 6 weeks, resulting in a significant reduction in blood pressure. The mechanism involves NO generation, as evidenced by restored levels of NO metabolites and decreased MDA levels (El-Mosallamy et al., 2012).

*Rheum officinale* Baill [Polygonaceae] is a traditional Chinese drug that has been able to improve blood circulation and treat thrombosis by reducing ROS. Key active ingredients included emodin, aloe-emodin, and physcion, which target the NOS2 and NOS3 enzymes. By positively regulating NOS3 mRNA expression and altering the levels of arginine, glutamate, and glutamine, it improved thrombosis inhibition (Zhang et al., 2022).

The ethanolic extract from the fruits of the *Sarcopoterium spinosum* (L.) Spach [Rosaceae] was assessed for its antioxidant and cytoprotective effects in a model of endothelial dysfunction utilizing HECV cells. Treatment with 10 μg/mL of this extract, as well as its polyphenolic constituents—corilagin and quercetin—either before or following exposure to 30 µM hydrogen peroxide, resulted in a significant decrease in ROS production and lipid peroxidation. Furthermore, this treatment effectively enhanced glutathione levels, restored NO concentrations, and mitigated protein denaturation (Zbeeb et al., 2024).

A study investigated the vasodilatory effects of hexane extracts from *Schisandra chinensis* on the rat thoracic aorta. The results demonstrated that extracts induced a concentration-dependent relaxation in aortas. This relaxation was significantly inhibited by L-NAME and ODQ, indicating the involvement of the NO and guanyl cyclase signaling pathways. Additionally, these extracts activated eNOS and produced nitrites, further supporting the NO-mediated mechanism (Park et al., 2009).

*Senecio serratuloides* DC [Asteraceae] has been traditionally used to treat hypertension and various skin conditions. Antihypertensive effects of its hydroethanolic extract in rats with L-NAME-induced hypertension. The extract significantly and dose-dependently lowered both systolic and diastolic blood pressure, improved lipid profiles, prevented reductions in serum levels of angiotensin II and NO, and decreased collagen deposition in cardiac tissue (Tata et al., 2019).

The aqueous extract of *Sesamum radiatum* Schum. and Thonn. [Pedaliaceae] demonstrated notable vasorelaxant effects, primarily through the NO pathway. In studies conducted with guinea pig aortic preparations, this extract induced relaxation in a concentration-dependent manner. The relaxation response was significantly reduced in preparations and endothelium-denuded preparations, and was further inhibited by NOS inhibitors. Additionally, the contractile effects of the extract appear to be mediated by cyclooxygenase activation and by potassium channel involvement (Konan et al., 2008).

In traditional Chinese medicine, the root of *Sophora flavescens* Aiton [Fabaceae], known as Ku Shen, is highly valued for its medicinal properties (He et al., 2015). Jin et al. reported that the ethanolic extract of *S. flavescens* roots (concentrations ranging from 0.1 to 100 μg/mL) induces relaxation of vascular smooth muscle through an endothelium-dependent signaling pathway involving NO/sGC/cGMP signaling (Jin et al., 2011).

*Terminalia fagifolia* Mart. [Combretaceae] is a plant indigenous to the Brazilian cerrado. Research on its ethanolic extract and different partition fractions has shown that they possess vasorelaxant properties in rat thoracic aorta rings, which were mediated by the NO/sGC/cGMP pathway and potassium channels (De Carvalho et al., 2019).

The crude extract of *Terminalia pendula* var. *pendula* (syn. *Anogeissus acuminata*) [Combretaceae] demonstrated *in vitro* cardio-relaxant and vasorelaxant effects, as well as a significant reduction in blood pressure *in vivo*. It positively affected left ventricular hypertrophy in rats, as evidenced by decreased cardiac cell size, the absence of inflammatory cells, lower levels of ACE and renin, and increased levels of NO and cGMP. In an Acute Myocardial Infarction model, the extract reduced markers of myocardial injury, including creatine kinase, creatine kinase-MB, and LDH, and decreased necrosis and inflammation (Saqib et al., 2020).

*Urtica dioica* L. [Urticaceae], commonly known as nettle, exhibits antihypertensive effects primarily through its vasodilatory mechanisms. L-NAME and ODQ significantly inhibit the vasodilation induced by nettle extracts in this process. Additionally, the presence of potassium channel blockers attenuates the vasodilatory effects of U. dioica extracts, suggesting that potassium channel activation also plays a key role (Testai et al., 2002).

Thaci et al. investigated the effects of *V. agnus-castus* L. [Lamiaceae], commonly known as chaste tree, extract on isolated rabbit arterial rings. This extract (0.15–0.75 mg/mL) induced concentration-dependent relaxation in precontracted aortic rings, indicating potential calcium channel-blocking activities. The relaxation effect was found to be endothelium-dependent, as evidenced by the effects of inhibitors such as L-NAME and indomethacin, which reduced the extract’s impact. Conversely, bradykinin and zaprinast enhanced the relaxant effects. The findings suggested that *Vitex agnus-castus* extract-induced relaxation was mediated by NO/cGMP and prostaglandin synthesis (Thaçi et al., 2022).

Dongmo et al. evaluated the vasorelaxant activity of extracts of *Vitex doniana* Sweet (syn. *Vitex cienkowskii*) [Lamiaceae] and isolated compounds using rat aortic rings. The extract demonstrated significant relaxation effects in a concentration- and endothelium-dependent manner, with an effective concentration of 12.12 μg/L. The relaxation was inhibited by L-NAME and ODQ, indicating that the mechanism likely involves NO release and sGC activation, which produce cGMP as a second messenger. Additionally, the compounds salvin A and maslinic acid, isolated from this plant, reduced mean arterial pressure without affecting heart rate and exhibited vigorous antioxidant activity in the 2,2-diphenyl-1-picrylhydrazyl (DPPH) assay (Dongmo et al., 2011).

Phenolic-rich extracts from Mediterranean wild plants such as wild artichoke (*Cynara cardunculus* L. [Asteraceae]) and thyme (*Thymus pulegioides* L. [Lamiaceae]) significantly enhance NO release in porcine aortic endothelial cells and brain cell membranes (Grande et al., 2004).

The findings of a rat study demonstrated that both the aqueous and ethyl acetate fractions of *Ziziphus jujuba* Mill. [Rhamnaceae] could inhibit the development of hypertension induced by L-NAME by modulating the NO system (Mohebbati et al., 2020).

The antihypertensive effects of the aqueous methanolic extract of *Ziziphus oxyphylla* Edgew. [Rhamnaceae] were investigated in rats with L-NAME-induced hypertension. Phytochemical analysis revealed the presence of key compounds, including kaempferol and quercetin, which enhanced NO production. Chronic administration of this methanolic extract resulted in a significant reduction in blood pressure and an increase in serum NO and cGMP levels. Furthermore, the extract downregulated pro-inflammatory markers, such as IL-6 and TNFα, and angiotensin-converting enzymes, while simultaneously upregulating eNOS (Shaukat et al., 2022).

#### 3.1.3 Clinical aspects of targeting the L-arginine/NO/cGMP/K_ATP_ pathway for the treatment of cardiovascular diseases

Various clinical studies have reported evaluating targeting the L-arginine/NO/cGMP/K_ATP_ pathway for the treatment of cardiovascular diseases (Table 3).

**TABLE 3:** Clinical studies of phytochemicals and phytoextracts modulating the L-arginine/NO/cGMP/K_ATP_ pathway in cardiovascular disease.

Compound/Extract	Type/Class	Method	*In vitro*/*in vivo/clinical* models	Mechanism and outcome	References
Black soybean extract	Poasted black soybeans	Vascular function and oxidative stress reduction	Clinical trial: 24 healthy volunteers	↑Vascular function, ↑eNOS mRNA, ↑NO metabolites, ↓Oxidative stress	Yamashita et al. (2020), Akagi et al. (2024)
Beetroot juice	Juice	Hypertension study	Clinical trial: 15 hypertensive adults	↑NO availability ↓Blood pressure, ↑Redox status, ↓Classical monocytes	Fejes et al. (2024)
Cocoa	Diet	Cardiovascular biomarkers	Clinical trial: Randomized, parallel-group study, 60 healthy adults	Positive modulation of microbiota metabolism ↑FMD values ↓TMAO, ↓Uric acid levels	García-Cordero et al. (2023)
Dark chocolate	Diet	Blood pressure reduction and vascular function	Clinical trial: 44 hypertensive and 21 healthy adults	↑NO, ↑Endothelial function ↓Blood pressure	Engler et al. (2004), Taubert et al. (2007)
*Eleutherococcus senticosus* (Rupr. and maxim.) maxim. (syn. *Acanthopanax senticosus* Harms)	Aqueous extract of fruits	Vascular function	Clinical trial: A 12-week randomized, placebo-controlled trial in 76 healthy adults with at least two of borderline high blood pressure, smoking, or borderline blood lipids	eNOS activation ↓Blood pressure	Oh et al. (2020)
Epicathechin and papple puree	Supplemnetation	NO bioavailability	Clinical trial: Randomized, placebo-controlled, crossover trial	↑NO bioavailability	Hollands et al. (2013)
Fitnox®	Supplementation	Athletic performance and NO enhancement	Clinical trial: Double-blind, placebo-controlled	↑Nitrate and nitrite levels, ↑NO	Jacob et al. (2018)
Grape juice	Supplementation	Cardiovascular function and platelet function	Clinical trial: 20 healthy subjects	↑NO, ↑Endothelial function, ↑platelet function, ↓Superoxide	Freedman et al. (2001)
Hesperidin	Flavonoid	Endothelial function improvement	*In vitro*: Bovine aortic endothelial cells	↑p-eNOS, ↑NO, ↓Adhesion molecule expression	Rizza et al. (2011)
High-flavonoid diet	Diet	Cardiovascular risk reduction	Clinical trial: 174 participants	↑NO, ↑Microvascular reactivity, ↓Inflammatory markers	Macready et al. (2014)
High-flavonoid diet	Apple and spinach	Endothelial function and blood pressure	Clinical trial: Randomized controlled trial in healthy adults	↑Endothelial function ↓Blood pressure	Bondonno et al. (2012)
Green tea	Extract	Cardiovascular effect	Clinical trial: 20 healthy non-smoking females, 4-week controlled intervention study	↓MDA	Freese et al. (1999)
Mediterranean diet	Diet	Blood pressure reduction study	Clinical trial: 200 high-risk participants	↑Urinary TPE, ↑NO ↓Blood pressure	Medina-Remón et al. (2015)
Plant based bioequivalent nitrate complex	Phytophenol rich food extracts	NO bioavailability	Clinical trial: A randomized, double-blind, placebo-controlled trial of 67 hypertensive individuals	↑Endothelial function ↓Blood pressure	Cherukuri et al. (2020)
*Terminalia chebula* Retz	Aqueous extract	Endothelial function	Clinical trial: A randomized double-blind, placebo-controlled study, Type 2 diabetes mellitus	No significant changes in vital, hematological, renal, and hepatic functions ↑NO ↑HDL-C ↓MDA, Inflammation ↓marker, ↓LDL-C, ↓VLDL-C	Pingali et al. (2020)
Watermelon Juice	Citrulline	NO bioavailability	Clinical trial: Controlled dietary intervention	↑Arginine, ↑NO, ↓Blood pressure	Collins et al. (2007)
Wild blueberry	Juice	Endothelial function	Clinical trial: 76 participants	↑NO, ↑Vascular function, ↓Systemic arterial stiffness	Johnson et al. (2015), Curtis et al. (2019)
Wine	Diet	Blood pressure	Clinical trial: Randomized, crossover clinical trial, 67 men at high cardiovascular risk	↑NO ↓Blood pressure	Chiva-Blanch et al. (2012)
	Diet	Blood pressure	Clinical trial: Men at high cardiovascular risk	↑NO ↓Blood pressure No changes in food intake, physical activity, or body weight	Roth et al. (2019)

6-keto-PGF1α, 6-Keto-Prostaglandin F1 alpha; AC/cAMP/PKA, Adenylate Cyclase/Cyclic AMP/Protein Kinase A; ACE, Angiotensin-Converting Enzyme; ACh, Acetylcholine; AhR-RGE, Aryl Hydrocarbon Receptor-Regulated Gene Enhancement; Ahr-TN, Aryl Hydrocarbon Receptor-Translocated; AI, atherogenic index; Akt, Protein Kinase B ALDH, aldehyde dehydrogenase; AMPK, AMP-Activated Protein Kinase; Ang II, Angiotensin II; Ang II-CFP, Angiotensin II; Central Fusion Point; AP-1, Activator Protein 1; ARNT, aryl hydrocarbon receptor nuclear translocator; AS, atherosclerosis; ATPases, Adenosine Triphosphatases; Bcl-2/Bax, B-cell Lymphoma 2/Bcl-2-associated X Protein; bFGF, basic fibroblast growth factor; BMI, body mass index; BP, blood pressure; BRS, baroreflex sensitivity; CaMKII, Calcium/Calmodulin-Dependent Protein Kinase II; CaMKKβ, Calcium/Calmodulin-Dependent Protein Kinase Kinase Beta CAT, catalase; CD39, Cluster of Differentiation 39; CD73, Cluster of Differentiation 73; CDR, cognitive decline rate; cGMP, cyclic guanosine monophosphate; CK, creatine kinase; CLS, Capillary-like Structures; cNOS, constitutive nitric oxide synthase; CPK, creatine phosphokinase; CS, Cholesterol SulfatecTnI, Cardiac Troponin I; CVP, central venous pressure; CYP7A1, Cholesterol 7 Alpha-Hydroxylase; ECFC, Endothelial Colony-Forming Cells; EDHF, Endothelium-Derived Hyperpolarizing Factor; EDR, Endothelium-Dependent Relaxation; EDV, end diastolic volume; eNO, endothelial nitric oxide; eNOS, endothelial nitric oxide synthase; ERK1/2, Extracellular Signal-Regulated Kinases 1/2,ET-1, Endothelin-1; GC, guanylate cyclase; GSH/GSSG, Reduced Glutathione/Glutathione Disulfide Ratio; GSH-Px, Glutathione Peroxidase; GSK-3β, Glycogen Synthase Kinase 3 Beta; H_2_O_2_, hydrogen peroxide; Hcy-ED, Homocysteine-Endothelial Dysfunction; HDL, High-Density Lipoprotein; HDL-C, High-Density Lipoprotein Cholesterol; HO-1, Heme Oxygenase 1; ICAM-1, Intercellular Adhesion Molecule 1; IL-1β, Interleukin 1 Beta; IL-6, Interleukin 6; iNOS, inducible nitric oxide synthase; IP3R, Inositol 1,4,5-Trisphosphate Receptor,JNK, c-Jun N-terminal kinase,JNK1/2, c-Jun N-terminal kinases 1/2; K_ATP_, ATP-Sensitive Potassium Channel; KCM, potassium channel modulation; KDR, kinase insert domain receptor; K-Ras, Kirsten Rat Sarcoma Viral Oncogene Homolog; KV; and K_ATP_, Voltage-Gated Potassium and ATP-Sensitive Potassium Channels; LDH, lactate dehydrogenase; LDL, Low-Density Lipoprotein; LDL-C, Low-Density Lipoprotein Cholesterol; LF and HF, low frequency and high frequency components of power index; LPO, lipid peroxidation; MAP, mean arterial pressure; MAPK, Mitogen-Activated Protein Kinase; MDA, Malondialdehyde,MF, myocardial fibrosis; mitoK_ATP_, Mitochondrial ATP-Sensitive Potassium Channel; MMP, matrix metalloproteinase; mRNA, messenger ribonucleic acid; NADPH, nicotinamide adenine dinucleotide phosphate; NLRP3, NLR; Family Pyrin Domain Containing 3; NO, Nitric Oxide,NOS2 and NOS3, Nitric Oxide Synthase 2 and 3; NOX4, NADPH; Oxidase 4; NQO1, NAD(P)H Quinone Dehydrogenase 1; NRC, nuclear regulatory commission; Nrf2, Nuclear Factor Erythroid 2–Related Factor 2; OS, oxidative stress; p38 MAPK, p38 Mitogen-Activated Protein Kinases; p47phox, Phox Subunit of NADPH; oxidase; PE, phenylephrine; PGI, prostacyclin; PI3K, Phosphoinositide 3-Kinase,p-NF-κB, Phosphorylated Nuclear Factor kappa B,PTEN, phosphatase and tensin homolog; RAS, Renin-Angiotensin System; ROS, reactive oxygen species; RS, renal stenosis; SAL, salinity; sGC, soluble guanylate cyclase; SIPS, Stress-Induced Premature Senescence; Smad2/3, SMAD; Family Member 2/3; SOD, superoxide dismutase; STCL, short term cellular leukemia; TG, Triglycerides,TGF-β1, Transforming Growth Factor Beta 1; TNFR, Tumor Necrosis Factor Receptor,TNF-α, tumor necrosis factor alpha; TPE, total protein excretion; TRPA1, Transient Receptor Potential Ankyrin 1; TXB2, Thromboxane B2; VCAM-1, Vascular Cell Adhesion Molecule 1; VD, vascular diameter; VEGF, vascular endothelial growth factor; VOCC, Voltage-Operated Calcium Channels; VP, venous pressure; VR, vascular resistance; β2-AR, Beta-2; adrenergic receptor; β-Gal, Beta-Galactosidase.

Black soybeans are rich in polyphenols, such as isoflavones and anthocyanidins (Ganesan and Xu, 2017). A clinical study involving 24 healthy volunteers found that daily supplementation with 100 mg of black soybean seed coat extract for 2 weeks led to significant improvements in vascular function compared with a placebo group. Findings indicated that treatment with this extract elevated eNOS mRNA and NO metabolite levels, while reducing markers of oxidative stress. Specifically, it inhibited markers of cellular senescence and decreased intracellular ROS levels (Akagi et al., 2024). A separate study in healthy women showed that an 8-week diet including black soybeans reduced vascular stiffness, lowered markers of oxidative stress, and increased NO levels. Among the 44 participants, 33 showed signs of improved vascular age. Additionally, the consumption of black soybeans led to higher levels of 12 polyphenols in both plasma and urine. Therefore, black soybeans appear to promote vascular function by increasing NO levels and reducing oxidative stress through their polyphenol content (Yamashita et al., 2020).

In a randomized, placebo-controlled, crossover study involving 15 hypertensive adults, researchers assessed the effects of dietary inorganic nitrate on oxidative stress and inflammation. Participants consumed a single dose of approximately 400 mg of nitrate and daily doses of nitrate-rich beetroot juice over 4 weeks. The results showed a significant decrease in the oxidized LDL/NO ratio and an increase in the GSH/oxidized glutathione (GSSG) ratio after 4 weeks, indicating improved redox status from dietary nitrate intake. Additionally, there was a notable reduction in classical monocytes following the nitrate intervention. These findings suggested that regular consumption of inorganic nitrate may help shift the redox balance to a less pro-oxidative state, potentially benefiting cardiovascular health (Fejes et al., 2024).

A randomized trial with 60 healthy participants revealed that cocoa consumption significantly reduced serum levels of trimethylamine N-oxide (TMAO) and uric acid, while enhancing flow-mediated vasodilation. This improvement in vascular function was associated with increased carbohydrate fermentation and was negatively correlated with TMAO levels, suggesting a potential link between cocoa flavanols and modulation of the beneficial microbiota. Additionally, the findings suggested that regular intake of these compounds could lead to lower blood pressure and improved lipid profiles, particularly highlighting the cardiovascular benefits of cocoa flavanols through mechanisms such as enhanced NO availability and improved endothelial function (García-Cordero et al., 2023).

A randomized controlled trial involving 44 adults with untreated hypertension assessed the impact of a daily intake of 6.3 g of dark chocolate containing 30 mg of polyphenols over 18 weeks. The primary outcome was a change in blood pressure, while secondary measures included plasma markers of NO and oxidative stress. Results indicated a significant reduction in systolic and diastolic blood pressure, alongside a decrease in hypertension prevalence (Taubert et al., 2007). A double-blind study of 21 healthy adults found that high-flavonoid dark chocolate improved brachial artery dilation compared to low-flavonoid chocolate, with increased plasma epicatechin levels in the high-flavonoid group. However, there were no significant differences in LDL oxidation, blood pressure, or lipid profiles between the two groups. The results suggest that flavonoid-rich dark chocolate may improve endothelial function without affecting other cardiovascular risk factors (Engler et al., 2004).

A randomized, double-blind, placebo-controlled trial was conducted over 12 weeks, with 76 participants assigned to receive either placebo or *Eleutherococcus senticosus* (Rupr. and Maxim.) Maxim. (syn. *Acanthopanax senticosus* Harms) [Araliaceae] fruit at two different doses (500 mg/day and 1000 mg/day). The results showed that the lower dose led to significant improvements in systolic blood pressure and arterial stiffness, along with increased endothelial function, as indicated by enhanced eNOS phosphorylation (Oh et al., 2020).

In a randomized, placebo-controlled crossover trial, the effects of epicatechin from apple extract (70 and 140 mg) and apple puree (70 mg) on plasma and urinary NO metabolites were evaluated. The epicatechin drinks showed significantly higher plasma concentrations and absorption than the puree, with the 140 mg drink demonstrating a more than two-fold increase in bioavailability. All products improved urinary excretion of NO metabolites compared to placebo, particularly the high-dose drink. These results indicate that higher doses of epicatechin may enhance bioavailability and support cardiovascular health by increasing NO availability (Hollands et al., 2013).

The sports nutritional supplement Fitnox®, formulated with a blend of *Kaempferia parviflora* Wall. ex Baker [Zingiberaceae], pomegranate peel polyphenols, and saponins of *Moringa oleifera* Lam. [Moringaceae] was evaluated in a double-blind, placebo-controlled trial. Results revealed that a single 250 mg dose significantly increased nitrate and nitrite levels in both blood serum and saliva within 24 h compared to the placebo group. Additionally, key pharmacokinetic parameters, such as maximum serum concentrations and time to peak concentration, were markedly improved. Notably, the elevated NO levels persisted for at least 12 h following administration. These findings suggest that Fitnox® may enhance athletic performance and physical endurance by boosting NO production, positioning it as a promising natural option for athletes seeking to improve cardiovascular fitness and endurance (Jacob et al., 2018).

In a study involving 24 healthy volunteers, researchers assessed the effects of a fruit and vegetable puree-based drink (FVPD) on vasodilation and antioxidant status, with a focus on a specific genetic polymorphism. The findings revealed that individuals with the GG genotype experienced significant enhancements in endothelium-dependent vasodilation and reduced LDL oxidation following FVPD consumption. In contrast, those with the GT genotype showed no significant changes. This suggests that the eNOS Glu298Asp genotype plays a crucial role in determining how individuals respond to fruit and vegetable intake with respect to vascular function and oxidative stress (George et al., 2012).

A clinical study suggests that hesperidin may contribute to the cardiovascular health benefits associated with consuming citrus fruits. When administered orally at 500 mg daily for 3 weeks, hesperidin significantly enhanced endothelial function in individuals with metabolic syndrome, as evidenced by improved flow-mediated dilation. Additionally, there were reductions in inflammatory biomarkers, such as high-sensitivity C-reactive protein and soluble E-selectin (Rizza et al., 2011).

A clinical study investigated the effects of high-flavonoid and low-flavonoid diets on vascular function and cardiovascular disease risk in 174 participants at increased risk. Results showed that a high-flavonoid diet improved microvascular reactivity, reduced inflammatory markers, and increased plasma NO levels, which are vital for vascular health (Macready et al., 2014).

The effects of flavonoid-rich apples and nitrate-rich spinach on NO status, endothelial function, and blood pressure were assessed in 30 healthy participants. Each treatment (apple, spinach, and apple + spinach) was administered at a dose of 200 g, resulting in significant increases in plasma S-nitrosothiol and nitrite levels. All treatments improved flow-mediated dilatation and reduced pulse pressure, apple and spinach lowering systolic blood pressure. However, the combination of apple and spinach did not yield additive effects on NO status or blood pressure (Bondonno et al., 2012).

In a substudy of the PREDIMED trial involving 200 participants at high cardiovascular risk, those following Mediterranean diets supplemented with either extra-virgin olive oil or nuts showed significant reductions in systolic and diastolic blood pressure after 1 year. The dietary interventions led to increased total polyphenol excretion in the urine and elevated plasma NO levels, indicating enhanced endothelial function (Medina-Remón et al., 2015).

A study found that after 14 days of purple grape juice supplementation, participants showed significant improvements in platelet function, with NO levels increasing and superoxide production decreasing. These effects are attributed to the flavonoids in grapes, suggesting a potential mechanism for their protective role against cardiovascular disease independent of alcohol consumption (Freedman et al., 2001).

The effectiveness of a plant-based nitrate complex in enhancing NO bioavailability and its impact on lowering blood pressure were evaluated in 67 participants with hypertension. Over 12 weeks, those who received the nitrate supplementation experienced significant reductions in both systolic and diastolic blood pressure, as well as improvements in endothelial function (Cherukuri et al., 2020).

A clinical study evaluated the effects of aqueous extract of *Terminalia chebula* Retz. [Combretaceae] at 250 mg and 500 mg doses on endothelial function and oxidative stress biomarkers over 12 weeks. Results indicated that both doses significantly improved endothelial function. Additionally, aqueous extract treatment led to notable improvements in cardiovascular risk indicators, including NO levels and markers of oxidative stress, such as MDA and glutathione (Pingali et al., 2020).

In a clinical study, participants consumed controlled diets along with varying amounts of watermelon juice over 3 weeks, revealing a 12% increase in plasma arginine with lower doses and a 22% increase with higher doses. Additionally, ornithine levels rose by 18% after the higher-dose treatment, while citrulline levels remained stable. These findings suggest that citrulline from watermelon is effectively converted to arginine, indicating the potential for watermelon juice to enhance arginine availability in the body (Collins et al., 2007).

In a single-blind, placebo-controlled trial, 19 women aged 39-64 consumed either 240 mL of wild blueberry juice or a placebo for 7 days. Notably, serum nitrates and nitrites, indicators of NO production, increased significantly after blueberry juice consumption (Stote et al., 2017). Also, results of a randomized, double-blind, placebo-controlled trial showed that 8 weeks of blueberry consumption increased NO levels and improved vascular function (Johnson et al., 2015). A double-blind, randomized controlled trial investigated the effects of 6 months of blueberry intake on insulin resistance and cardiometabolic function in individuals with metabolic syndrome. Participants who consumed 1 cup of blueberries daily experienced significant improvements in endothelial function, reduced systemic arterial stiffness, and decreased cGMP concentrations. In participants who were not using statins, there were notable increases in HDL cholesterol, HDL particle density, and apolipoprotein A-I levels following the 1-cup daily intervention. However, insulin resistance, pulse wave velocity, blood pressure, NO, and overall plasma thiol status remained unchanged (Curtis et al., 2019).

In another report, 67 participants underwent a crossover clinical trial, consuming either 30 g of alcohol from red wine, dealcoholized red wine, or gin over 4-week periods. Results demonstrated significant reductions in both systolic and diastolic blood pressure following the intervention with dealcoholized red wine, which correlated with increased plasma NO levels (Chiva-Blanch et al., 2012). Additionally, a randomized, crossover-controlled trial involving 38 men demonstrated that daily consumption of 30 g of a polyphenol-rich Andalusian white wine significantly reduced systolic and diastolic blood pressure while increasing plasma NO levels (Roth et al., 2019). After 15 days of controlled wine consumption, significant increases in plasma resveratrol levels and stimulated platelet NO release were observed. Mechanistically, resveratrol enhances platelet NOS activity and promotes Akt phosphorylation, thereby activating eNOS. Furthermore, it reduces p38 MAPK phosphorylation, a pro-inflammatory pathway, and decreases NADPH oxidase activity, thereby reducing ROS production (Gresele et al., 2008). A study investigated the effects of resveratrol on relaxation in human coronary bypass grafts, specifically the saphenous vein and internal mammary artery. The results showed significant vasodilation in both vessels, primarily mediated by endothelium-dependent NO pathways (Rakici et al., 2005).

In a controlled study involving twenty healthy non-smoking females, the effects of a high linoleic acid diet combined with green tea extract on various cardiovascular risk markers were assessed over 4 weeks. The diet consisted of 27% fat, 14% protein, and 59% carbohydrates, with participants receiving either 3 g/day of encapsulated green tea extract or a placebo. Results indicated that while green tea extract significantly reduced plasma MDA, an indicator of lipid peroxidation, no significant changes were observed in serum lipids, NO metabolites, thromboxane production, or blood coagulation markers compared to the placebo group (Freese et al., 1999).

## 4 Phytochemicals and phytoextracts modulating the L-arginine/NO/cGMP/K_ATP_ pathway for the treatment of metabolic disorders

By improving endothelial function, increasing NO bioavailability, and exerting antioxidant effects, phytochemicals can provide a complementary approach to conventional therapies and ultimately help improve health outcomes in metabolic diseases (Figure 2).

### 4.1 Phytochemical

In T2DM, high glucose and fatty acids impair endothelial NO synthase phosphorylation, leading to decreased NO levels and increased ET-1 synthesis. This dysfunction is linked to elevated ROS production. However, hydroxytyrosol and polyphenol extracts from extra virgin olive oil can partially reverse these detrimental effects, restoring NO levels and reducing ET-1 synthesis (Storniolo et al., 2014).

Treatment with oligonol, a polyphenolic compound, counteracted these effects by enhancing eNOS phosphorylation and restoring NO synthesis. Specifically, oligonol treatment activates Akt and modulates the p38 MAPK pathway, both of which are crucial for eNOS function. The mechanism involves inhibiting protein kinase C (PKC) epsilon, which participates in dephosphorylation (Zhang et al., 2010).

Administration of resveratrol to diabetic rats resulted in reduced glucose levels and improved left ventricular function after ischemia-reperfusion. Mechanistically, resveratrol upregulated protective proteins, including Trx-1, NO/HO-1, and VEGF, while enhancing manganese superoxide dismutase (MnSOD) activity. Additionally, it decreased infarct size and cardiomyocyte apoptosis compared to untreated diabetic animals. Notably, the presence of L-NAME diminished the cardioprotective effects of resveratrol, indicating that NO signaling plays a crucial role in its mechanism of action (Thirunavukkarasu et al., 2007).

Boydens et al. demonstrated that 30 µM resveratrol and quercetin effectively protected mouse corpora cavernosa from damage induced by diabetes, specifically with regard to NO-mediated relaxant responses (Boydens et al., 2016). A study examined the antihypertensive effects of cultured red grape berry cells in rats with metabolic syndrome and HUVECs. The findings revealed that supplementation with red grape berry cells significantly reduced blood pressure, plasma triglycerides, and insulin levels, while improving endothelial function by decreasing ET-1 secretion and increasing eNOS expression. These positive effects are likely due to the high polyphenol and resveratrol content in grapes (Leibowitz et al., 2014). The investigation into the impact of alcohol-free Alibernet red wine extract on NOS activity and pro-inflammatory markers in metabolic syndrome revealed notable findings. Male Wistar Kyoto rats and SHR were administered alcohol-free Alibernet red wine extract over 3 weeks. The results indicated that although the extract did not significantly affect total NOS activity, it led to a marked reduction in NF-κB and iNOS protein expression in the hearts and aortas of hypertensive rats (Janega et al., 2014). In hypercholesterolemic rabbits, resveratrol and red wine improved endothelial function by mitigating the reduction in flow-mediated dilation associated with a high-cholesterol diet. This improvement was linked to a decrease in plasma ET-1 levels and an increase in NO concentrations (Zou et al., 2003).

A comparative study evaluated the effects of ferulic acid compared to the standard aldose reductase inhibitor zopolrestat in rats fed a fructose-rich diet. The findings indicated that ferulic acid played a protective role against metabolic syndrome by lowering serum insulin levels, triglycerides, and cholesterol, and by enhancing vascular reactivity and NO production in isolated aortic samples. After 12 weeks of treatment, both ferulic acid and zopolrestat effectively mitigated hyperinsulinemia and hypertension, successfully restoring normal endothelial relaxation responses (Badawy et al., 2013).

Geranyl acetate, a terpenoid compound, exhibited notable diuretic and anti-hyperuricemic effects in male rats, particularly at a dosage of 100 mg/kg. Molecular docking studies indicated a strong binding affinity between geranyl acetate and NOS. *In vitro* experiments demonstrated significant free radical scavenging activity and effective inhibition of key enzymes, including urease, xanthine oxidase, and acetylcholinesterase (Shah et al., 2024).

Nothofagin is a natural 3′-C-β-D-glucoside derived from phloretin. In perfused rat kidney experiments, nothofagin reduced perfusion pressure in a dose-dependent manner when the endothelium was intact. This effect was inhibited by endothelium removal or NOS blockade, indicating that NO plays a crucial role in the vasodilatory response. Furthermore, the involvement of calcium-activated high-conductance potassium channels was confirmed, as selective blockers of these channels abolished the vasodilatory effects of nothofagin (Marques et al., 2020).

Provinols is a polyphenolic extract derived from red wine. In a study with Zucker fatty (ZF) rats, supplementation with provinols over 8 weeks improved glucose metabolism by lowering plasma glucose and triglycerides while enhancing cardiac performance through increased left ventricular fractional shortening and cardiac output. The mechanism underlying these benefits involves correcting endothelial dysfunction in aortas, where provinol improves endothelium-dependent relaxation and enhances NO bioavailability by increasing eNOS activity and reducing superoxide anion release (Agouni et al., 2009). In another study, it was reported that in L-NAME-treated rats, 40 mg/kg of provinol significantly decreased myocardial fibrosis, enhanced the reduction in aortic cross-sectional area, improved endothelium-dependent relaxation, and reduced aortic contractility. These beneficial effects of provinol were also associated with increased NO synthase activity in both the left ventricle and the aorta (Bernátová et al., 2002).

In a study on hypertriacylglycerolemic rats, the pyridoindole derivative SMe1EC2 (10 mg/kg) and the natural polyphenol rutin (100 mg/kg) were found to upregulate eNOS3 expression, a crucial factor in NO production. While SMe1EC2 improved coronary flow and left ventricular function after ischemia, rutin decreased coronary flow. Notably, SMe1EC2 also reduced sensitivity to electrically induced ventricular fibrillation. These findings suggest that SMe1EC2 may be more effective than rutin in addressing cardiovascular disorders related to metabolic syndrome by enhancing NO signaling and mitigating inflammation (Salvaras et al., 2021).

Kidney failure is the leading cause of death in malignant hypertension. Resveratrol showed therapeutic potential in the kidney tissue by reducing oxidative damage, enhancing antioxidant defenses, and restoring the NO pathway in hypertensive rats (Grujić-Milanović et al., 2022). In a 12-week experimental study, rats fed with adenine developed hypertension and kidney dysfunction, conditions that were alleviated by supplementation with resveratrol butyrate monoester. Mechanistically, this compound enhanced NO bioavailability, rebalanced the renin-angiotensin system, decreased oxidative stress, and modified gut microbiota profiles (Tain et al., 2023).

In their study, Chtourou et al. examined the effects of a mixture of naringin, chlorogenic acid, and quercetin on renal fibrosis in STZ-induced diabetic rats. This supplementation effectively restored renal function and reversed pathological changes, including renal interstitial fibrosis, leukocyte infiltration, and glomerular degeneration. These protective effects were attributed to antioxidant mechanisms that mitigated oxidative stress, as well as the inhibition of key inflammatory mediators, including TGF-β, TNF-α, and p53. Additionally, it helped preserve sodium-potassium pump activity, further contributing to its renal protective effects (Chtourou et al., 2022) (Table 4).

**TABLE 4:** Phytochemicals modulating the L-arginine/NO/cGMP/K_ATP_ pathway in metabolic disorders.

Compound	Class	Methods	*In vitro*/*In vivo* models	Mechanisms and outcomes	References
Chlorogenic acid, Naringin, and Quercetin	Flavonoid	STZ-induced diabetic nephropathy	*In vivo:* Diabetic rat model	↑Renal function, ↓Fibrosis ↓Oxidative stress ↓TGF-β ↓TNF-α ↓p53 ↓Leukocyte infiltration, ↓Glomerular degeneration	Chtourou et al. (2022)
Ferulic acid	Phenolic Acid	Fructose-induced metabolic syndrome	*In vivo:* Fructose-fed rats	↑NO, ↑Vascular reactivity ↑Endothelial relaxation ↓Insulin, ↓Triglycerides, ↓Cholesterol ↓Hyperinsulinemia, ↓Hypertension	Mathew and Abraham (2006)
Ferulic acid and zopolrestat	Phenolic compound	Hypertension in diabetes	*In vivo:* Male rats	↑Endothelial relaxation, ↑NO	Badawy et al. (2013)
Geranyl acetate	Terpenoid	Hyperuricemia and oxidative stress	*In vivo:* Male rats	↑NOS, ↑Diuretic, ↑Antioxidant effects ↓Xanthine oxidase, ↓Urease ↓Acetylcholinesterase	Shah et al. (2024)
Hydroxytyrosol	Polyphenol	High glucose and fatty acid-induced endothelial dysfunction	*In vitro:* Endothelial cells	↑NO ↓Oxidative stress, ↓ET-1	Storniolo et al. (2014)
Nothofagin	Flavonoid phloretin	Endothelium-dependent vasodilation	*In vitro:* Perfused rat kidney	↑NOS ↓Perfusion pressure via NO and KCa1.1 channels	Marques et al. (2020)
Oligonol	Polyphenol	Hyperglycemia-induced endothelial dysfunction	*In vitro:* Endothelial cells	↑NO, ↑eNOS, ↑Akt, ↑p38 MAPK pathways activation, ↓PKC epsilon	Du et al. (2001)
		High-glucose condition	*In vitro:* Porcine aortic endothelial cells139	↑eNOS, ↑NO	Zhang et al. (2010)
Provinols	Polyphenol	Endothelial dysfunction and cardiac performance	*In vivo*: Zucker fatty rats	↑Endothelium-dependent relaxation, ↑NO, ↑eNOS, ↑cardiac performance, ↑Cardiac output, ↑left ventricular fractional shortening ↓Glucose, ↓triglycerides ↓Superoxide anion	Agouni et al. (2009)
		L-NAME-treated	*In vivo*: L-NAME-treated rats	↓Myocardial fibrosis, ↑NO, ↑Endothelium-dependent relaxation, ↓Aortic contractility	Bernátová et al. (2002)
Pyridoindole derivative SMe1EC2	Pyridoindole	Cardiovascular disorders related to metabolic syndrome	*In vivo:* Hypertriacylglycerolemic rats	↑eNOS3 ↑NO, ↑Left ventricular function, ↑Coronary flow ↓Inflammation, ↓Sensitivity to electrically induced ventricular fibrillation	Salvaras et al. (2021)
Resveratrol	Polyphenol	Ischemia-reperfusion injury in diabetic rats	*In vivo:* Diabetic rat model	↑NO/HO-1, ↑VEGF, ↑MnSOD; ↑Trx-1 ↓Glucose ↓Infarct size, ↓Cardiomyocyte apoptosis	Thirunavukkarasu et al. (2007)
		Diabetes	*In vitro:* Thoracic aorta, mesenteric arteries and corpora cavernosa of Rats	↑NO protected corpora cavernosa	Boydens et al. (2016)
		Metabolic syndrome-induced endothelial dysfunction	*In vivo:* Hypertensive rats	↑eNOS ↓Blood pressure, ↓Plasma triglycerides, ↓Insulin levels; ↓ET-1 secretion	Leibowitz et al. (2014)
		Hypercholesterolemic rabbit	*In vivo*: Rabbit model	↑NO ↑Endothelial function, ↓ET-1	Zou et al. (2003)
		Kidney failure in malignant hypertension	*In vivo:* Hypertensive rats	↓Oxidative damage, ↑Antioxidant defenses ↑NO Renoprotective	Grujić-Milanović et al. (2022)
		Renal ischemia-reperfusion injury	*In vivo:* Rats	↑ NO ↓Oxidative stress, ↓Renal dysfunction	Chander and Chopra (2005)
Resveratrol Butyrate Monoester	Polyphenol	Adenine-induced kidney dysfunction	*In vivo:* Hypertensive rats	↑NO, ↑Renin-angiotensin system, ↑Gut microbiota ↓Oxidative stress	Tain et al. (2023)

ACh, Acetylcholine; AChE, acetylcholinesterase; Akt, Protein Kinase B; BMI, body mass index; BP, blood pressure; cGMP, Nitric Oxide-cyclic Guanosine Monophosphate-Potassium channel; EDR, Endothelium-Dependent Relaxation; eNOS, endothelial nitric oxide synthase; eNOS, endothelial nitric oxide synthase; ET-1, Endothelin-1; GRP, Glucose-Regulated Protein; HO-1, Heme Oxygenase 1; HR, heart rate; HTN, hypertension; iNOS, inducible nitric oxide synthase; MnSOD, manganese superoxide dismutase; NF-κB, Nuclear Factor kappa B; NO, nitric oxide; NOS, nitric oxide synthase; NT, norepinephrine transporter; OS, oxidative stress; p38 MAPK, p38 Mitogen-Activated Protein Kinases; p53, Tumor Protein p53; PI3K, Phosphoinositide 3-Kinase; PKC; epsilon, Protein Kinase C epsilon; RAS, Renin-Angiotensin System; TG, triglycerides; TGF-β, transforming growth factor beta; TNF-α, tumor necrosis factor alpha; Trx-1, Thioredoxin-1; UCr, Urinary Creatinine; VEGF, vascular endothelial growth factor; VR, vascular resistance; XO, xanthine oxidase.

### 4.2 Phytoextracts

Several studies have reported that plant extracts modulate the L-arginine/NO/cGMP/K_ATP_ pathway in metabolic disorders (Table 5).

**TABLE 5:** Phytoextracts modulating the L-arginine/NO/cGMP/K_ATP_ pathway in metabolic disorders.

Extract	Type	Methods	*In vitro*/*In vivo* models	Mechanisms and outcomes	References
Alcohol-free alibernet red wine	Extract	Metabolic syndrome-induced inflammation High cholesterol diet	*In vivo:* Wistar Kyoto and SHR rats	↑NO, ↑Endothelial function ↓ET-1, ↓iNOS, ↓NF-κB, ↓Inflammatory response, ↓Flow-mediated dilation	Janega et al. (2014)
*Caralluma fimbriata* wall	Extract	High-fat diet-induced metabolic syndrome	*In vivo:* Obese mice	↑eNOS, ↑Acetylcholine, ↑Vascular function ↓Stress markers, ↓Nitrotyrosine, ↓GRP78, ↓Fat mass	Thunuguntla et al. (2024)
*Crinum zeylanicum* (L.) L	Extract	Hypertension and renal dysfunction	*In vivo:* Hypertensive rats	↑NO, ↑Renal function ↑Urinary creatinine ↓Blood pressure, ↓Heart rate	Ndjenda Ii et al. (2021)
*Gomphrena portulacoides* (A.St.-Hil.) T. Ortuño and Borsch [Amaranthaceae] (syn. Blutaparon portulacoides)	Extract	Hypertension and renal dysfunction	*In vivo:* Spontaneously hypertensive rats	↑NO/cGMP/K^+^ channel, ↑Cardioprotective ↑Diuretic activity ↑Renal function ↓Hypertension ↓Oxidative stress	Terço Leite et al. (2022)
Grape	Extract	Acute kidney injury	*In vivo:* Wild-type and eNOS-deficient mice *In vitro:* Cultured endothelial cells	↑PI3K/Akt/eNOS pathway activation, ↑Renal function, ↓Inflammatory markers	Ohkita et al. (2019)

ACh, Acetylcholine; AChE, acetylcholinesterase; Akt, Protein Kinase B; BMI, body mass index; BP, blood pressure; cGMP, Nitric Oxide-cyclic Guanosine Monophosphate-Potassium channel; EDR, Endothelium-Dependent Relaxation; eNOS, endothelial nitric oxide synthase; eNOS, endothelial nitric oxide synthase; ET-1, Endothelin-1; GRP, Glucose-Regulated Protein; HO-1, Heme Oxygenase 1; HR, heart rate; HTN, hypertension; iNOS, inducible nitric oxide synthase; MnSOD, manganese superoxide dismutase; NF-κB, Nuclear Factor kappa B; NO, nitric oxide; NOS, nitric oxide synthase; NT, norepinephrine transporter; OS, oxidative stress; p38 MAPK, p38 Mitogen-Activated Protein Kinases; p53, Tumor Protein p53; PI3K, Phosphoinositide 3-Kinase; PKC; epsilon, Protein Kinase C epsilon; RAS, Renin-Angiotensin System; TG, triglycerides; TGF-β, transforming growth factor beta; TNF-α, tumor necrosis factor alpha; Trx-1, Thioredoxin-1; UCr, Urinary Creatinine; VEGF, vascular endothelial growth factor; VR, vascular resistance; XO, xanthine oxidase.

The extract *Caralluma fimbriata* Wall. [Apocynaceae] significantly reduces weight gain and fat mass in mice on a high-fat diet, improving vascular function and enhancing acetylcholine-mediated relaxation in aortic rings. Mechanistically, this extract increased eNOS expression while decreasing stress markers, such as nitrotyrosine and GRP78 (Thunuguntla et al., 2024).

The aqueous and methanol extracts of leaves of *Crinum zeylanicum* (L.) L. [Amaryllidaceae] exhibited the highest levels of polyphenols and flavonoids. Treatment with these extracts significantly lowered blood pressure and heart rate in hypertensive rats, while also enhancing renal function by increasing NO levels and urinary creatinine (Ndjenda Ii et al., 2021).

Research has shown that an aqueous extract of *Gomphrena portulacoides* (A.St.-Hil.) T. Ortuño and Borsch [Amaranthaceae] (syn. *Blutaparon portulacoides*) at a dose of 300 mg/kg significantly lowers blood pressure in spontaneously hypertensive rats. The primary mechanism of action involves activation of the NO/cGMP/K_ATP_ Channel Pathway, which enhances diuretic activity. Furthermore, the extract not only improves renal function but also reduces markers of oxidative stress, thereby contributing to its cardioprotective effects (Terço Leite et al., 2022).

A study focusing on grape extract (administered intravenously at 0.1 and 1 mg/kg) demonstrated its ability to completely inhibit the expression of several inflammatory markers in cultured endothelial cells while also stimulating the PI3K/Akt/eNOS signaling pathway. *In vivo* experiments demonstrated that administering grape extract before ischemia induction significantly improved renal function in wild-type mice with acute kidney injury. At the same time, no such effect was observed in eNOS-deficient mice (Ohkita et al., 2019). Chander et al. also highlighted the essential role of NO in mediating the renoprotective benefits of resveratrol (Chander and Chopra, 2005).

## 5 Phytochemicals and phytoextracts modulating the L-arginine/NO/cGMP/K_ATP_ pathway for the treatment of pain management

Recent research has highlighted various phytochemicals as modulators of pain via L-arginine/NO/cGMP/K_ATP_ (Table 6).

**TABLE 6:** Phytochemicals modulating the L-arginine/NO/cGMP/K_ATP_ pathway in pain.

Compound(s)	Class	Methods	*In vitro*/*In vivo* models	Mechanisms and outcomes	References
Astaxanthin	Carotenoid	Formalin test administered intraperitoneally	*In vivo*: Male Mice	Significant antinociceptive effect through L-arginine/NO/cGMP/K_ATP_ pathway	Mohammadi et al. (2021)
Berberine	Alkaloid	Formalin test	*In vivo*: Rats	Antinociceptive effect mediated via NO/cGMP/K_ATP_ channel pathway	Rahemi et al. (2023)
Cuminaldehyde	Terpenoid	Acetic acid-induced writhing tests CCI model hot plate, formalin, and acetic acid tests	*In vivo:* Mice	↓ Allodynia, ↓hyperalgesia ↓ Pain via opioid receptors and L-arginine/NO/cGMP signaling pathway, ↓Inflammatory cytokines	Koohsari et al. (2020)
Cuminic alcohol	Monocyclic terpenoid	Hot plate, formalin, and acetic acid tests	*In vivo:* Mice	Opioid receptor activation, modulation of the L-arginine/NO/cGMP signaling pathway, ↓Inflammatory cytokines	Sheikholeslami et al. (2021)
Diosgenin	Steroidal saponin	Formalin and hot plate models	*In vivo:* Rats	Analgesic effect via NO/cGMP/K_ATP_ pathway. ↓Neuropathic pain, ↓Pro-inflammatory cytokines, ↓Oxidative stress through the modulation of the p38 MAPK and NF-κB signaling pathways	Zhao et al. (2017)
Ellagic acid	Polyphenolic compound	Formalin test	*In vivo:* Rats	Central and peripheral antinociceptive effect via NO and cGMP signaling, L-arginine/NO/cGMP/K_ATP_	Taghi Mansouri et al. (2013), Ghorbanzadeh et al. (2014), Mansouri et al. (2014)
Ferulic acid	Phenolic acid	Peripheral analgesic assay	*In vivo:* Mice	Peripheral analgesia via K_ATP_ and cGMP pathway activation	Kaşik et al. (2019)
Isopulegol	Monoterpenoid	Pain models	*In vivo:* Mice	Antinociceptive effect via NO-cGMP/K_ATP_, muscarinic, and opioid receptors	Andrade Próspero et al. (2018)
α-Mangostin	Xanthone	Opioid and vanilloid receptor assays	*In vivo:* Rodent model	Anti-inflammatory, Antioxidant, Antidiabetic, Anticancer activities, Peripheral and central pain-relieving effects, Modulation of the glutamatergic system and the l-arginine/NO/cGMP pathway, influences PKC and K_ATP_ pathways	Sani et al. (2015)
Naringenin	Flavonoid	Pain model	*In vivo*: Male rats	Analgesic effect mediated by l-arginine/NO/cGMP/PKG/K_ATP_ and Nrf2/HO-1 pathways Antioxidant, anti-inflammatory	Manchope et al. (2016)
Rutin	Bioflavonoid	Formalin-induced pain model	*In vivo:* Male NMRI mice	Antinociceptive effect via L-arginine/NO/cGMP/K_ATP_ pathway and opioid receptor activation Antioxidant anti-inflammatory vascular protective effects	Fayazzadeh et al. (2024)
α-Terpineol	Monoterpene	Pain and cardiovascular models	*In vivo:* Rats	Vasorelaxation and analgesic effects via the NO-cGMP pathway L-NAME significantly reduced the hypotensive effects ↑NO	Ribeiro et al. (2010)
Thymoquinone	Quinone	Pain modulation pathway analysis	*In vivo:* Rats	L-arginine/NO/cGMP/K_ATP_ channel pathway activation	Parvardeh et al. (2018)
Tingenone	Triterpenoid	PGE_2_-induced hyperalgesia	*In vivo*: Rat paw pressure test	↓ Pain perception	de Carvalho Veloso et al. (2015)
Zerumbone	Sesquiterpenoid	Chronic constriction injury (CCI) model	*In vivo:* Mice	Antihyperalgesic, Antiallodynic, Antimicrobial, Anti-inflammatory, Antioxidant, Anticancer Pain relief via ATP L-arginine/NO/cGMP/PKG/K_ATP_	Zulazmi et al. (2017)

ALD, alcoholic liver disease; ATP, adenosine triphosphate; CB2, Cannabinoid Receptor Type 2; CBRs, Cannabinoid Receptors; cGMP, cyclic guanosine monophosphate; EOS, endocannabinoid system; GABAergic, Gamma-Aminobutyric Acid; HO-1, Heme Oxygenase 1; HTN, hypertension; IL-1β, Interleukin-1; beta; IL-2, Interleukin-2. K_ATP_, ATP-Sensitive Potassium Channels; NO, nitric oxide; Nrf2, Nuclear Factor Erythroid 2-Related Factor 2; PKG, Protein Kinase G; sGC, soluble guanylate cyclase; TNF-α, tumor necrosis factor alpha; TRPV, transient receptor potential vanilloid.

The intricate interplay between phytochemicals and biological pathways has attracted increasing attention in pain management (Takeda et al., 2024). Among the various signaling mechanisms, the L-arginine/NO/cGMP/K_ATP_ pathway emerges as a crucial target for therapeutic intervention. Phytochemicals, bioactive compounds derived from plants, possess the potential to modulate this pathway (Table 5; Figure 3).

### 5.1 Phytochemicals

Rutin, also known as quercetin-3-*O*-rutinoside or rutoside, is a multifunctional bioflavonoid recognized for its wide-ranging health benefits, particularly its antioxidant, anti-inflammatory, and vascular-protective effects (Madkour et al., 2024). In previous studies, we demonstrated that rutin exhibits a dose-dependent antinociceptive effect, highlighting the involvement of the L-arginine/NO/cGMP/K_ATP_ channel-signaling pathway and the activation of opioid receptors. The use of antagonists or inhibitors that target specific elements of these signaling pathways—such as L-NAME, which inhibits NO synthase, and glibenclamide, which blocks K_ATP_ channels—significantly diminishes the analgesic effectiveness of rutin. Conversely, administering S-nitroso-N-acetylpenicillamine (SNAP), a NO donor, enhances the analgesic effects of rutin in both phases of the formalin test, indicating that NO plays a supportive role in its antinociceptive properties (Fayazzadeh et al., 2024).

Furthermore, we found that naringenin, another flavonoid known for its antioxidant and anti-inflammatory properties, may serve as an antinociceptive agent by acting through opioid receptors and the l-arginine/NO/cGMP/K_ATP_ pathway (Moradi et al., 2021). Similarly, research conducted by Manchope et al. found that naringenin’s antinociceptive effects are mediated by the stimulation of the NO-cGMP-PKG-K_ATP_ signaling pathway, which also interacts with the Nrf2/HO-1 pathway (Manchope et al., 2016).

In another study, astaxanthin, a carotenoid recognized for its potent antioxidant properties (Hashemi et al., 2024; Rastinpour et al., 2025), was investigated in relation to the L-arginine/NO/cGMP/K_ATP_ channel signaling pathway and its contribution to antinociceptive effects. Our findings revealed that a dosage of 10 mg/kg of astaxanthin, administered intraperitoneally, produced the most significant antinociceptive effect during both the acute and inflammatory phases of the formalin test (Mohammadi et al., 2021).

Ellagic acid, a natural polyphenol compound, exhibited both central and peripheral antinociceptive effects. Studies have shown that its analgesic properties are dose-dependent and involve multiple pathways, including the modulation of NO and cGMP signaling pathways (Taghi Mansouri et al., 2013; Mansouri et al., 2014). Ghorbanzadeh et al. demonstrated that ellagic acid exhibits local analgesic effects in rats in a dose-dependent manner during both the early and late phases of the formalin test, highlighting its action through the L-arginine/NO/cGMP/K_ATP_ pathway (Ghorbanzadeh et al., 2014).

Rahemi et al. concluded that the L-arginine/NO/cGMP/K_ATP_ channel pathway is crucial for the local antinociceptive effects of berberine, an isoquinoline alkaloid known for its diverse pharmacological properties, particularly during the second phase of the rat formalin test (Rahemi et al., 2023).

Zerumbone is a bioactive compound derived from the rhizomes of *Zingiber zerumbet* (L.) Roscoe ex Sm. [Zingiberaceae], commonly known as bitter ginger (Devkota et al., 2021). This sesquiterpene has garnered attention for its diverse pharmacological properties, including antimicrobial, anti-inflammatory, antioxidant, and anticancer activities (Ibáñez et al., 2022). Zulazmi et al. demonstrated that the antiallodynic and antihyperalgesic effects of zerumbone, administered at 10 mg/kg via intraperitoneal injection in the chronic constriction injury (CCI) model, are primarily mediated through the L-arginine-NO/cGMP/PKG/K_ATP_ channel pathways (Zulazmi et al., 2017).

Another significant bioactive compound found in *C. cyminum* is cuminaldehyde, which has demonstrated notable analgesic properties. Research indicates that cuminaldehyde effectively reduces pain responses across various models, including the hot-plate, formalin, and acetic acid tests. Its analgesic effects appear to be primarily mediated through the activation of opioid receptors and the L-arginine/NO/cGMP signaling pathway. In the CCI model, cuminaldehyde not only alleviated allodynia and hyperalgesia at doses ranging from 25 to 100 mg/kg, but it also significantly decreased serum levels of the inflammatory cytokines TNF-α and IL-1β (Koohsari et al., 2020).

Cuminic alcohol, or 4-isopropylbenzyl alcohol, is a monocyclic terpenoid derived from medicinal plants such as *Cuminum cyminum* L. and *Elwendia persica* (Boiss.) Pimenov and Kljuykov [Apiaceae]. The analgesic effects of cuminic alcohol were assessed across several animal pain models, including hot plate, formalin, and acetic acid tests. The findings revealed that cuminic alcohol at doses of 200 and 400 mg/kg effectively alleviated pain by activating opioid receptors, modulating the L-arginine/NO/cGMP signaling pathway, and exerting anti-inflammatory actions, specifically by reducing levels of the inflammatory cytokines TNF-α and IL-1β (Sheikholeslami et al., 2021).

In formalin and hot-plate models of nociception in rats, diosgenin, a steroidal saponin, exhibited a significant analgesic effect at 50 mg/kg, primarily via the NO/cGMP/K_ATP_ pathway (Meroka, O.J. et al., 2023). Additionally, Zhao et al. previously showed that diosgenin alleviates neuropathic pain by reducing the levels of pro-inflammatory cytokines (TNF-α, IL-1β, IL-2) and decreasing oxidative stress through the modulation of the p38 MAPK and NF-κB signaling pathways (Zhao et al., 2017).

Ferulic acid is a naturally occurring hydroxycinnamic acid derivative and phenolic compound (Mathew and Abraham, 2006). Research has demonstrated that ferulic acid at a dose of 80 mg/kg exerts a significant peripheral analgesic effect, primarily through activation of K_ATP_ channels and, to a lesser extent, via the cGMP pathway (Kaşik et al., 2019).

Isopulegol is a monoterpenoid alcohol celebrated for its refreshing, cool, and herbal aroma. Research on animals has demonstrated that oral administration of isopulegol exhibits significant antinociceptive properties in mice, acting through multiple mechanisms. These include the activation of opioid receptors, K_ATP_ channels, muscarinic receptors, and the NO/cGMP pathway (Andrade Próspero et al., 2018).

α-mangostin is a natural xanthone predominantly found in the pericarp of the mangosteen fruit (*Garcinia mangostana* L. [Clusiaceae]). It has garnered significant attention for its diverse pharmacological properties, including anti-inflammatory, antioxidant, antidiabetic, and anticancer activities (Alam et al., 2023). It has been reported that α-mangostin exhibits both peripheral and central pain-relieving effects by targeting multiple pathways. The compound interacts with opioid receptors and vanilloid receptors, which are integral to pain perception. It interacts with opioid and vanilloid receptors involved in pain perception. Additionally, it modulates the glutamatergic system and the L-arginine/NO/cGMP pathway, which are vital for pain signaling, and influences PKC and K_ATP_ pathways (Sani et al., 2015).

Alpha-terpineol is a monoterpene found in the essential oils of several aromatic plant species. Alpha-terpineol induces dose-dependent hypotension and tachycardia in normotensive rats, with its hypotensive effects significantly reduced by L-NAME. In isolated mesenteric artery rings, alpha-terpineol causes concentration-dependent vasorelaxation, which is diminished when the endothelium is removed or when NOS is inhibited. Ribeiro et al. concluded that the cardiovascular effects of alpha-terpineol are primarily mediated by endothelium-dependent NO release and activation of the NO/cGMP pathway (Ribeiro et al., 2010).

Thymoquinone, a bioactive compound derived from the seeds of *Nigella sativa* L. [Ranunculaceae] (black cumin), has been investigated for its potential analgesic properties. The signaling pathways involved in its antinociceptive effects, particularly the L-arginine/NO/cGMP/K_ATP_ channel pathway, played a significant role in mediating pain responses in both central and peripheral systems (Parvardeh et al., 2018).

Tingenone is a triterpenoid compound primarily derived from *Salacia impressifolia* (Miers) A.C. Sm. [Celastraceae] and *Euonymus tingens* Wall. [Celastraceae] (Alvarenga and Ferro, 2006). Administration of tingenone (200 µg/paw) into the right hind paw produced a local antinociceptive effect. Tingenone induces peripheral antinociception by activating the L-arginine/NO/cGMP pathway, enhancing analgesic effects without side effects (de Carvalho Veloso et al., 2015). It also activates cannabinoid receptors, particularly CB2, contributing to its multifaceted analgesic properties (Veloso et al., 2018). Additionally, its interaction with the opioidergic system suggests that tingenone may enhance endogenous opioid signaling, further supporting its potential as an analgesic (Veloso et al., 2014).

### 5.2 Phytoextracts

There are studies on phytoextracts that highlight their modulatory effects on the L-arginine/NO/cGMP/K_ATP_ pathway in pain (Table 7).

**TABLE 7:** Phytoextracts modulating the L-arginine/NO/cGMP/K_ATP_ pathway in pain.

Compound(s)	Type used	Methods	*In vitro*/*In vivo* models	Mechanisms and outcomes	References
*Bupleurum falcatum* L	Essential oil	Formalin-induced paw licking model, Cervical spinal cord contusion	*In vivo:* Male mice	↓TNF-α, ↓IL-1β, ↓IL-2, Anti-nociceptive, L-arginine/NO/cGMP/K_ATP_ channel pathway, interactions with opioid, PPAR, cannabinoid receptors. ↑ TNF-α, ↑IL-1β, ↑IL-2 Anti-allodynic and anti-nociceptive effects, Activated the L-arginine/NO/cGMP/K_ATP_ channel pathway, Interaction with opioid, PPA, and cannabinoid receptors ↓TNF-α, ↓IL-1β, ↓IL-2	Ahmadimoghaddam et al. (2021)
*Centaurea benedicta* (L.) L	Methanolic extract	Tail-flick and formalin tests	*In vivo*: Rats	Antinociceptive, mediated through the L-arginine/NO/cGMP/K_ATP_ channel pathway, Opioidergic mechanisms	Ahmadimoghaddam et al. (2020)
*Dahlstedtia araripensis* (Benth.) M.J. Silva and A.M. G. Azevedo	Protein extract	Carrageenan-induced hypernociception model	*In vivo*: Swiss mice	Hypernociception alleviation via NO/cGMP/K_ATP_ pathway	Assreuy et al. (2020)
		Paw edema and peritonitis models	*In vivo:* Mice	↓Paw edema, ↓BK, ↓PGE2, ↓TNF-α, ↓leukocyte rolling and adhesion	Pires et al. (2016)
*Lotus corniculatus* L	Essential oil	Formalin-induced paw licking model, Cervical spinal cord contusion	*In vivo:* Rats	Modulation of opioid, TRPV, dopamine, cannabinoid mechanisms NO-cGMP-K^+^ channel, ↓TNF-α, ↓IL-1β, ↓IL-2	Jabbari et al. (2024)
*Muntingia calabura* L	Methanolic extract	Acetic acid	*In vivo:* Mice	Antinociceptive effects, ↑NO, ↑sGC/cGMP/PKG signaling	Zakaria et al. (2006)
Green pomegranate peel extract + Aspirin	Extract	Formalin test	*In vivo:* Male rats	Antinociceptive, L-arginine/NO/cGMP pathway, antioxidant activity	Guerrero-Solano et al. (2021)
*Pentanema britannica* (L.) D.Gut.Larr., Santos-Vicente, Anderb., E.Rico and M.M.Mart.Ort. (syn*. Inula britannica* L.) and Patuletin	Flower Essential oil	Acetic acid-induced writhing, Tail-flick formalin-induced paw licking model, Glutamate-induced paw licking	*In vivo:* Mice	Analgesic, Glutamatergic system modulation, Opioid receptor activation, L-arginine/NO/cGMP/K_ATP_ signaling pathway	Zarei et al. (2018)
*Rhus virens* Lindh. ex A.Gray	Flavonoid-rich extract	Formalin test, Local edema	*In vivo*: Mice	Antinociceptive, Anti-inflammatory, Antioxidant properties, Opioidergic, GABAergic, NO/GMPc/K_ATP_ activation, ↑ 1,2,3,4,6-penta-O-galloyl-glucopyranose	Vargas-Ruiz et al. (2023)
*Salvia rosmarinus* Spenn	Essential oil	Formalin test	*In vivo:* Mice	Analgesic effect mediated by NO/cGMP/K_ATP_ pathway	Hajhashemi et al. (2023)
*Thymus persicus* (Ronniger ex Rech.f.) Jalas	Essential oil	Formalin test, cervical spinal cord contusion	*In vivo:* Mice	Antinociceptive effect, L-arginine/NO/cGMP/K_ATP_ signaling pathway, Adrenergic, opioid, serotonin receptor interaction, NO/cGMP pathway anti-neuropathic mediated by anti-inflammatory	Abed et al. (2022)
*Trifolium resupinatum* L	Leaves essential oil	Formalin test, Cervical spinal cord hemi-contusion	*In vivo:* Male rats	Activation of TRPV, GABA, and cannabinoid receptors. Anti-neuropathic effects via ↓TNF-α, ↓IL-1β, ↓IL-2, ↓Stat-3	Jabbari et al. (2025)

ALD, alcoholic liver disease; ATP, adenosine triphosphate; CB2, Cannabinoid Receptor Type 2; CBRs, Cannabinoid Receptors; cGMP, cyclic guanosine monophosphate; EOS, endocannabinoid system; GABAergic, Gamma-Aminobutyric Acid; HO-1, Heme Oxygenase 1; HTN, hypertension; IL-1β, Interleukin-1; beta; IL-2, Interleukin-2. K_ATP_, ATP-Sensitive Potassium Channels; NO, nitric oxide; Nrf2, Nuclear Factor Erythroid 2-Related Factor 2; PKG, Protein Kinase G; sGC, soluble guanylate cyclase; TNF-α, tumor necrosis factor alpha; TRPV, transient receptor potential vanilloid.

The cervical spinal cord contusion and formalin-induced paw-licking models have been employed to evaluate the antinociceptive effects of essential oil from *Bupleurum falcatum* L. [Apiaceae], commonly known as Chinese Thoroughwax, specifically targeting neurogenic, inflammatory, and neuropathic pain. Recent findings demonstrate that 100 mg/kg of this essential oil exhibits significant antinociceptive properties through the L-arginine/NO/cGMP/K_ATP_ channel pathway, as well as interactions with opioid, peroxisome proliferator-activated receptor (PPAR), and cannabinoid receptors. Additionally, these results suggest that it effectively alleviates allodynia by modulating TNF-α, IL-1β, and IL-2 expression in the rat spinal cord (Ahmadimoghaddam et al., 2021).

*Centaurea benedicta* (L.) L. (syn. *Cnicus benedictus*) [Asteraceae], commonly known as Blessed Thistle, is renowned for its medicinal properties (Al-Snafi, 2016). A study found that the methanolic extract of *C.benedicta*, administered at 150 and 300 mg/kg (i.p.), along with its primary constituent, cnicin (30 mg/kg), demonstrated significant antinociceptive effects. These effects were mediated through the L-arginine/NO/cGMP/K_ATP_ channel pathway and opioidergic mechanisms, as evidenced by the results from the tail-flick and formalin tests (Ahmadimoghaddam et al., 2020).

The lectin isolated from *Dahlstedtia araripensis* (Benth.) M.J. Silva and A.M. G. Azevedo (syn. *Lonchocarpus araripensis*) [Fabaceae] has been the subject of numerous studies due to its notable biological activities, particularly in inflammation (Napimoga et al., 2007; Pires et al., 2016). Assreuy et al. examined the involvement of the NO pathway in the antinociceptive effects of lectin using a carrageenan-induced hypernociception model in Swiss mice. Their findings demonstrated that lectin administration significantly alleviated hypernociception, with the most pronounced effect observed at a dose of 10 mg/kg. The antinociceptive action of lectin was inhibited by various pharmacological blockers, suggesting the engagement of multiple signaling pathways, particularly the L-arginine/NO/cGMP/K_ATP_ pathway (Assreuy et al., 2020).

An animal study investigated the antiallodynic and antinociceptive effects of the essential oil of leaves of *Lotus corniculatus* L. [Fabaceae] (LCEO) using both a cervical contusion injury model and a formalin model in rats. The results indicated that both LCEO and its primary constituent, oleanolic acid (OA), significantly alleviated acute pain. This analgesic effect was primarily attributed to the modulation of several receptor systems, including vanilloid (TRPV) receptors, cannabinoid receptors, opioid receptors, and the NO/cGMP/K_ATP_ pathway (Jabbari et al., 2024).

*Muntingia calabura* L. [Muntingiaceae], commonly known as Jamaican cherry or strawberry tree, is gaining recognition for its therapeutic potential, particularly in cancer prevention and the treatment of gastrointestinal disorders (Mahmood et al., 2014). Investigations into the methanol extract of *Muntingia calabura* have highlighted the involvement of the L-arginine/NO/cGMP pathway in mediating its antinociceptive effects (Zakaria et al., 2006). Shih also proposed that the cardiovascular effects of *M. calabura* are mediated by NO production and the activation of the sGC/cGMP/PKG signaling cascade (Shih, 2009).

*Pentanema britannica* (L.) D. Gut.Larr., Santos-Vicente, Anderb., E. Rico and M.M.Mart.Ort. (syn*. Inula britannica* L.). is a flowering plant belonging to the Asteraceae family. It is commonly known as British elecampane. Traditionally, various parts of *I. britannica* have been used in herbal medicine, primarily for their potential anti-inflammatory and antimicrobial properties. Patuletin is a flavonoid found in several plant species, including some in the *Pentanema* genus (Kim et al., 2024c). A study found that essential oil from P. britannica and its component, patuletin, significantly reduced nociceptive behaviors across various pain assessment tests. These included acetic acid-induced writhing, the tail-flick test, formalin-induced paw licking, and glutamate-induced paw licking. The observed analgesic effects are likely mediated by the modulation of glutamatergic systems, particularly involving both selective and non-selective opioid receptors. Furthermore, activation of the L-arginine/NO/cGMP/K_ATP_ signaling pathway appears crucial for eliciting these antinociceptive effects (Zarei et al., 2018).

A study indicated that the combination of green pomegranate peel extract and acetylsalicylic acid (aspirin) enhances pain relief (antinociceptive effects) through several mechanisms, including the l-arginine/NO/cGMP pathway, antioxidant activity, and high total phenol content (Guerrero-Solano et al., 2021).

*Rhus virens* Lindh. ex A. Gray [Anacardiaceae], commonly referred to as evergreen sumac. This plant exhibits notable antinociceptive, anti-inflammatory, and antioxidant properties, which may be attributed to its interactions with various biological pathways, including opioidergic and γ-aminobutyric acid (GABA)ergic systems, as well as NO and GMPc signaling pathways involving K_ATP_ channels. A key component believed to enhance these pharmacological effects is 1,2,3,4,6-penta-*O*-galloyl-glucopyranose, which plays a significant role in the plant’s bioactivity (Vargas-Ruiz et al., 2023).

Rosemary essential oil, derived from *Salvia rosmarinus* Spenn. (syn. *Rosmarinus officinalis* L.). [Lamiaceae] has gained attention for its potential therapeutic benefits, particularly in pain management and anti-inflammatory applications (Ghasemzadeh Rahbardar and Hosseinzadeh, 2020). Research indicates that the analgesic effect of rosemary essential oil in mice, particularly at a dose of 300 μL/kg, is likely mediated through the NO/cGMP/K_ATP_ signaling pathway (Hajhashemi et al., 2023).

*Thymus persicus* (Ronniger ex Rech. f.) Jalas [Lamiaceae], an endemic medicinal plant from Iran, has demonstrated significant potential in alleviating nociceptive and neuropathic pain behaviors in mice. This was evaluated using the formalin test and a cervical spinal cord contusion model. The study revealed that doses of 50, 100, and 150 mg/kg of *T. persicus* essential oil exhibit notable antinociceptive effects, mediated through the L-arginine/NO/cGMP/K_ATP_ signaling pathway and interactions with adrenergic, opioid, and serotonin receptors. Furthermore, the plant’s anti-neuropathic activity appears to be mediated by its anti-inflammatory properties (Abed et al., 2022).

Jabbari et al. reported that *Trifolium resupinatum* L. [Fabaceae] shows considerable promise as a natural therapeutic agent for the management of both nociceptive and neuropathic pain. The analgesic effects of this plant are mediated by intricate interactions involving NO signaling, TRPV channel activation, and modulation of GABAergic pathways (Jabbari et al., 2025).

## 6 Challenges with the bioavailability of phytochemicals

One of the key challenges in harnessing phytochemicals for clinical applications is their low bioavailability—the extent and rate at which active ingredients are absorbed and become available at their sites of action. Many phytochemicals are lipophilic and poorly water-soluble, limiting their absorption in the gastrointestinal tract. Additionally, these compounds may be rapidly metabolized or excreted before they can enter systemic circulation, diminishing their therapeutic efficacy. Furthermore, certain phytochemicals can degrade under physiological conditions, further compromising their potential benefits (Hu et al., 2023; Nicolescu et al., 2023).

To address these challenges, researchers are exploring various innovative strategies. Advanced delivery systems, such as nanoparticles, can encapsulate phytochemicals, protecting them from degradation and enhancing their absorption. Lipid-based formulations, including liposomes and solid lipid nanoparticles, enhance solubility and facilitate lymphatic transport, thereby increasing bioavailability. Modifying the chemical structure of phytochemicals can enhance their stability and solubility, potentially improving efficacy. Combining phytochemicals with other compounds, such as adjuvants, may further enhance absorption or produce synergistic effects that improve therapeutic outcomes, particularly in the context of NO production. Additionally, incorporating phytochemical-rich foods into daily diets provides a natural means to increase intake and optimize the bioavailability of these beneficial compounds, thanks to synergistic interactions with other nutrients (Imam et al., 2021; Haines et al., 2024).

By employing these diverse strategies, we can enhance the clinical applicability of phytochemicals and more effectively harness their potential health benefits.

## 7 Discussion and conclusion

The L-arginine/NO/cGMP/K_ATP_ pathway operates as an integrated signaling network with extensive crosstalk among multiple molecular mediators. L-arginine, the substrate for NOS, is enzymatically converted to NO, which acts as a diffusible messenger influencing vascular tone, nociception, and metabolic processes. NO activates sGC, elevating cGMP levels, which, in turn, activate PKG. PKG modulates downstream effectors, including the opening of K_ATP_ channels, affecting cellular excitability and cytoprotection.

Phytochemicals navigate this system’s complexity by exerting pleiotropic effects and through multiple mechanisms. Such plant-derived active compounds enhance NO bioavailability directly by upregulating eNOS expression and activity via phosphorylation cascades involving PI3K/Akt signaling, thereby facilitating sustained NO production. The antioxidant properties of phytochemicals reduce ROS that degrade NO, thereby improving its signaling efficacy. They modulate cGMP levels not only via NO synthesis but also by influencing PDE activity, which degrades cGMP. Phytochemicals impact K_ATP_ channel function both by direct activation and by modulating upstream signals that regulate these channels, thereby contributing to vascular and neuronal effects on vasodilation and pain modulation. Additional crosstalk with other signaling pathways, including MAPK, NF-κB, and calcium-dependent mechanisms, facilitates broader anti-inflammatory, anti-apoptotic, and metabolic regulatory outcomes. In the nervous system, the pathway interacts with NMDA receptor activity and neuropeptide release, thereby modulating pain signaling at both peripheral and central sites.

Thus, phytochemical modulation of the L-arginine/NO/cGMP/K_ATP_ pathway should be conceptualized as a dynamic, context-dependent interaction with multiple feedback and feedforward loops rather than a simple linear cascade. This complexity enables phytochemicals to exert systemic therapeutic benefits in cardiovascular, metabolic, and pain-related disorders, often with generally favorable safety profiles compared to traditional pharmacological agents.

Despite the promising findings, challenges related to the bioavailability, dosing, and standardization of these phytochemicals remain significant barriers to their clinical application. Many bioactive compounds exhibit low solubility, rapid metabolism, and variability in preparation, which can hinder their ability to reach therapeutic concentrations at target sites. Innovative strategies, including advanced delivery systems, standardized formulations, and dietary modifications, are essential to enhance the bioavailability, efficacy, and safety of these compounds.

Future research should focus on the complex interactions between phytochemicals and the L-arginine/NO/cGMP/K_ATP_ pathway, particularly through well-designed clinical trials that evaluate optimal dosing, pharmacokinetics, and potential toxicity across diverse populations and disease states. By addressing these critical limitations, we can better harness the therapeutic potential of phytochemicals, paving the way for novel, effective, and safer interventions that target this key signaling pathway and improve health outcomes across a range of conditions.

## Glossary

β2-AR: Beta-2 adrenergic receptor
β-Gal: Beta-galactosidase
6-keto-PGF1α: 6-keto-prostaglandin F1 alpha
AC/cAMP/PKA: Adenylate cyclase/cyclic AMP/protein kinase A
ACE: Angiotensin-converting enzyme
ACh: Acetylcholine
AChE: Acetylcholinesterase
AhR-RGE: Aryl hydrocarbon receptor-regulated gene enhancement
Ahr-TN: Aryl hydrocarbon receptor translocated
AI: Atherogenic index
Akt: Protein kinase B
ALD: Alcoholic Liver Disease
ALDH: Aldehyde dehydrogenase
AMPK: AMP-activated protein kinase
Ang II: Angiotensin II
Ang II-CFP: Angiotensin II - Central Fusion Point
AP-1: Activator protein 1
ARNT: Aryl hydrocarbon receptor nuclear Translocator
AS: Atherosclerosis
ATP: Adenosine Triphosphate
ATPases: Adenosine triphosphatases
Bcl-2/Bax: B-cell Lymphoma 2/Bcl-2-associated X Protein
bFGF: Basic fibroblast growth factor
BMI: Body mass index
BP: Blood pressure
BRS: Baroreflex sensitivity
CaMKII: Calcium/calmodulin-dependent protein kinase II
CaMKKβ: Calcium/calmodulin-dependent protein kinase kinase beta
CAT: catalase
CB2: Cannabinoid Receptor
CBRs: Cannabinoid Receptors Type 2
CD39: Cluster of differentiation 39
CD73: Cluster of differentiation 73
CDR: Cognitive decline Rate
cGMP: Nitric oxide-cyclic guanosine monophosphate-potassium channel
CK: Creatine kinase
CLS: Capillary-like structures
cNOS: Constitutive nitric oxide synthase
CPK: Creatine phosphokinase
CS: Cholesterol sulfate
cTnI: Cardiac troponin I
CVP: Central venous pressure
CYP7A1: Cholesterol 7-alpha-hydroxylase
ECFC: Endothelial colony-forming cells
EDHF: Endothelium-derived hyperpolarizing factor
EDR: Endothelium-dependent relaxation
EDV: End diastolic volume
eNO: Endothelial nitric oxide
eNOS: Endothelial nitric oxide synthase
EOS: Endocannabinoid System
ERK1/2: Extracellular signal-regulated kinases 1/2
ET-1: Endothelin-1
GABAergic: Gamma-Aminobutyric Acid
GC: Guanylate cyclase
GRP: Glucose-regulated protein
GSH/GSSG: Reduced glutathione/glutathione disulfide ratio
GSH-Px: Glutathione Peroxidase
GSK-3β: Glycogen synthase kinase 3 Beta
**H**_**2**_ **O**_**2**_: Hydrogen peroxide
Hcy-ED: Homocysteine-endothelial dysfunction
HDL: High-density lipoprotein
HDL-C: High-density lipoprotein cholesterol
HO-1: Heme oxygenase 1
HR: Heart rate
HTN: Hypertension
ICAM-1: Intercellular adhesion molecule 1
IL-1β: Interleukin 1 beta
IL-6: Interleukin 6
iNOS: Inducible nitric oxide synthase
IP3R: Inositol 1,4,5-trisphosphate receptor
JNK: c-Jun N-terminal kinase
JNK1/2: c-Jun N-terminal kinases 1/2
**K**_**ATP**_: ATP-sensitive potassium channel
KCM: Potassium channel modulation
KDR: Kinase insert domain receptor
K-Ras: Kirsten rat sarcoma viral oncogene homolog
KV and KATP: Voltage-gated potassium and ATP-sensitive potassium channels
LDH: Lactate dehydrogenase
LDL: Low-density lipoprotein
LDL-C: Low-density lipoprotein cholesterol
LF and HF: Low frequency and high frequency components of power index
LPO: Lipid peroxidation
MAP: Mean arterial presure
MAPK: Mitogen-activated protein kinase
MDA: Malondialdehyde
MF: Myocardial Fibrosis
mitoKATP: Mitochondrial ATP-Sensitive Potassium Channel
MMP: Matrix metalloproteinase
MnSOD: Manganese superoxide dismutase
mRNA: Messenger Ribonucleic Acid
NADPH: Nicotinamide adenine dinucleotide phosphate
NF-κB: Nuclear factor kappa B
NLRP3: NLR family pyrin domain containing 3
NO: Nitric oxide
NOS: Nitric oxide synthase
NO: Nitric oxide
NOS2 and NOS3: Nitric oxide synthase 2 and 3
NOX4: NADPH oxidase 4
NQO1: NAD(P)H quinone dehydrogenase 1
NRC: Nuclear regulatory commission
Nrf2: Nuclear factor erythroid 2–related factor 2
NT: Norepinephrine Transporter
OS: Oxidative stress
p38 MAPK: p38 mitogen-activated protein kinases
p47phox: Phox subunit of NADPH oxidase
p53: Tumor protein p53
PE: Phenylephrine
PGI: Prostacyclin
PI3K: Phosphoinositide 3-kinase
PKC epsilon: Protein kinase C epsilon
PKG: Protein Kinase G
p-NF-κB: Phosphorylated nuclear factor kappa B
PTEN: Phosphatase and tensin homolog
RAS: Renin-angiotensin system
ROS: Reactive oxygen species
RS: Renal stenosis
SAL: Salinity
sGC: Soluble guanylate cyclase
SIPS: Stress-induced premature senescence
Smad2/3: SMAD family member 2/3
SOD: Superoxide dismutase
STCL: Short term cellular leukemia
TG: Triglycerides
TGF-β: Transforming growth factor beta
TGF-β1: Transforming growth factor beta 1
TNF-α: Tumor necrosis factor alpha
TNFR: Tumor necrosis factor receptor
TPE: Total protein excretion
TRPA1: Transient receptor potential ankyrin 1
Trx-1: Thioredoxin-1
TRPV: Transient Receptor Potential Vanilloid
TXB2: Thromboxane B2
UCr: Urinary creatinine
VCAM-1: Vascular cell adhesion molecule 1
VD: Vascular diameter
VEGF: Vascular endothelial growth factor
VOCC: Voltage-operated calcium channels
VP: Venous pressure
VR: Vascular resistance
XO: Xanthine oxidase
